# The AMIGa complex is at the crossroads of cytochrome *c* oxidase and ATP synthase biogenesis

**DOI:** 10.1038/s44319-026-00946-7

**Published:** 2026-10-06

**Authors:** Ulrik Pedroza-Dávila, Yolanda Camacho-Villasana, Miriam Vázquez-Acevedo, Madhurya Lutikurti, Diego González-Halphen, Alfredo Cabrera-Orefice, Xochitl Perez-Martinez

**Affiliations:** 1 Departamento de Genética Molecular, Instituto de Fisiología Celular, Universidad Nacional Autónoma de México, México City, México; 2 Research Institute for Medical Innovation, Radboud University Medical Center, Nijmegen, the Netherlands; 3 Functional Proteomics Center, Goethe University, Frankfurt am Main, Germany; 4 Institute of Biochemistry, Faculty of Medicine, Justus Liebig University, Giessen, Germany

**Keywords:** Membranes & Trafficking, Metabolism, Translation & Protein Quality

## Abstract

Cytochrome c oxidase (CIV) and ATP synthase are part of the oxidative phosphorylation machinery. Their modular, highly regulated assembly requires specific factors and is coordinated through a common intermediate, the Atco complex, composed of the Atp9 ring and Cox6. By high-throughput analysis, it was previously detected that Aim11 interacts with some CIV and ATP synthase subunits, but its relevance was unknown. In this work, we characterize the role of Aim11 in CIV and ATP synthase biogenesis. We find that the null mutant exhibits reduced respiratory growth and diminished CIV activity. Mitochondrial complexome profiling of the *Δaim11* mutant shows an accumulation of intermediates of the CIV and ATP synthase assembly modules. Aim11 forms a complex with three other proteins: Mtc3, Gep7, and Iai11, which we coin as AMIGa (Aim11-Mtc3-Iai11-Gep7 assembly complex). Aim11 interacts with the newly synthesized Atp9 ring, possibly mediated by the Atco complex. In addition, we identify the human protein TMEM242 as an Aim11 orthologue.

## Synopsis

The AMIGa complex is a novel inner mitochondrial heterotetramer involved in the assembly of complex IV and ATP synthase in *S. cerevisiae*.

- The AMIGa complex is formed by Aim11, Gep7, Iai11 and Mtc3.
- Loss-of-function of AMIGa complex components results in respiration-deficient cells mainly due to diminished CIV assembly.
- Aim11 interacts with the newly synthesized Atp9 ring, and in its absence, ATP synthase assembly intermediates accumulate.
- The role of the AMIGa complex might be related to the stability of the Atco complex, a common intermediate of complex IV and ATP synthase.
- TMEM242 is the human orthologue of Aim11.

## Abstract (web summary)

The AMIGa complex is a novel inner mitochondrial heterotetramer involved in the assembly of complex IV and ATP synthase in *S. cerevisiae*.

## Graphical abstract

**Figure:**
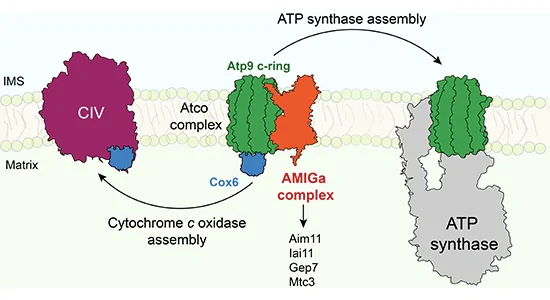

## Introduction

The oxidative phosphorylation system (OXPHOS) comprises the respiratory complexes and ATP synthase. On one hand, cytochrome *c* oxidase (complex IV, CIV) is the last electron acceptor of the mitochondrial respiratory chain and is responsible for donating electrons to molecular oxygen and contributing to the electrochemical potential. In *Saccharomyces cerevisiae*, it is composed of three core subunits (Cox1-3) whose genes are in the organelle’s genome (mtDNA) and nine accessory subunits (Cox4-9, Cox12, Cox13, and Cox26) encoded in nuclear genes. On the other hand, ATP synthase uses electrochemical potential to synthesize ATP from ADP and inorganic phosphate. It is composed of two domains, one hydrophilic (F_1_) and one membranal (F_0_) (Artika, 2019; He et al, 2018). In yeast, three subunits (Atp6, Atp8 and Atp9) are encoded in the mtDNA, and the rest in the nuclear genome. Due to their dual genetic origin, the assembly of CIV and ATP synthase subunits must be coordinated to ensure correct conformation and stoichiometry (Moretti-Horten et al, 2024). Additionally, their biogenesis is proposed to be modular, where specific subunits are associated independently and then assemble to produce the mature complex. In the case of CIV, each mtDNA-encoded subunit (Cox1, Cox2, and Cox3) is associated with a specific set of accessory subunits and assembly factors to form the three distinct modules (Su et al, 2014b; Franco et al, 2018; McStay et al, 2013b). In contrast, the F_1_ and F_0_ portions of ATP synthase are assembled independently on the mitochondrial matrix or the mitochondrial inner membrane, respectively (Franco et al, 2020b). However, it remains unknown how some of these biogenesis steps occur and which proteins are involved in the regulation of these processes. Initially, it was thought that these two processes were isolated (Pedroza-Dávila et al, 2026). Nevertheless, the group from Alexander Tzagaloff showed that Cox6 (from the Cox1 module of CIV) and the Atp9 ring (from ATP synthase F_0_ portion) form a common intermediate known as the Atco complex in yeast (Franco et al, 2020a). This intermediate is proposed to coordinate CIV and ATP synthase biogenesis and maintain a proper ratio between these OXPHOS complexes(Franco et al, 2023).

In this work, we characterized the mitochondrial protein Aim11 and unveiled its role in the assembly of complex IV and ATP synthase. Aim11 is a mitochondrial inner membrane protein of ~15 kDa with two transmembrane helices (Morgenstern et al, 2017). Aim11 loss was shown to be lethal in the absence of prohibitins (Osman et al, 2009), a multimeric multifunctional mitochondrial inner membrane scaffold that has been linked to mitochondrial quality control (Champsi and Hood, 2025). Additionally, a large-scale pulldown experiment showed that Aim11 physically interacts with some subunits and assembly factors of ATP synthase (α, β, δ, and ε) and CIV (Cox2, Cox20, and Cox6) (Morgenstern et al, 2017, Data ref: Morgenstern et al, 2017). However, the relevance of these interactions was not established.

Here, we found that Aim11 is necessary for the optimal assembly of complex IV, and to a lesser degree, ATP synthase. Its absence results in the accumulation of assembly intermediates of both complexes. The *Δaim11* mutant showed a respiratory defect, which is suppressed by the overexpression of *COX6*. Moreover, Aim11 physically interacted with newly synthesized Atp9 ring. Together, this data indicated a possible role of Aim11 in the Atco complex. Additionally, we show that Aim11 forms a complex with the uncharacterized proteins Iai11, Gep7, and Mtc3, which we named AMIGa (Aim11-Mtc3-Iai11-Gep7 assembly) complex. All four components are necessary for optimal CIV assembly. Finally, we also identified the ATP synthase assembly factor TMEM242 (Carroll et al, 2021) as the human orthologue of Aim11, which was able to compensate for the loss of Aim11 in yeast.

## Results

### Aim11 is involved in CIV biogenesis

**Figure 1:**
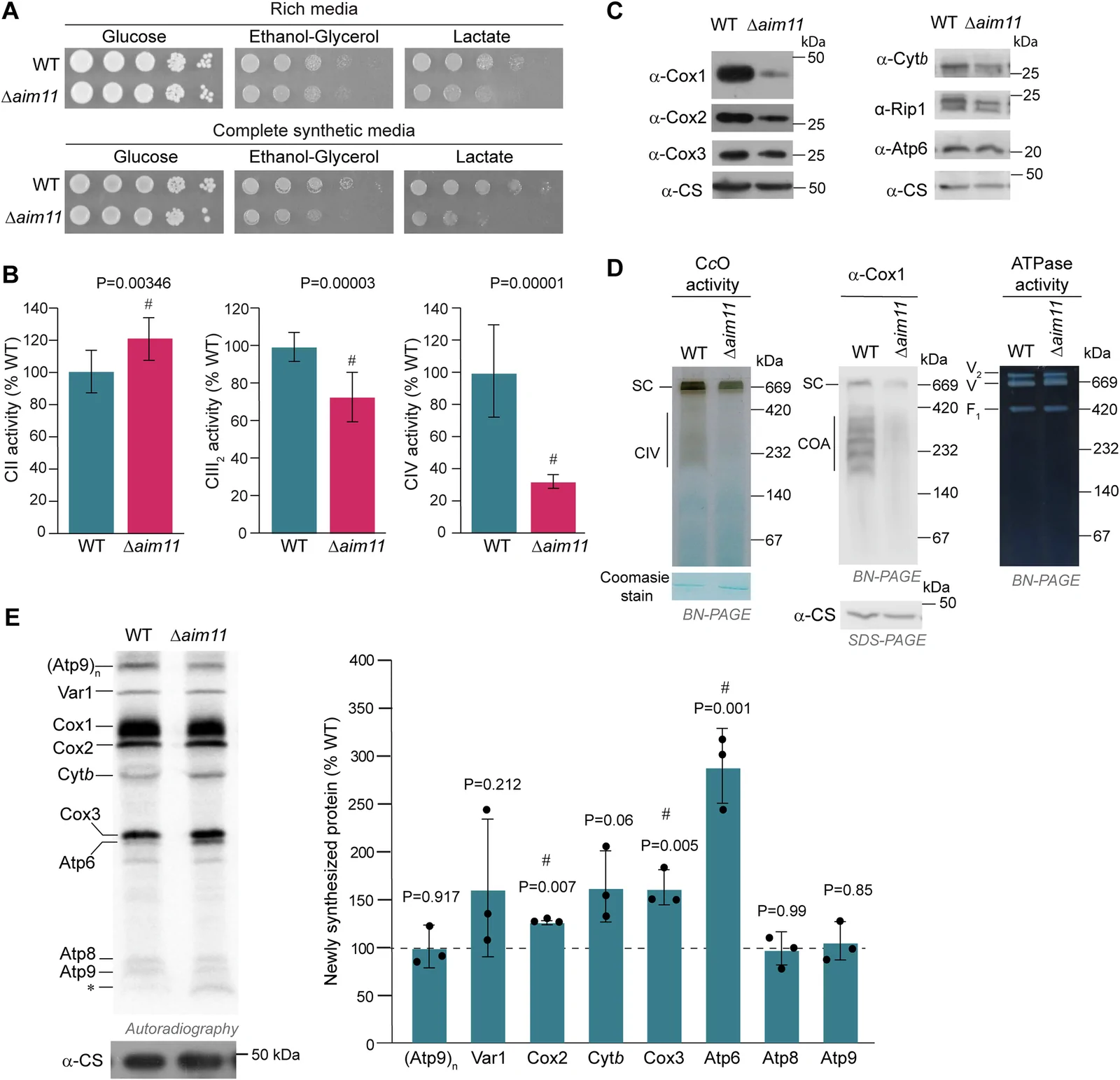
Complex IV biogenesis is decreased in the absence of Aim11. (**A**) Serial dilutions of WT and Δ*aim11* strains in fermentative (glucose) and respiratory (ethanol-glycerol or lactate) media were grown for 2 days (rich media) or 5 days (complete synthetic media) at 30 °C. (**B**) Enzyme activities of CII, CIII_2_, and CIV in isolated mitochondrial membranes (*n* = 9). Specific inhibitor-sensitive activities are represented as a percentage of the WT activity and were corrected to their respective protein content. Statistically significant changes were determined using a student’s *t*-test analysis; **p < 0.01, ***p < 0.001 (*n* = 3 independent preparations; nine technical replicates). (**C**) Western blot of mitochondria (50 µg) from the indicated strains. Citrate synthase was used as a loading control. (**D**) Mitochondria (100 µg) were solubilized with digitonin and separated by BN-PAGE. Part of the gel was transferred to a PVDF membrane for western blot analysis, and the rest was used for in-gel staining activity of CIV and ATP synthase. (**E**) Whole-cell [^35^S]-methionine labeling of mitochondrial-encoded proteins. Cox2, Cox3, Cyt*b*, and Atp6 signals were normalized to Cox1 signal and expressed as a percentage of wildtype. Statistical analysis from three independent biological replicates was performed by multiple unpaired *t*-test analysis to compare each newly synthesized protein to the control (WT). # Significant difference (*p* < 0.05). * Unknown translation product present in the Δ*aim11* mutant. Error bars represent standard deviation.

By high-throughput assays, it was previously observed that Aim11 copurified with some CIV subunits (Morgenstern et al, 2017, Data ref: Morgenstern et al, 2017); however, the physiological relevance of these interactions remained unexplained. To explore the role of Aim11 in CIV biogenesis, we characterized a *Δaim11* mutant. No change in the rho^-^/rho^0^ incidence was detected after deletion of *AIM11*. First, we performed serial dilutions of the wildtype strain and the Δ*aim11* mutant on fermentative (glucose) and respiratory (ethanol/glycerol or lactate) media. While fermentative growth was unaffected, the growth of the Δ*aim11* mutant decreased in ethanol/glycerol and lactate compared to the WT strain (Fig. 1A). The defect was more pronounced on lactate than on ethanol/glycerol media. Unlike ethanol or glycerol, lactate donates its electrons directly to cytochrome *c* (Lodi and Ferrero, 1993), which in turn donates them to CIV, suggesting that a target of Aim11 was CIV.

Next, we evaluated the activity of the respiratory complexes in isolated mitochondria. The most affected complex was CIV, whose activity was reduced to 30% of the WT activity (Fig. 1B). Complex III_2_ (CIII_2_) activity was 73% of the WT strain, while complex II (CII) activity was slightly higher in the Δ*aim11* mutant (120% of the WT strain). Therefore, the respiratory growth defect in the Δ*aim11* mutant was due to a major reduction in CIV activity and, to some extent, also to a reduced CIII_2_ activity.

Next, we asked if the reduction in CIV activity is due to a defect in its assembly. First, we analyzed the steady state levels of CIV subunits (Cox1-3), CIII_2_ (Cyt*b*, and Rip1), and ATP synthase (Atp6) in WT and Δ*aim11* mitochondria (Fig. 1C). Cox1 levels decreased in the mutant, as well as Cox2, Cyt*b* and Rip1. Cox3 and Atp6 subunit levels showed no significant difference between the WT and Δ*aim11* strains. So far, the primary defect resulting from the absence of Aim11 has been observed in CIV activity and in the levels of Cox1 and Cox2. We wondered if the absence of Aim11 could modify the distribution of CIV between the monomeric enzyme and supercomplexes. We performed blue native electrophoresis (BN-PAGE) of digitonin-solubilized mitochondria and analyzed the CIV content by in-gel activity staining and western blot. Both immunodetection of Cox1 and in-gel CIV activity staining revealed that not only Cox1 and Cox2 levels diminished, but there are also lower levels of monomeric CIV and respiratory supercomplexes in the Δ*aim11* strain (Fig. 1D). In contrast, no difference was found on ATP synthase activity staining (Fig. 1D right panel), suggesting that ATP synthase does not seem to contribute to the lower respiratory growth observed in Fig. 1A. Our results indicate that the reduced activity of CIV in the Δ*aim11* mutant was due to lower assembly rather than to a regulatory effect on CIV activity.

Lower levels of mitochondria encoded CIV subunits in the Δ*aim11* mutant (Fig. 1C) could reflect a reduced stability or diminished synthesis of the proteins. To answer this, we tested mitochondrial de novo protein synthesis by incubating yeast cells with [^35^S]-methionine and cycloheximide to inhibit cytosolic translation. Mitochondria were isolated, separated by SDS-PAGE, and analyzed by autoradiography. While Cox1, Cyt*b* and Var1 synthesis were unaffected, synthesis of Cox2 and Cox3 were slightly increased (Fig. 1E). Surprisingly, de novo synthesis of Atp6 greatly increased when Aim11 was absent (Fig. 1E). However, neither Atp6 steady state levels (Fig. 1C) nor ATP synthase levels (Fig. 1D) reflected this increase in translation. Additionally, we consistently observed the presence of an unknown low molecular mass polypeptide that was detected in Δ*aim11* cells but not in WT cells (Fig. 1E). This peptide might originate from Atp9 (see Discussion).

Taken together, these results indicate that Aim11 is required for optimal CIV assembly, while synthesis of the mtDNA-encoded proteins Cox2, Cox3, and Atp6 increased to different degrees. Moreover, the stability of Cox1 was compromised after *AIM11* deletion.

### Aim11 affects the three assembly modules of CIV and stabilizes Cox6

To gain insights into the possible role of Aim11 on CIV assembly, we carried out a complexome profiling analysis of WT and Δ*aim11* mitochondria. This mass spectrometry-based technique helps identify the components of both mature mitochondrial protein complexes and their stable subassemblies (Cabrera-Orefice et al, 2022; Heide et al, 2012). Digitonin-solubilized mitochondria were separated by BN-PAGE. Then, each gel lane was cut into 48–60 slices and subjected individually to trypsin digestion. Identification of the peptides present in each fraction was done by LC-MS/MS. The resulting data provided a global understanding of the composition and migration of the mitochondrial proteins in the native gel and revealed the effects of the Δ*aim11* mutation.

**Figure 2:**
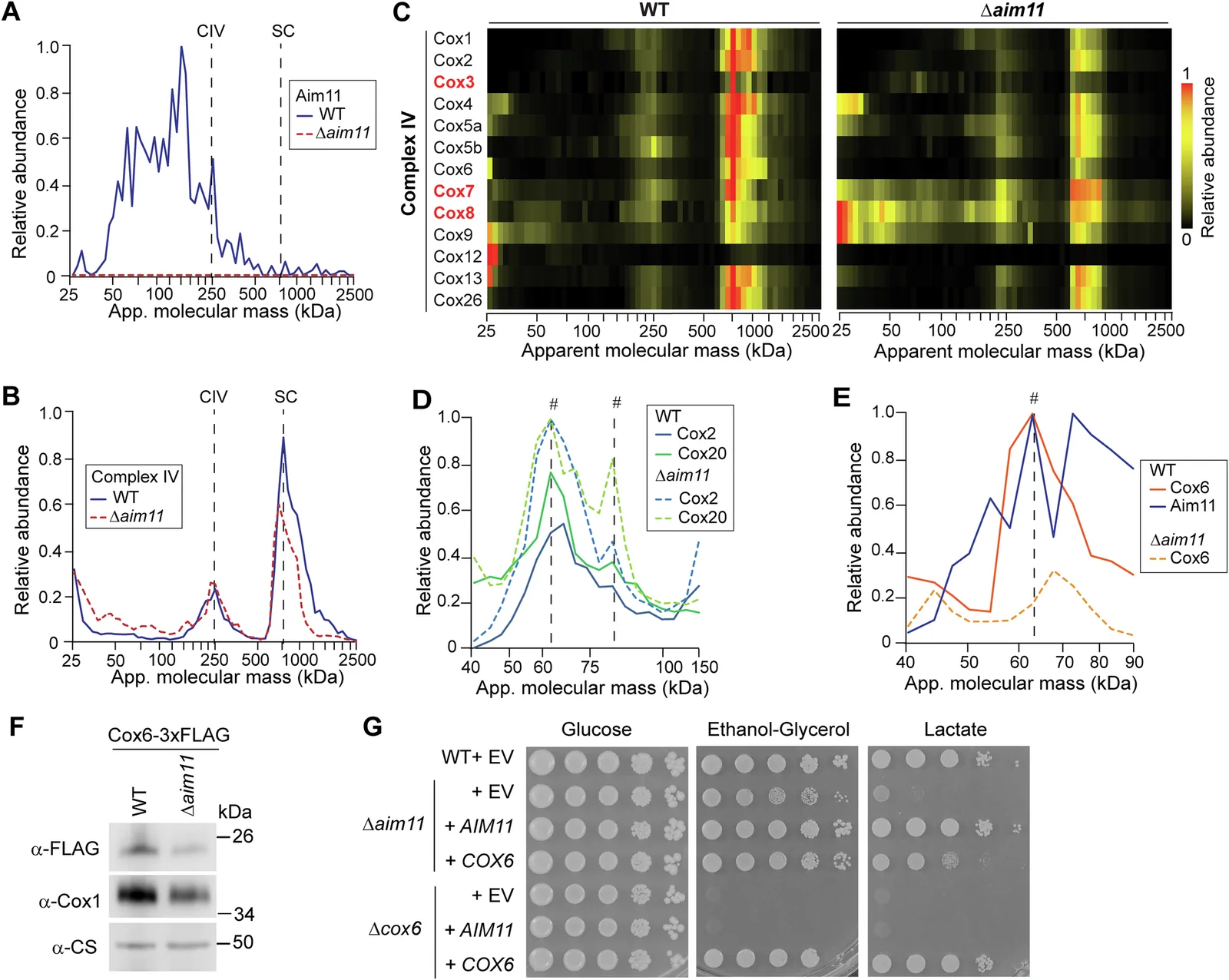
Aim11 seems to regulate Cox2 module assembly as well as subunit Cox6. (**A**) Complexome profiling of Aim11 in mitochondria. (**B**) CIV complexome profiling in WT and *Δaim11* mitochondria. The graphic shows the average of the relative abundance of each subunit forming CIV. (**C**) Heat maps showing the migration patterns of the individual CIV subunits in WT and *Δaim11* mitochondria. Subunits with an altered migration pattern are indicated in red characters. (**D**) Complexome profiles from Cox2 and Cox20 subassemblies showing the relative abundance of WT and *Δaim11* mitochondria. The discontinued vertical lines indicate where the proteins comigrate: # =  ~68 and ~ 90 kDa intermediates. (**E**) Relative abundance profiles of Cox6 and Aim11 from WT and *Δaim11* mitochondria. The dotted lines indicate where the two proteins comigrate: # = ~60 kDa. The corresponding graphs showing the averaged iBAQ values (*n* = 2) instead of the relative abundances are shown in Fig. EV2. (**F**) Steady state levels of Cox6 in the *Δaim11* strain. Western blot against the indicated antibodies. Citrate synthase (CS) was used as a loading control. (**G**) The *COX6* gene was cloned into the YEp352 2 μ plasmid to overexpress it. Serial dilutions of the indicated strains transformed with either an empty vector (EV) or plasmids overexpressing *AIM11* or *COX6*. Cells were grown in CSM media lacking uracil with either fermentative (glucose) or respiratory media (ethanol-glycerol or lactate). Growth shown after 7 days at 30 °C.

**Figure EV1:**
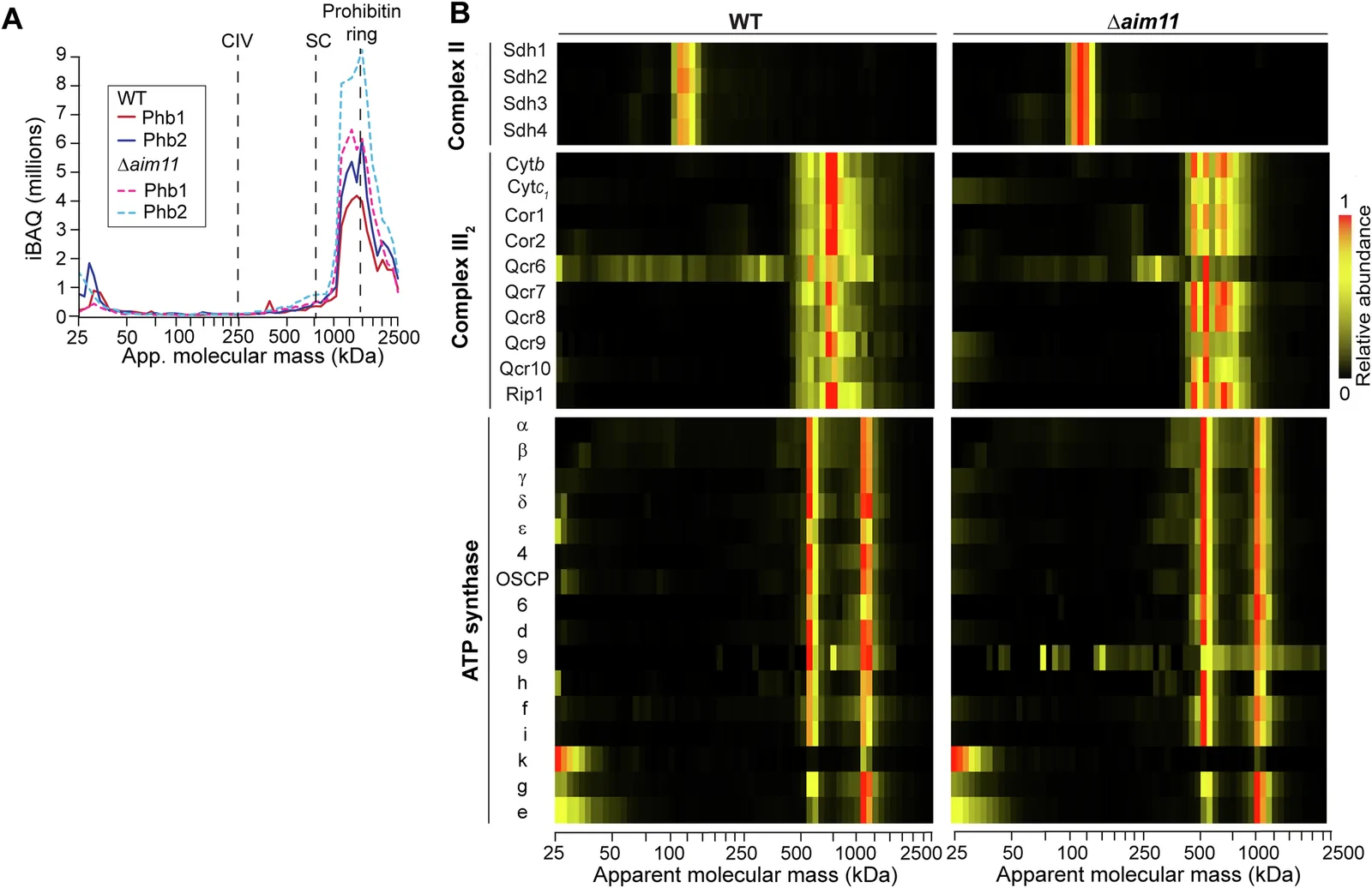
Absence of Aim11 accumulates prohibitin levels, but does not alter CII, CIII_2_, or ATP synthase levels. (**A**) Prohibitin ring (Phb1 and Phb2) complexome migration in WT and *AIM11* null mutant mitochondria. (**B**) Heat maps of the complexome profiling analysis showing the migration patterns of CII, CIII_2_, and ATP synthase individual subunits.

In the WT strain, Aim11 comigrated with various-sized complexes (ranging from ~50 to 400 kDa). Its highest abundance was found at ~150 kDa (Fig. 2A). Since the simultaneous absence of Aim11 and the prohibitin ring are lethal (Osman et al, 2007), we evaluated if Phb1 and Phb2 were affected in the Δ*aim11* mutant. The prohibitin ring migrated at around 1500 kDa, and its relative abundance increased in the null mutant compared to the WT strain (Fig. EV1A).

To establish a direct role of Aim11 in CIV assembly, we analyzed the abundance and migration patterns of the OXPHOS complexes in the WT and the Δ*aim11* strains (Figs. 2B and EV1B). The migration patterns of most CII, CIII_2_, and ATP synthase subunits showed no relevant modification upon *AIM11* deletion (Fig. EV1B). In the mutant, the relative abundance of CIII_2_ subunits was slightly reduced, while the relative abundance of CII subunits showed a minor increase. In contrast, several CIV subunits exhibited changes in their migration patterns and relative abundance in the absence of Aim11 (Fig. 2B). Overall, the average signal of CIV subunits in supercomplexes (~719–950 kDa) decreased in the mutant, whereas no major change in the monomeric fraction (~250 kDa) was observed. Additionally, there was an accumulation of non-assembled subunits (Fig. 2B). Subunit Cox12 was barely detected in CIV and supercomplexes, probably because the interaction of Cox12 with CIV seems to be destabilized under BN-PAGE conditions (Strecker et al, 2016).

Specific subunits showed an altered migration pattern in the Δ*aim11* mutant. Subunits Cox3, Cox7 and Cox8 accumulated in complexes with lower molecular masses (ranging from ~50 to 150 kDa) (Fig. 2C). This enriched low mass population indicated the accumulation of assembly intermediates.

**Figure EV2:**
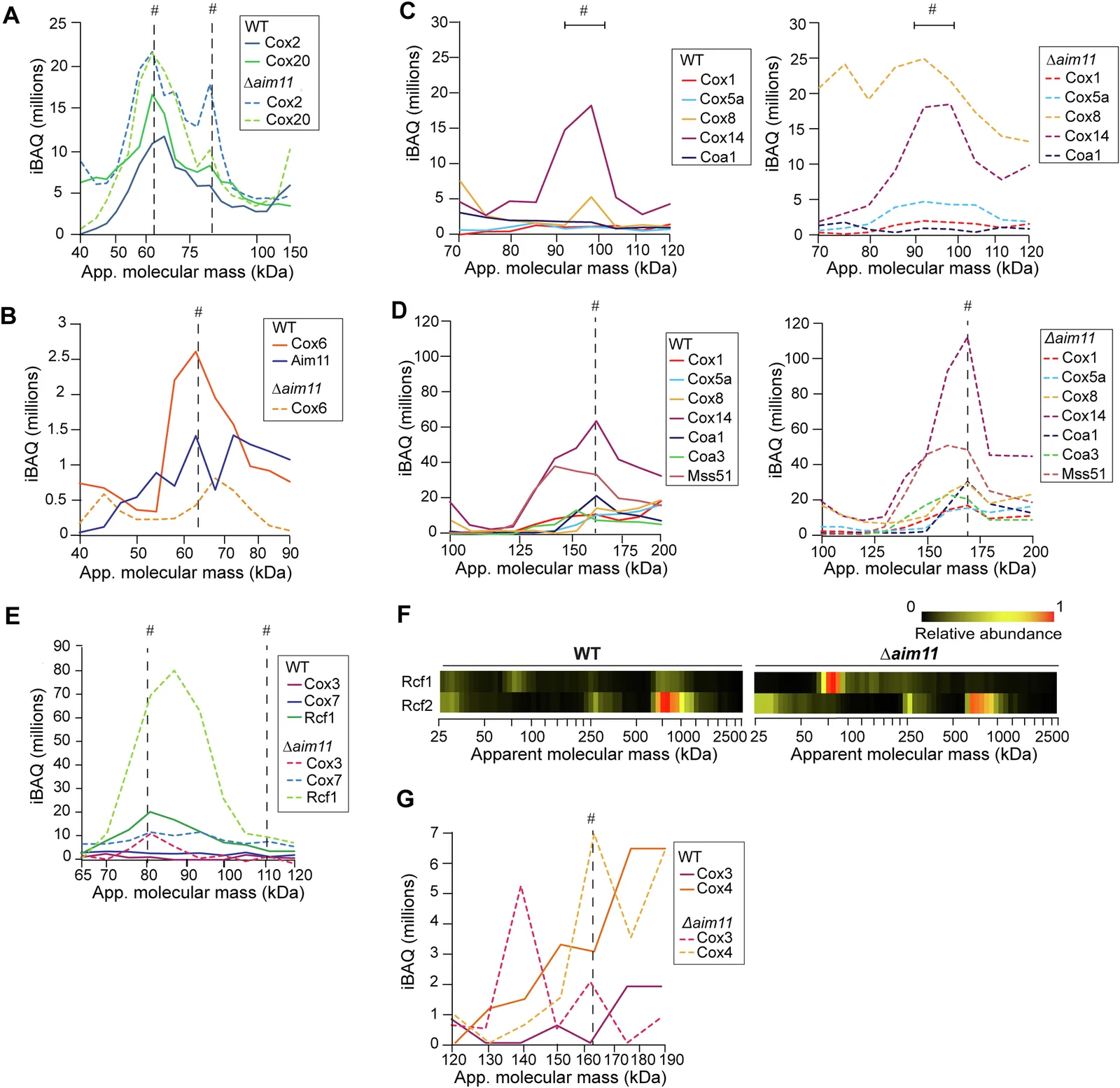
The absence of Aim11 alters the three CIV assembly modules. (**A**–**E**) Abundance and migration patterns of different CIV subunits and assembly factors. The y-axis shows average iBAQ values (*n* = 2) from each protein. (**F**) Heat maps of the complexome profiling analysis showing the migration patterns of Rcf1 and Rcf2. (**G**) Abundance and migration patterns of CIV subunits Cox3 and Cox4. The y-axis shows average iBAQ values (*n* = 2) from each protein.

Previous work showed that Aim11 copurified with Cox2, Cox20 and Cox6 (Morgenstern et al, 2017, Data ref: Morgenstern et al, 2017), so we evaluated more in-depth the migration pattern of these proteins. Cox20 interacts with unassembled Cox2, and it is involved in Cox2 stabilization, translocation, and processing (Elliott et al, 2012; Lorenzi et al, 2016). We observed the Cox2-Cox20 subcomplex in two peaks (~68 and 90 kDa) in both strains (Figs. 2D, and EV2A), which are like the ones previously identified (Lorenzi et al, 2016). In the Δ*aim11* mutant, the levels of both subassemblies increased. The observed intermediates' apparent molecular mass was higher than the theoretical one (52 kDa), indicating that additional proteins were present. Even though overall Cox2 levels were lower (Fig. 1C), there was an accumulation of the Cox2-Cox20 intermediates, suggesting that Aim11 affects the early biogenesis steps of the Cox2 module.

Cox6, a nuclear encoded subunit that is part of the Cox1 assembly module (McStay et al, 2013b), was also previously reported to interact with Aim11 (Morgenstern et al, 2017, Data ref: Morgenstern et al, 2017). We evaluated whether a fraction of Aim11 could migrate with Cox6. The complexome analysis showed that most of Cox6 comigrated with CIV and supercomplexes, and only a small portion comigrated with Aim11 at a molecular mass of ~60 kDa (Figs. 2E and EV2B). In the Δ*aim11* strain, this peak disappeared, supporting the existence of a Cox6-Aim11 complex (Fig. 2E). This association has a theoretical molecular mass of 33 kDa, indicating a different stoichiometry of Cox6:Aim11 or the presence of additional unidentified proteins. The interaction previously reported (Morgenstern et al, 2017, Data ref: Morgenstern et al, 2017) and the complexome data shown here suggest that the Cox6-Aim11 interaction could help stabilize Cox6 and facilitate its assembly into the Cox1 module. Thus, we evaluated Cox6 levels in the absence of Aim11, and, as expected, Cox6 levels decreased (Fig. 2F). We hypothesized that in the Δ*aim11* mutant, Cox6 may be a limiting factor for CIV assembly. To test this, we overexpressed *COX6* in a Δ*aim11* mutant and monitored the respiratory growth of the resulting cells. Overexpression of *COX6* resulted in a partial recovery of the respiratory growth defect of the Δ*aim11* strain (Fig. 2G). Our data suggests that part of Aim11 role on CIV assembly is related to the assembly of Cox6. However, as the respiratory growth did not fully recover, Aim11 must have additional functions.

**Figure 3:**
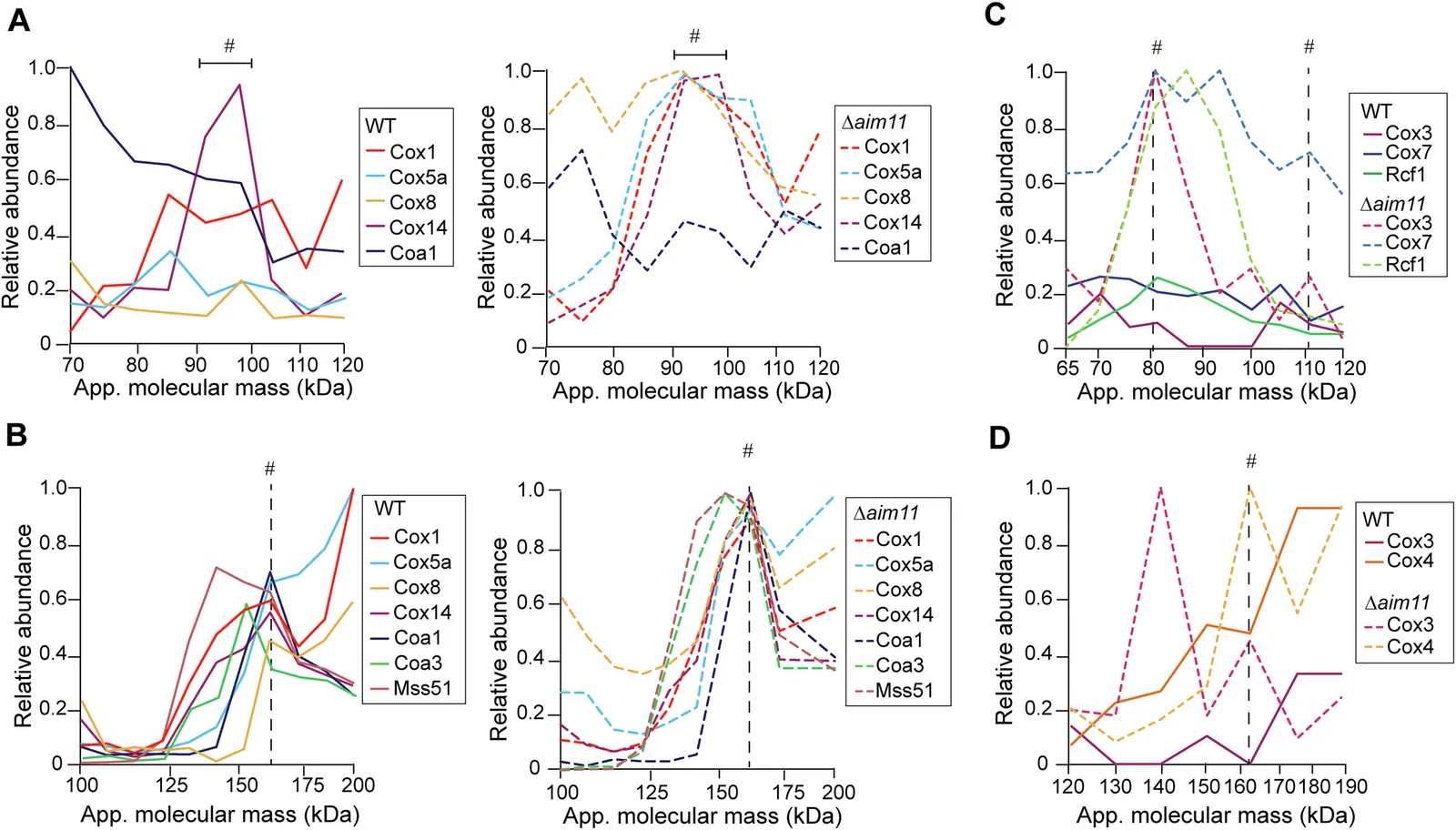
Aim11 also plays a role in the assembly of Cox1 and Cox3 modules. (**A**) Complexome analysis of Cox1 subcomplexes at ~90–100 kDa from WT (left panel) and *Δaim11* (right panel) mitochondria. Comigration of proteins is indicated with the # symbol. The corresponding graph showing average iBAQ values instead of relative values is presented in Fig. EV2C. (**B**) Relative abundance graph of the ~160 kDa Cox1 subassembly from WT (left panel) and *Δaim11*(right panel) mitochondria. The dotted line indicates where the proteins comigrate (#). For the corresponding iBAQ graph, see Fig. EV2D. (**C**) Complexome migration profile of Cox3, Cox7, and Rcf1 from WT and *Δaim11* mitochondria. The dotted lines indicate where the three proteins comigrated: # = ~80 or ~110 kDa intermediate. For the corresponding iBAQ graph, see Fig. EV2E. (**D**) Complexome migration profile of Cox3 and Cox4 from WT and *Δaim11* mitochondria. The dotted line indicates where the proteins comigrate: # =  ~160 kDa. For the corresponding iBAQ graph, see Fig. EV2F.

Next, we looked for the presence of other Cox1 module assembly intermediates that could be affected by the Δ*aim11* mutation. During early assembly, newly translated Cox1 interacts with proteins like Cox14, Coa1, Coa3 and Mss51 (McStay et al, 2013b). Additionally, the subunits Cox4, Cox5a, Cox6 and Cox8 associate with Cox1 during assembly (McStay et al, 2013a). Complexome profiling data of the WT strain showed two Cox1-containing subassemblies (Fig. 3A, B). The first one had a molecular mass ranging from ~90 to 100 kDa, and it consisted of subunits Cox1, Cox5a, and Cox8 plus the chaperones Coa1 and Cox14 (Figs. 3A and EV2C). The theoretical molecular mass of this complex is 113 kDa, which correlates well with the observed migration. This peak was sharper in the Δ*aim11* strain, where Cox1, Cox5a, Cox8, and Cox14 levels increased, suggesting an accumulation of this subcomplex. The second Cox1 subassembly (~160 kDa) contained the same components as the first subcomplex (Cox1, Cox5a, Cox8, Cox14, Coa1) plus Coa3 and Mss51 (Figs. 3B and EV2D). In the Δ*aim11* mutant, there was an accumulation of this subcomplex. The theoretical molecular mass of this subcomplex is 165 kDa, which also correlates well with the observed molecular mass. The accumulation of the ~90–100 kDa and the ~160 kDa subassemblies in the Δ*aim11* mutant suggests that Aim11 assists the progression of the Cox1 module assembly.

As previously stated, Cox3 also showed several new or enriched peaks in the Δ*aim11* mutant (Fig. 2C), suggesting that the Cox3 assembly module is also affected. Subunits Cox3, Cox4, Cox7, Cox13, and the assembly factor Rcf1, form the Cox3 module (Su et al, 2014a). While analyzing their migration patterns in the complexome dataset (Fig. 3C,D), we detected a subcomplex with an apparent molecular mass of ~80 kDa containing Cox3, Cox7, and Rcf1, however it was not clearly defined (Figs. 3C and EV2E). In contrast, this subcomplex was greatly enriched and defined in the Δ*aim11* mutant. The theoretical molecular mass of the Cox3-Cox7-Rcf1 complex is 55 kDa, suggesting the presence of additional unidentified proteins. In addition to the ~80 kDa subcomplex, Rcf1 migrated with the monomeric CIV (~254 kDa) and with supercomplexes (~765 kDa) (Fig. EV2F). Meanwhile, in the Δ*aim11* strain, the Rcf1 peak corresponding to the supercomplexes dramatically decreased, while the Rcf1 peak comigrating with the monomeric CIV showed a milder decrease (Fig. EV2F). Rcf1 has a homolog, Rcf2, which is believed to be involved in super complex formation but not in Cox3 assembly (Strogolova et al, 2012). Unlike Rcf1, the Rcf2 migration pattern in the WT and Δ*aim11* strains did not change nor seemed to associate into the ~80 kDa subcomplex (Fig. EV2F), suggesting that the Rcf2 role is independent of Aim11.

We observed two additional intermediates containing Cox3 that accumulated after *AIM11* deletion. One of them, containing Cox3 and Cox7, was detected at ~110 kDa (Figs. 3D and EV2G). The second one was detected at ~160 kDa and consisted of Cox3 associated with Cox4 (Figs. 3D and EV2G). The theoretical molecular masses of both subcomplexes are 37 and 47 kDa, respectively, indicating that there must be other unidentified components in both subassemblies. These results strongly suggest that Aim11 is required for efficient progression of the Cox3 module.

Altogether, our complexome profiling analysis supports a model in which Aim11 has a role in CIV biogenesis under the tested conditions. In its absence, the assembly of CIV is inefficient, resulting in an accumulation of intermediates from all three assembly modules. We also observed that the absence of Aim11 limited the availability of Cox6 for assembly into the Cox1 module and compromised Cox6 stability.

### The AMIGa complex is formed by Aim11, Iai11, Gep7, and Mtc3

Next, we asked whether Aim11 acts alone in CIV biogenesis or as part of a complex. Previous and recent work has shown that Aim11 copurifies with other uncharacterized proteins, including the Aim11 paralogue, Iai11, plus Mtc3 and Gep7 (Morgenstern et al, 2017; Data ref: Morgenstern et al, 2017; preprint: Kizmaz et al, 2026). Like Aim11, Iai11 is an inner membrane protein with two transmembrane helices, with both N- and C-termini exposed to the intermembrane space (Morgenstern et al, 2017). Both Aim11 and Iai11 copurified with the same set of CIV and ATP synthase subunits in high-throughput experiments (Morgenstern et al, 2017, Data ref: Morgenstern et al, 2017). Mtc3 and Gep7 are membrane-integral mitochondrial proteins (Huh et al, 2003) with a predicted mitochondrial targeting sequence and one transmembrane helix. Like *AIM11*, the *GEP7* gene shows a negative genetic interaction with genes encoding prohibitins (Osman et al, 2009). The *Δmtc3* mutant is synthetically sick when found in combination with the *cdc13-2* allele, encoding a mutant version of Cdc13, a telomeric single-stranded DNA binding protein (Addinall et al, 2008). Other than these facts, the functions of Iai11, Gep7, and Mtc3 remain unknown.

**Figure 4:**
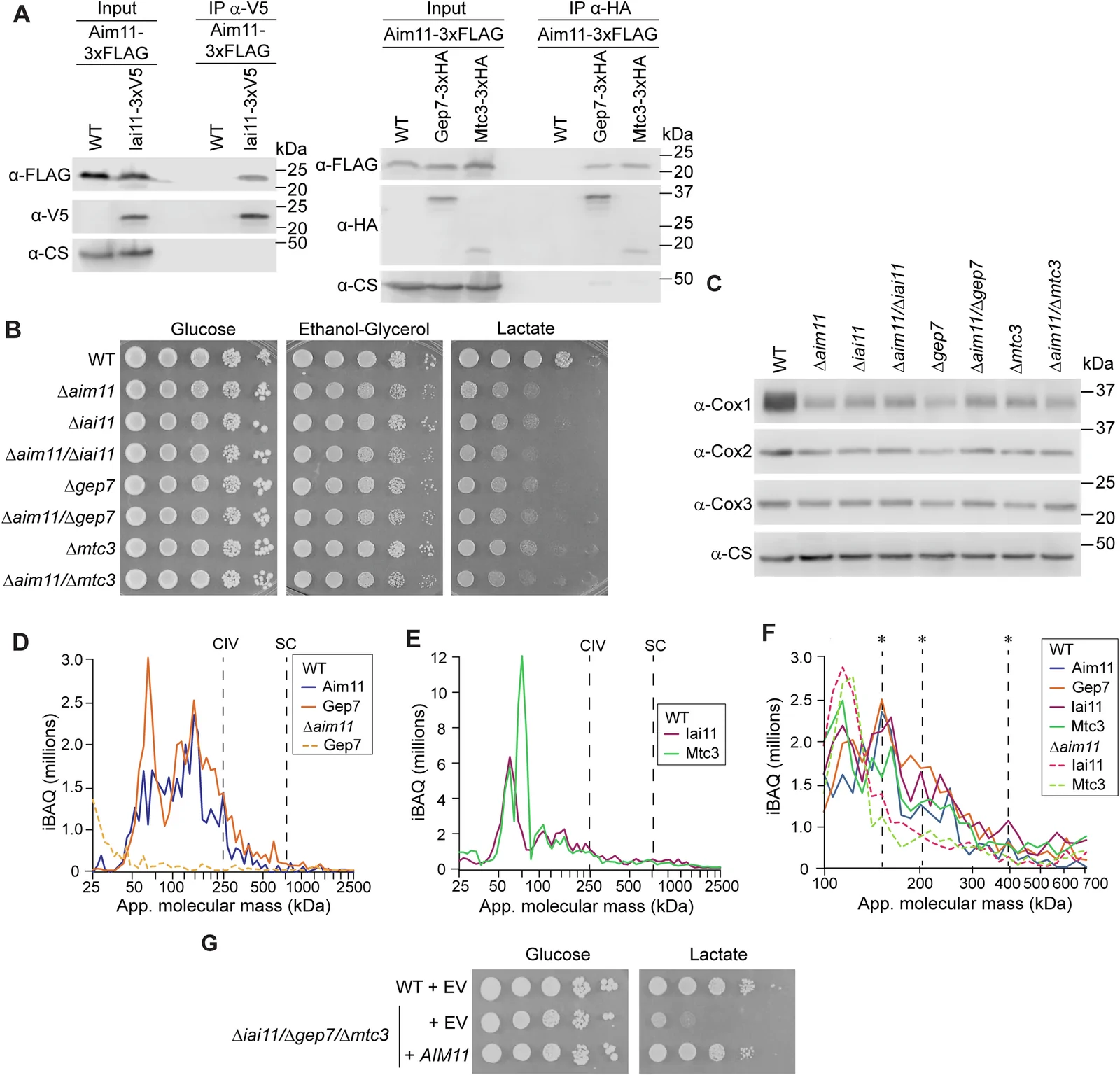
Aim11 forms a complex with Iai11, Gep7, and Mtc3 (AMIGa complex) that participates in complex IV biogenesis. (**A**) Mitochondria were isolated, and tagged versions of Iai11, Gep7 or Mtc3 were immunoprecipitated with the indicated antibodies. Input = 10% of total pulldown protein. Citrate synthase (CS) was used as a non-interacting control. (**B**) Serial dilutions of the indicated strains in fermentative (glucose) and respiratory media (ethanol-glycerol or lactate). Growth in CSM media was shown after 3 days at 30 °C. (**C**) Western blot of mitochondria (20 µg of protein per lane) showing steady state levels of the indicated proteins. Citrate synthase (CS) was used as a loading control. (**D**) Gep7 and Aim11 complexome migration patterns in a WT and *Δaim11* mutant. Data are shown as iBAQ values. (**E**) Mtc3 and Iai11 complexome profiling in a WT strain. Data were shown as iBAQ values. (**F**) Aim11, Iai11, Mtc3, and Gep7 complexome migration patterns in a WT strain and a *Δaim11* mutant. Peaks where all four proteins comigrate are indicated with an asterisk (*). Data were shown as iBAQ values. (**G**) Serial dilutions of the WT strain and *Δiai11/Δgep7/Δmtc3* triple mutant with the indicated plasmid in fermentative (glucose) or respiratory media (lactate) lacking uracil. EV: empty vector.

Therefore, we first decided to confirm the existence of the Aim11-Iai11-Mtc3-Gep7 complex proposed by Morgenstern (2017). We immunoprecipitated tagged versions of Iai11, Mtc3 or Gep7 and in all cases Aim11 copurified with them (Fig. 4A). We thus coined the cluster of these four proteins as the Aim11-Mtc3-Iai11-Gep7 assembly complex or AMIGa complex.

**Figure EV3:**
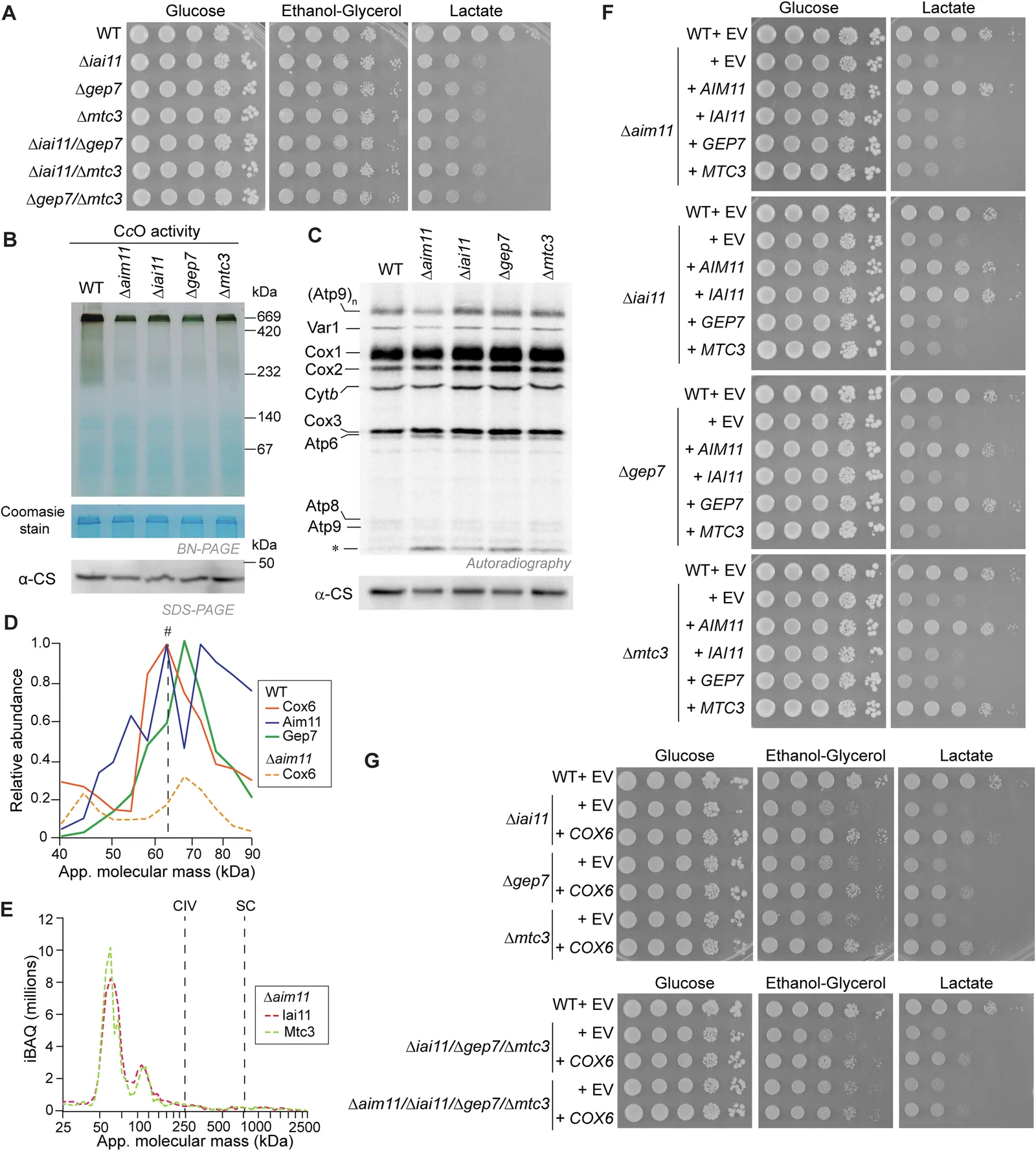
Aim11 is the core component of the AMIGa complex. (**A**) Serial dilutions of the indicated strains in fermentative (glucose) and respiratory media (ethanol-glycerol or lactate). Growth in CSM media was shown after 3 days at 30 °C. (**B**) Mitochondria (100 µg) were solubilized with digitonin and separated by BN-PAGE. In-gel staining activity of CIV was performed. A fraction of the sample was separated by SDS-PAGE and transferred to a PVDF membrane for western blot and probed with an antibody against citrate synthase and was used as loading control. (**C**) Mitochondrial translation products from whole cells were labeled with [^35^S]-methionine in the indicated strains. An unknown translation product was present in all the null mutants (*). (**D**) Complexome profiles of Gep7, Aim11, and Cox6. A comigration signal at around 60 kDa is indicated with the # symbol. This comigration disappeared in the absence of Aim11. (**E**) Complexome profile showing migration patterns of Iai11 and Mtc3 in the absence of Aim11. The apparent molecular mass of Complex IV (CIV) and supercomplexes (SC) is indicated. (**F**) Serial dilutions of the null mutants of AMIGa complex components with the indicated overexpression plasmids. Cells were grown in either fermentative (glucose) or respiratory (lactate) media lacking uracil. Growth shown after 5 days at 30 °C. EV is empty vector. (**G**) Serial dilutions of the single, triple and quadruple *AIM11, IAI11, GEP7*, and* MTC3* null mutant strains with the indicated overexpression plasmids. Cells were grown in either fermentative (glucose) or respiratory (ethanol-glycerol or lactate) media lacking uracil for 5 days at 30 °C. EV is empty vector.

After this validation, we next asked if these proteins are also functionally related and whether Iai11, Gep7, and Mtc3 are involved in CIV biogenesis. To tackle this, we analyzed the growth phenotype of *Δiai11*, *Δgep7*, and *Δmtc3* single mutants. The corresponding strains showed decreased respiratory growth as compared to the WT cells (Fig. 4B). However, these mutants grew slightly better compared to the Δ*aim11* single mutant. We also evaluated the respiratory growth of double mutants in search of possible genetic interactions between them and *AIM11*. The double mutants *Δaim11/Δiai11*, *Δaim11/Δgep7*, and *Δaim11/Δmtc3* showed a similar respiratory growth defect as the single mutants (Fig. 4B). Similarly, no additional respiratory growth defect was observed in the double mutants *Δiai11/Δgep7*, *Δiai11/Δmtc3* and *Δgep7/Δmtc3* (Fig. EV3A), indicating that no genetic interaction exists among the AMIGa complex components. As with Aim11, all these results suggest that Iai11, Gep7, and Mtc3 are necessary but not essential for optimal respiratory growth. However, the absence of Aim11 leads to a more severe respiratory defect than the individual deletion of the other AMIGa complex components.

Next, we evaluated steady state levels of mitochondrial CIV subunits in mutants of the AMIGa complex components (Fig. 4C). Just like the Δ*aim11* mutant, the absence of Gep7, Iai11, and Mtc3 showed lower levels of CIV subunits. Once again, Cox1 was the most affected subunit, although Cox2 showed a lower accumulation. The Cox3 levels were maintained, as observed for the Δ*aim11* mutation (Fig. 1C). In-gel cytochrome *c* oxidase activity staining was also similarly decreased in all the mutants (Fig. EV3B). In mitochondrial translation assays, as observed for the Δ*aim11* strain, in *Δiai11*, *Δgep7*, and *Δmtc3* mutants, the [^35^S]-methionine labeling was not significantly affected, except for Atp6 synthesis, which was increased compared to the WT strain (Fig. EV3C). The same unknown low molecular mass peptide that we detected in the Δ*aim11* mitochondria was observed in all the mutants (see also Fig. 1E). Together, these results suggest that Aim11, Gep7, Mtc3, and Iai11 participate in the same assembly pathway of CIV.

To gain a deeper understanding of the AMIGa complex, we analyzed and compared the migration patterns of each component in the complexome dataset. On the one hand, Gep7 showed a very similar migration pattern to Aim11 (Fig. 4D) but was mostly undetectable in the Δ*aim11* strain (Fig. 4D). This suggests that Gep7 stability depends on Aim11 and that both proteins directly interact. A portion of Gep7 comigrated with the Aim11-Cox6 subcomplex (Fig. EV3D). The theoretical mass of the Aim11-Gep7-Cox6 complex (~60 kDa) matched the observed molecular mass, which supports the idea that Aim11 and Gep7 form a heterodimer. On the other hand, Iai11 and Mtc3 presented a similar migration pattern, suggesting that both proteins form a second heterodimer (Fig. 4E). Whereas Gep7 levels were compromised in the Δ*aim11* strain, Iai11 and Mtc3 levels were not significantly affected (Fig. EV3E). The sum of the molecular mass of one copy of each protein is ~87 kDa, so we searched for a possible comigration around this range. We found three peaks (~150, 200, and 400 kDa) where all four proteins were present (Fig. 4F). In these regions, when Aim11 (and therefore Gep7) were absent, Iai11 and Mtc3 levels were greatly reduced (Fig. 4F), co-validating the existence of the AMIGa complex. Similar migration patterns between Aim11 and Gep7, and between Iai11 and Mtc3, were also observed in a previously reported complexome dataset of yeast WT mitochondria (Schulte et al, 2023). Additionally, all four proteins comigrated at around 147 kDa, consistent with the 150 kDa peak where we found the AMIGa complex.

All four mutants share similar phenotypes, which suggest that all components of the AMIGa complex are necessary for its function. We tested if the overexpression of one component of the complex could compensate for the absence of another. Surprisingly, the overexpression of *AIM11* compensated for the respiratory growth defect of the single *Δgep7, Δiai11*, and *Δmtc3* mutants as well as the triple *Δgep7/Δiai11/Δmtc3* mutant (Figs. 4G and EV3F). In contrast, the overexpression of *GEP7*, *IAI11*, or *MTC3* did not compensate for the absence of Aim11 (Fig. EV3F). This result suggests that Aim11 is the central component of the AMIGa complex, while Gep7, Iai11, and Mtc3 play ancillary roles in CIV biogenesis. Moreover, the growth phenotype indicated that these proteins share common functions, although they do not seem to have overlapping roles. Even if Gep7 depended on Aim11 to form the heterodimer (Fig. 4D), *AIM11* overexpression could compensate for the *Δgep7* strain's respiratory growth, suggesting that Aim11 might act independently of the hypothetical Aim11-Gep7 heterodimer or that potential Aim11 homodimers may also be functional. Finally, to reinforce the idea that Gep7, Iai11, and Mtc3 also have a common role in CIV assembly, we tested if overexpression of *COX6* could also compensate for the respiratory capacity of mutant cells lacking each one of these proteins. As observed for the Δ*aim11* mutant, overexpression of *COX6* partially recovered the respiratory phenotype of single *Δgep7, Δiai11 or Δmtc3* mutants, of the triple *Δgep7/Δiai11/Δmtc3* and of the quadruple *Δaim11/Δgep7/Δiai11/Δmtc3* mutants (Fig. EV3G). This observation reinforces the idea that the AMIGa complex, and not only Aim11, participates in the efficient assembly of CIV.

### TMEM242 is the Aim11 human orthologue

We asked whether the AMIGa complex is conserved. By primary structure analysis (BLAST), we found that Aim11, Iai11, Gep7, and Mtc3 have orthologues only among fungi but not among metazoans. We then performed a secondary structure analysis of these proteins using the HHPRED platform (Zimmermann et al, 2018) to search for possible structural orthologues in humans, *Drosophila melanogaster*, and *Caenorhabditis elegans*. Surprisingly, Gep7 and Mtc3 showed a similar secondary structure to human, fly, and worm COX4 subunit (which is equivalent to the yeast Cox5a/b orthologue) with a probability of >99%, and an E-value <2.5e^-17^. The similarity in secondary structure between Cox5a/b and Gep7, as well as Mtc3, could facilitate the interaction of Gep7 and Mtc3 with Cox6, or with other CIV subunits during assembly.

**Figure 5:**
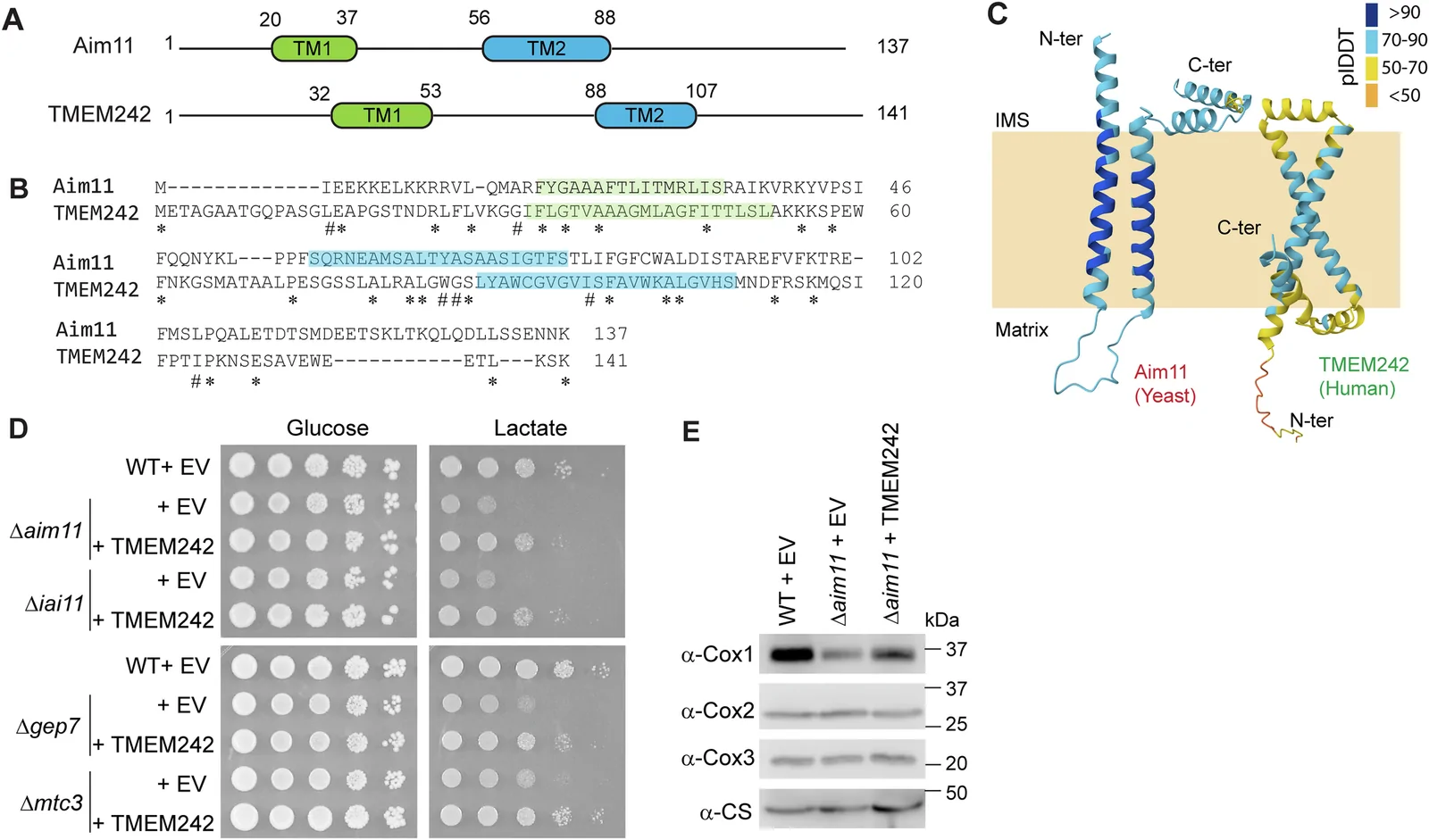
TMEM242 is the human orthologue of Aim11. (**A**) Secondary structure comparison between Aim11 and TMEM242. (**B**) Alignment by CLUSTAL0 of Aim11 and its human orthologue TMEM242. Identical (*) or similar (#) amino acid residues are indicated. Predicted transmembrane domains are shown in blue or green boxes. (**C**) Alphafold3 (Abramson et al, 2024) prediction models of human TMEM242 and yeast Aim11. The experimentally determined topology is illustrated. (**D**) Serial dilutions of the indicated yeast strains with overexpression of TMEM242. Growth in respiratory (lactate) and fermentative media (glucose) lacking uracil for 5 days at 30 °C. (**E**) Immunodetection of mitochondrial proteins from yeast cells where TMEM242 was overexpressed. Citrate synthase (CS) was used as a loading control. EV empty vector.

Next, we detected that the secondary structures of Aim11 and Iai11 were like the TMEM242 protein in humans, flies, and worms (probability >99%, E-value <1.9e^−23^). TMEM242 is an inner mitochondrial membrane protein implicated in complex I and ATP synthase assembly (Carroll et al, 2021). Secondary structure predictions of Aim11 and TMEM242 revealed that both proteins possess two transmembrane domains (Fig. 5A). Alignment of the Aim11 and TMEM242 amino acid residues indicated a low identity between them (21.14%) (Fig. 5B). However, experimental data suggested that Aim11 and Iai11 N- and C- termini are exposed to the intermembrane space (Morgenstern et al, 2017), while exposed to the matrix in the case of TMEM242 (Carroll et al, 2021) (Fig. 5C).

**Figure EV4:**
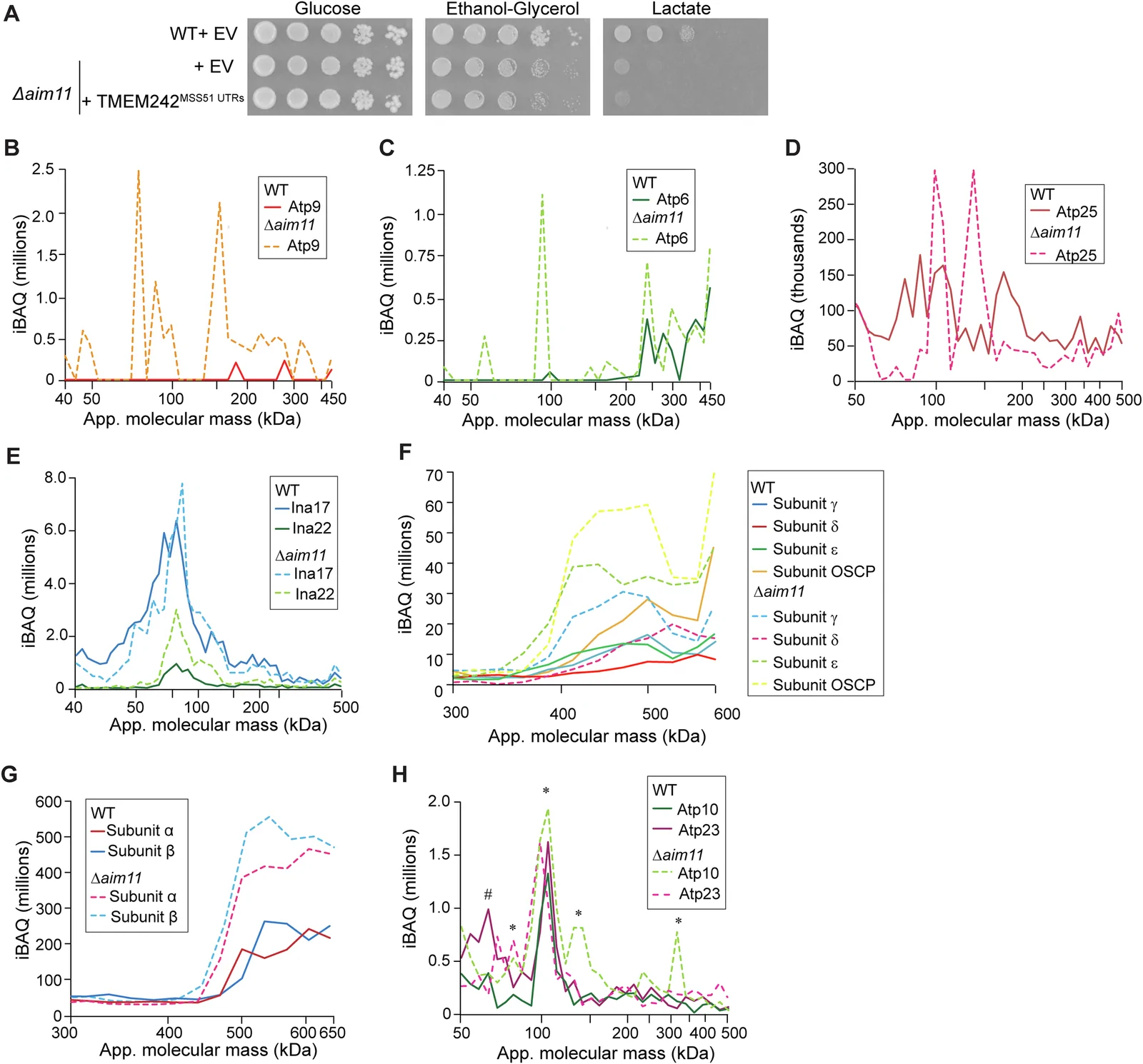
TMEM242 rescues the respiratory phenotype of a *Δaim11* mutation only when it is overexpressed. (**A**) Serial dilutions of *Δaim11* cells with the indicated plasmids. *TMEM242* was cloned on a centromeric plasmid with the promoter and terminator sequences of the *MSS51* gene (*TMEM242*^*MSS51 UTRs*^). Cells were grown in media lacking uracil with glucose, ethanol-glycerol, or lactate as a carbon source. Cells were grown for 5 days at 30 °C. EV is empty vector. (**B**–**H**) Complexome migration patterns of the indicated ATP synthase subunits and assembly factors. The y-axis shows average iBAQ values (*n* = 2) from each protein. In (**H**), peaks only observed in WT (#) or *Δaim11*(*) mitochondria are indicated.

Despite the low sequence identity, we tested whether TMEM242 could compensate for the absence of different components of the AMIGa complex. We cloned the human intronless *TMEM242* from the K562 cell line into a 2 μ yeast expression vector under the *ADH* promoter and terminator. Then, the plasmid was transformed into different yeast mutants. TMEM242 compensated for the respiratory growth in the Δ*aim11*, *Δgep7*, *Δiai11*, and *Δmtc3* mutants (Fig. 5D). We then analyzed the steady state levels of CIV mitochondrial-encoded subunits in WT and Δ*aim11* cells and found that TMEM242 restored Cox1 levels (Fig. 5E). Only overexpressed TMEM242 was able to compensate for the absence of the AMIGa complex (Fig. EV4A). These results strongly indicate that TMEM242 is the human orthologue of yeast Aim11.

### ATP synthase biogenesis is also a target of the AMIGa complex

**Figure 6:**
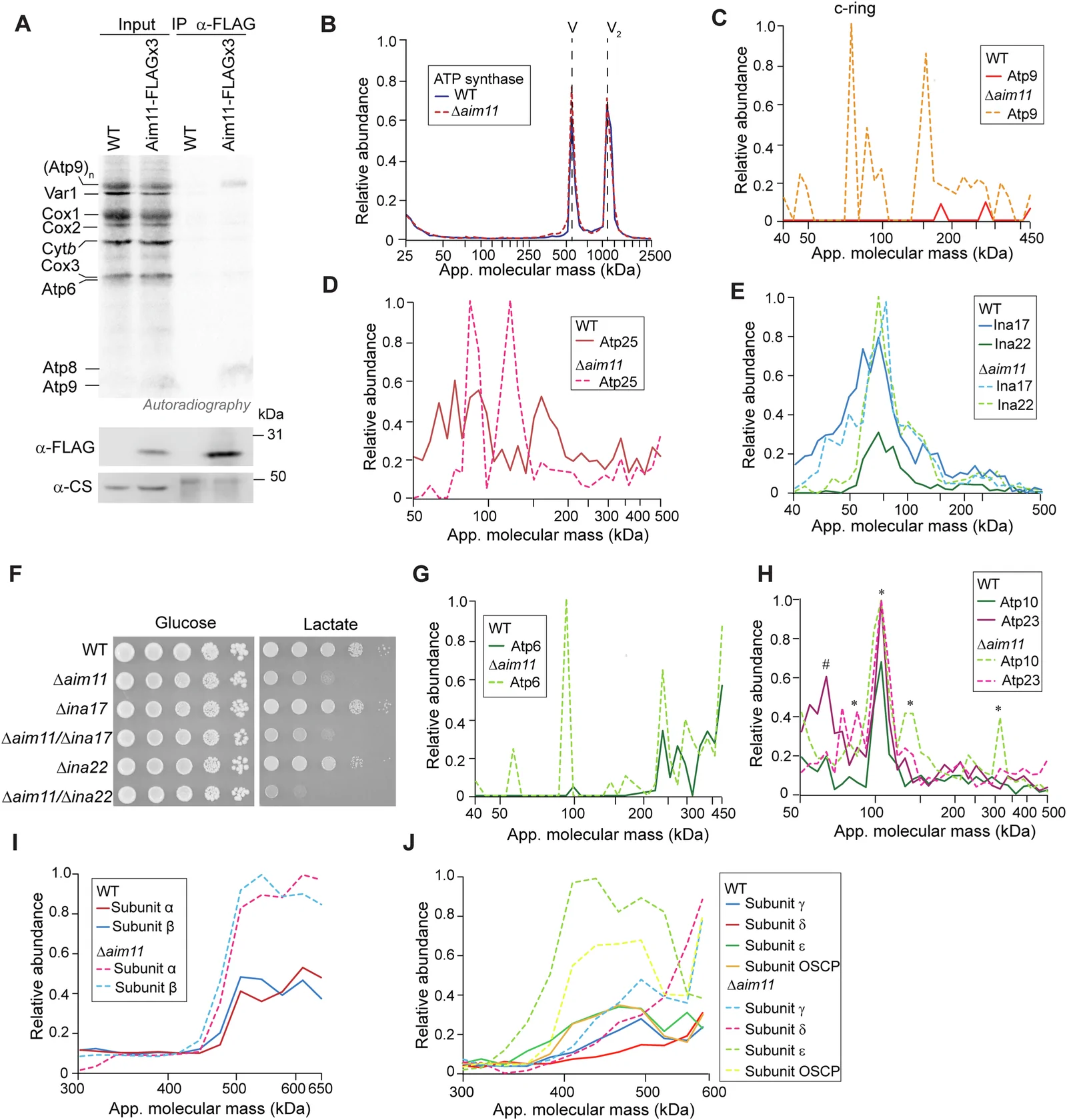
Aim11 has a role in ATP synthase assembly. (**A**) Translation in isolated mitochondria was carried out in the presence of [^35^S]-methionine. After digitonin solubilization, the indicated proteins were immunoprecipitated, and the samples were separated by SDS-PAGE and transferred to a PVDF membrane. The signals were detected by autoradiography and Western blot. Input is 5% of the total sample. (**B**) ATP synthase complexome profiling in WT and *Δaim11* mitochondria. The graphic shows the average of the relative abundance of each subunit conforming ATP synthase. (**C**) Complexome profiles for Atp9 subassemblies showing the relative abundance from WT and *Δaim11* mitochondria. (**D**, **E**) Complexome profiles for Atp25 (**D**) or Ina17 and Ina22 (**E**) showing the relative abundance from WT and *Δaim11* mitochondria. (**F**) Serial dilutions of the indicated strains in fermentative (glucose) and respiratory media (lactate). Growth in CSM media was shown after 5 days at 30 °C. (**G**–**J**). Complexome profiles for the indicated subassemblies showing the relative abundance from WT and *Δaim11* mitochondria. Peaks only observed in WT (#) or *Δaim11*(*) mitochondria.

Since Aim11 orthologue, TMEM242, assists in the assembly of human ATP synthase c_8_-ring (Carroll et al, 2021), we asked if its yeast orthologue Aim11 could also have a role in ATP synthase biogenesis. As observed in mitochondrial translation assays (Fig. 1E), Atp6 subunit synthesis is increased in a Δ*aim11* strain, strongly suggesting that the yeast Aim11 is also involved in ATP synthase biogenesis. We asked whether Aim11 could interact with newly synthesized ATP synthase subunits encoded in the mitochondrial genome (Atp6, Atp8, and Atp9). Thus, we performed a translation assay in isolated mitochondria (in organello), and we coupled this assay with digitonin solubilization and Aim11-3xFLAG immunoprecipitation (Fig. 6A). Like previously reported for TMEM242 (Carroll et al, 2021), Aim11 copurified with the newly synthesized Atp9 c-ring. No other mitochondrial-encoded protein interaction was clearly detected.

In yeast, ATP synthase and CIV assembly are coupled through the Atco complex, a shared intermediate composed of the Atp9 c-ring and the CIV subunit Cox6 (Su et al, 2014b). Cox6 is bound to the Atp9 ring before associating with the Cox1-Cox5-Cox8 intermediate (Franco et al, 2020a). Alterations in the assembly of the c-ring caused a defect in complex IV assembly. Since Aim11 copurified with both components of the Atco complex, the AMIGa complex may be assisting the assembly of both cytochrome *c* oxidase and ATP synthase.

To evaluate this, we closely analyzed the migration patterns of the ATP synthase subunits and chaperones on our complexome dataset. First, we compared the overall levels of the ATP synthase monomer and dimer in the WT and *∆aim11* data (Fig. 6B). There was no clear difference between the two strains, which is consistent with the ATPase in-gel staining (Fig. 1D).

Next, since Aim11 interacts with the recently synthetized c-ring, we tried to analyze Atp9 migration in the Δ*aim11* strain. Unfortunately, Atp9 was not detectable with certainty as other ATP synthase subunits, likely due to its small size, hydrophobic peptides and low sequence coverage. However, what we observed is that Atp9 migrated mainly with the ATP synthase monomer and dimer (Fig. EV1) and was also detected in discreet populations from ~150 to 300 kDa (Fig. 6C). In contrast, the Δ*aim11* mutant Atp9 migrated at various size subcomplexes ranging from ~45 to 400 kDa. The more enriched population had a molecular mass of ~75 kDa, like the theoretical molecular mass of yeast c_10_-ring (77.6 kDa). Due to the suboptimal quality of Atp9 detection, we decided to look at the levels and migration of related proteins, as an indirect monitoring of Atp9 assembly. In yeast, Atp9 c-ring formation is mediated by Atp25 (Zeng et al, 2008), and then Atp9 is associated with Atp6 by the INA complex formed by Ina17 and Ina23 (Naumenko et al, 2017). Atp25 displayed a profile with several signals between 60 and 500 kDa (Figs. 6D and EV4D). However, in the Δ*aim11* mutant, two populations of Atp25 were clearly increased at ~80 and 120 kDa accompanied by a decrease in the rest of the signals. In the case of the INA complex, the Δ*aim11* mutation induced an increase in the population of Ina22 at around ~75 kDa (Fig. 6E), and an accumulation of a population of Ina17 at ~80 kDa. The accumulation of Atp25 and INA complex at low molecular mass could be the result of an alteration in the formation of the Atp9 ring in the Δ*aim11* mutant.

The [^35^S]-methionine in vivo labeling of Δ*aim11* cells showed an increase in the translation of *ATP6* mRNA compared to the WT strain (Fig. 1E). This same phenotype was observed in null mutants of *INA17* and *INA22* (Naumenko et al, 2017). Therefore, we asked if there was a possible genetic interaction between *AIM11* and *INA17* or *INA22*. We performed serial dilutions of the single mutants *Δaim11, Δina17*, and *Δina22*, plus the double mutants *Δaim11/Δina17* and *Δaim11/Δina22* (Fig. 6F)*.* Unlike published data (Lytovchenko et al, 2014), *Δina17* grew like the WT strain and the *Δina22* cells showed a slight respiratory growth defect (Fig. 6F). In contrast, the double mutant *Δaim11/Δina22* showed a decreased respiratory growth compared to the Δ*aim11* single mutant. This genetic interaction supports (Fig. 1E) a possible role of Aim11 in the formation of the membrane domain of ATP synthase.

Derived from our observations that Aim11 could modulate Atp9 and Atp6 biogenesis, we also looked for small-sized Atp6-containing sub-associations in our complexome dataset. Like Atp9, Atp6 was detected in low molecular mass subcomplexes (Fig. 6G). Some, like those of ~250 kDa, were enriched in the null mutant, while others appeared only in the mutant strain (~100 kDa population) (Fig. 6G). Atp6 assembly is assisted by the chaperone Atp10 and the protease Atp23 (Ackerman and Tzagoloff, 1990; Yang et al, 2021). Although the overall migration of Atp10 and Atp23 seemed similar between the WT and Δ*aim11* mitochondria, there were new peaks present in the null mutant, which might represent a compensatory mechanism in the Δ*aim11* strain (Fig. 6H). The third mitochondrial-encoded subunit Atp8 was not detected in either strain.

Finally, we asked if the rest of the ATP synthase subunits are also affected by the absence of Aim11. Yeast F_1_ domain is composed of the catalytic subunits α_3_ and β_3_, plus the central stalk subunits γ, δ and ε. There is a clear increase in α and β subunit levels at a molecular mass near 500 kDa (Fig. 6I). At the same molecular mass, the rest of the F_1_ domain plus OSCP also showed an increase in their levels (Fig. 6J).

Together with the published Aim11 pull-down data (Morgenstern et al, 2017, Data ref: Morgenstern et al, 2017), our data suggest that Aim11 interacts with Cox6 and the Atp9 c-ring, both components of the Atco complex. Although CIV integrity and activity seems to be more affected by the Δ*aim11* mutation, ATP synthase biogenesis is also clearly modulated by Aim11.

## Discussion

Our results indicate that Aim11 has pleiotropic effects on the OXPHOS machinery (Fig. EV1B), but the most sensitive component is CIV. Aim11 modulates CIV assembly, although it is not indispensable, as 30% of CIV activity is preserved after *AIM11* deletion (Fig. 1B). This observation suggests that other proteins can compensate for the absence of Aim11. Alternatively, there could be conditions different from those used in the present study where Aim11 acquires more relevance in the assembly of CIV. Despite the lower CIV activity, respiratory growth is not substantially affected by Aim11 absence (Fig. 1A). This could mean that lower levels of mature CIV are sufficient for yeast growth and do not represent a limiting step for ATP production and carbon source utilization (Boumans et al, 1998).

Biochemical approaches revealed that Aim11 does not significantly affect the production of mitochondrial-encoded CIV subunits (Cox1-3) (Fig. 1E). Instead, our data suggest that it has a role in the stability of Cox1 and, to a lesser degree, Cox2 (Fig. 1C). Complexome profiling of WT and Δ*aim11* mitochondria showed that assembly intermediates for all three CIV assembly modules accumulated. Cox2 was found in complex with Cox20 (~68 and 90 kDa) (Fig. 2D). Cox3 was associated with Rcf1 and Cox7 (~80 kDa), or only with Cox7 (~110 kDa), or only with Cox4 (~160 kDa) (Fig. 3E,F). Cox1 was enriched in two subassemblies (~90–100 kDa and ~160 kDa), both containing Cox5a, Cox8, Cox14, and Coa1. The bigger subassembly also had Coa3 and Mss51.

Our data are consistent with the idea that the main target of Aim11 is the hydrophilic subunit Cox6. The physical interaction between Aim11 and Cox6 was previously reported (Morgenstern et al, 2017, Data ref: Morgenstern et al, 2017). In the Δ*aim11* mutant, no Cox6 was detected in either Cox1 module subassembly, although a portion of Aim11 migrated along with free Cox6 (Figs. 2E and EV3C). In addition, *COX6* overexpression could partially compensate for the absence of Aim11, suggesting that Cox6 is a limiting step on CIV biogenesis (Fig. 2E,F). Therefore, we propose that Cox6 could interact with Aim11 before associating with the assembling Cox1 module. Since stability of Cox6 depends upon Aim11, in the Δ*aim11* mutant a portion of Cox6 is degraded, resulting in the accumulation of Cox1 intermediates and the overall observed lower levels of Cox1 (Figs. 2E and 3).

Aim11 does not act alone. Our results support the idea that it exerts its function in conjunction with Gep7, Iai11, and Mtc3, three proteins that were previously proposed to interact with Aim11 (Morgenstern et al, 2017, Data ref: Morgenstern et al, 2017). Due to its physical interaction and common phenotypes (Fig. 4), we named this protein complex as AMIGa. While Aim11 seems to be the main component of the complex, Iai11, Gep7, and Mtc3 function as ancillary factors, since the overexpression of *AIM11* complemented the absence of the other three proteins, but not the other way around (Figs. 4G and EV3F).

Besides Cox6 and the rest of the AMIGa complex, Aim11 and Iai11 copurified with several ATP synthase subunits, such as subunits α, β, δ, and ε (Morgenstern et al, 2017, Data ref: Morgenstern et al, 2017). Our complexome data showed the accumulation of several ATP synthase intermediates in a Δ*aim11* mutation, suggesting that Aim11 also regulates ATP synthase assembly, although it is not absolutely necessary (Fig. 6C–E,G–J). Previously, it was reported that an accumulation of free F_1_ domain promotes *ATP6* mRNA translation (Rak and Tzagoloff, 2009). This could explain the increase of *ATP6* mRNA translation in the Δ*aim11* mutant observed here (Fig. 1E). Despite this, the overall Atp6 and ATP synthase levels were not affected (Fig. 1C), suggesting that the extra Atp6 produced was degraded.

The role of Aim11 in complex V biogenesis is supported by the identification of TMEM242 as an Aim11 orthologue (Fig. 5), since TMEM242 is a human ATP synthase assembly factor (Carroll et al, 2021). Along with TMEM70, TMEM242 is responsible for the assembly of the membrane portion of the ATP synthase (Carroll et al, 2021). In the present work, we demonstrated that Aim11 interacts with the Atp9 c-ring, just like TMEM242 (Fig. 6A). In yeast, the membrane portion of ATP synthase requires the assembly factors Atp25, Atp23, Atp10, Ina17, and Ina22 (Franco et al, 2020b). Moreover, ATP synthase subunits or chaperones have been reported to interact physically (Atp23) (Naumenko et al, 2017, Data ref: Naumenko et al, 2017) or genetically (*ATP23, ATP10,* and *ATP18*) (Costanzo et al, 2010, 2016) with some of the AMIGa complex components. The Aim11/TMEM242 orthologue pair could be the first assembly chaperone of the membrane portion that is shared between humans and yeast, since no human orthologues of yeast Atp25, Atp23, Atp10, Ina17, and Ina22 have been identified. TMEM242 null mutant cell lines exhibited reduced levels of CIV and CIII_2_ complexes (Carroll et al, 2021), which is consistent with the observed phenotype of a Δ*aim11* mutant, where CIV and CIII_2_ activities were reduced (Fig. 1C).

**Figure 7:**
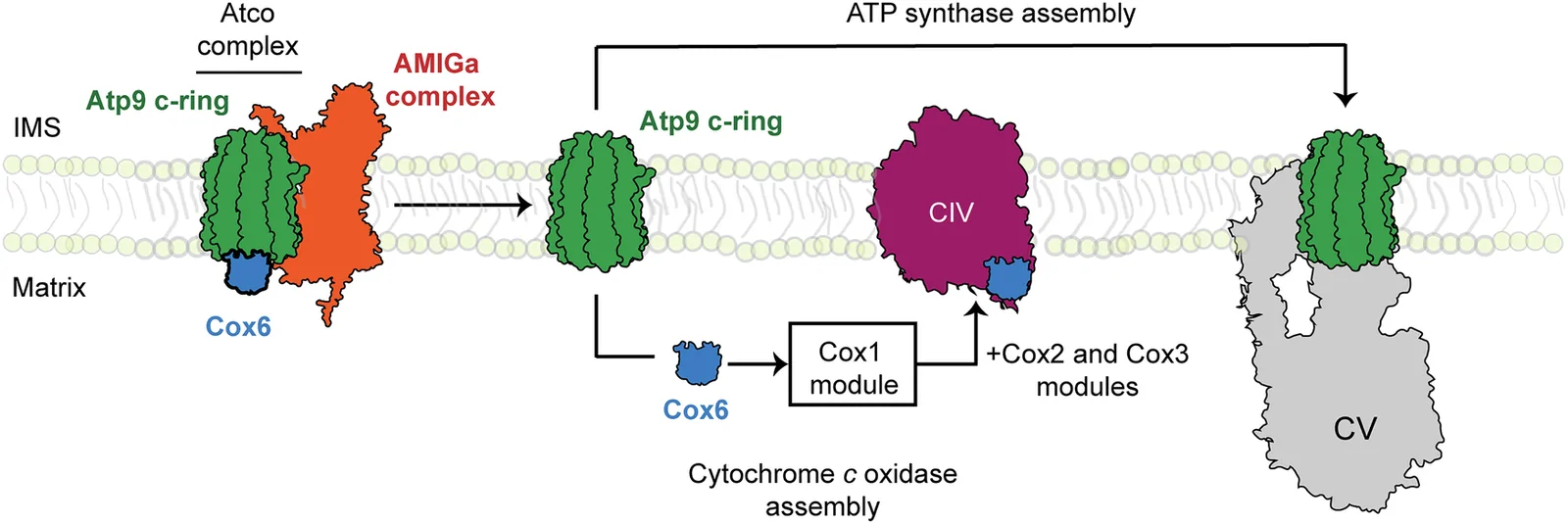
Model describing how the AMIGa complex regulates ATP synthase and cytochrome *c* oxidase biogenesis. The AMIGa complex interacts with the Atco complex containing Cox6 and the Atp9 c-ring. This interaction stabilizes Cox6 and, therefore, regulates the availability of this subunit and Atp9 for CIV and ATP synthase, respectively. Cox6 incorporates into the Cox1 assembly module, and the Atp9 c-ring incorporates into the ATP synthase. In addition, the AMIGa complex participates, directly or indirectly (possibly because of Cox1 module destabilization), in the assembly of the Cox2 and Cox3 modules.

How could the AMIGa complex have a role in both CIV and ATP synthase assembly? A possible explanation is through the Atco complex (see model in Fig. 7). Our data were consistent with a model in which Aim11 interacts with both components of this complex, Cox6 (Morgenstern et al, 2017, Data ref: Morgenstern et al, 2017) and the Atp9 c-ring. This complex could be formed prior to the incorporation of Cox6 and Atp9 into CIV and ATP synthase, respectively (Franco et al, 2023, 2020a). The group of Tzagoloff proposed that this shared intermediate works as a regulatory mechanism maintaining a proper ratio between the two complexes (Franco et al, 2020a). However, this regulation is unidirectional. The absence of Atp9 results in a defect on CIV levels, but a *COX6* null mutant does not affect ATP synthase levels (Franco et al, 2020a). This peculiarity is reminiscent of the phenotypes observed of the null mutant of Aim11, where CIV is affected but ATP synthase is not (Fig. 1D). Based on the fact that Aim11 interacts with both components, we propose a model where the AMIGa complex could function as a scaffold for the association of Cox6 and the Atp9 ring (Fig. 1E). In its absence, Cox6 is less likely to bind to the c-ring, and is thus degraded, resulting in the accumulation of both intermediates of CIV and ATP synthase. The unidentified peptide detected in mitochondrial translation assays could be derived from the *ATP9* mRNA and corresponds to a degradation product when the AMIGa complex is missing. Overexpression of *COX6* partially recovered the respiratory defect of a Δ*aim11* mutation, suggesting that the AMIGa complex participates in additional steps of the CIV biogenesis pathway. Our observations strongly support the involvement of the AMIGa complex in ATP synthase biogenesis, but the precise molecular mechanism remains unknown.

Why does the deletion of Aim11 result in many defects on CIV assembly, as well as CIII_2_ and ATP synthase alterations? During the peer review process of this article, a preprint focused on Aim11 was released (preprint: Kizmaz et al, 2026). The authors also validated the composition of the AMIGa complex and the physical interaction of Aim11 with Cox6. Additionally, a role of Aim11 in the biogenesis of inner membrane proteins was proposed (preprint: Kizmaz et al, 2026), related to its genetic interaction with prohibitins. The elimination of *AIM11* or *GEP7* are lethal or resulted in a growth defect in the absence of prohibitins (Osman et al, 2009). High-throughput assays showed that *IAI11* and *MTC3* also have a negative genetic interaction with either *PHB1* or *PHB2* (Costanzo et al, 2010, 2016). In yeast, the prohibitin ring has several functions, like a scaffold for stabilization of newly translated mitochondrial-encoded proteins (Nijtmans et al, 2000) or regulation of protein degradation (Steglich et al, 1999). In our complexome dataset, the prohibitin ring levels were increased in the *∆aim11* strain, possibly as a compensatory mechanism (Fig. EV1A). Coincidentally, null mutants of *COX6*, *ATP10*, and *ATP23* also have a growth defect when the prohibitin ring is absent (Osman et al, 2009, 2007). A possible explanation of the pleiotropic effects of Aim11 could originate from the common functions of this factor with those of prohibitins. Further work must be carried out to understand the relationship between prohibitins, the AMIGa complex and OXPHOS complexes assembly. Finally, the fact that the human orthologue TMEM242 can compensate for the *AIM11* null mutant, and that it is also present in flies and worms, suggests a conserved evolutionary role of the AMIGa complex.

## Methods

**Table:** Reagents and tools table

Reagent/resource	Reference or source	Identifier or catalog number
**Experimental models**		
BY4742 (*Saccharomyces cerevisiae*)	YKO Matα strain collection (Open Biosystems)	ATCC 201389 Table EV1
**Recombinant DNA**		
pRS416	Sikorski RS and Hieter P, 1989	GenBank: U03450.1
pGEm-T easy vector	Promega	A1360
Yep352	Hill et al, 1986	Genebank L14758
pVT100u-mtGFP	Westermann and Neupert, 2000	Addgene #45054
ZM467	Moqtaderi and Struhl, 2008	-
ZM474	Moqtaderi and Struhl, 2008	-
pMPY-HAx3	Schneider et al, 1995	-
pUG72	Gueldener, 2002	-
pUG73	Gueldener, 2002	-
pAG25	Goldstein and McCusker, 1999	Addgene #35121
pAG32	Goldstein and McCusker, 1999	Addgene #35122
pAG60	Goldstein and McCusker, 1999	Addgene #35128
Yeast expression plasmids	This study	Table EV3
**Antibodies**		
anti Mouse HRP	Santa Cruz	sc-2005
anti Rabbit HRP	Jackson Immunoresearch	111-035-003
Anti-FLAG M2-Peroxidase (HRP)	Sigma	A8592
Anti-FLAG M2	Sigma	F9291
Anti-HA Peroxidase HF	Roche	12013819001
Anti-HA High Affinity	Roche	11867423001
Anti V5 HRP	Sigma	V2260
Anti V5 Probe (E10)	Santa Cruz	sc-81594
**Oligonucleotides and other sequence-based reagents**		
PCR oligonucleotides	This study	Table EV2
**Chemicals, enzymes and other reagents**		
EcoRI	Invitrogen	IVGN0112
HindIII	Invitrogen	15207012
NotI	New England Biolabs	R0189 L
SphI	Promega	R626A
SalI	Invitrogen	152100-7011
T4 ligase	Thermo scientific	EL00 11
Yeast extract	BD	212750
Bacto Peptone	BD	211677
NaCl	JT Baker	3624-19
Dextrose	BD	215520
Ethanol	JT Baker	9014-02
Glycerol	JT Baker	2136
Galactose	Formedium	Gal-03
Lactic acid	Sigma	L1250
NaOH	Macron	1310732
Trizma base	Sigma	T1503
Lithium acetate	Sigma	L6883
5-FOA	Calbiochem	343922
Agarose	Sigma	A0169
Agar	BD	214040
Quiaprep spin miniprep	QIAGEN	27104
TIANgel purification kit	TIAGEN	GDP219-02
Quiaquick PCR purification kit	QIAGEN	28104
Protein A sepharose CL-4B	GE Healthcare	170780-01
TIANprep miniplasmid kit	TIAGEN	GDP103-02
RevertAid H minus first strand cDNA synthesis kit	Thermo Scientific	K1631
Zymolyase (20 T)	AMSBIO	120491-1
Immun-blot PVDF membrane	Bio-Rad	1620177
Autoradiography Film Ultra Cruz	Santa Cruz Biotechnology	sc-201696
Sodium dodecyl sulfate	Sigma	L5750
Sodium hydroxide	Macron	7708-10
HCl	JT Baker	9535-02
Acrylamide	Bio-Rad	1610107
Acrylamide 2x	SERVA	10675.02
Bis-acrylamide	Bio-Rad	1610201
*N*,*N’*-Methylene bis-acrylamide 2X	SERVA	29195.03
SDS	Sigma	L5750
TEMED	Sigma	T7024
DTT	Bio-Rad	1610611
Salmon sperm DNA solution	Invitrogen	15632011
PEG	Sigma	P4338
HEPES	Sigma	H3375
KH_2_PO_4_	Meyer	2055
K_2_HPO_4_	Macron	7092-04
Magnesium sulfate	JT Baker	2500-01
Horse heart cytochrome *c*	Sigma	C2506
ATP	Sigma	A3377
Lead nitrate	JT Baker	2322-50
Hygromycin B	Invitrogen	10687010
Nourseothricin sulfate	GoldBio	N-500-1
G418 Disulfate	Formedium	G4185
Drop out -URA	Formedium	DCS0169
Drop out -LEU	Bio 101	4510-522
Drop out -MET	Bio 101	4510-712
CSM	Bio 101	4500-022
YNB	BD	291940
EasyTag Methionine L- ^35^[S]	Perkin Elmer	NEG709A
Diamobenzidine	Fluka	32750
Digitonin	Sigma	D141
Coomassie Brilliant Blue G-250	Serva	35050.02
Prestained proteínas molecular weight marker	Thermo Scientific	26612
Precision Plus Protein WesternC Standards	Bio-Rad	161-0376
Precision Protein streptactin-HRP conjugate	Bio-Rad	161-0380
Native protein marker	GE healthcare	
PCRBIO HS Taq	PCRBIO	PB10.22-02
PCRBIO HI-Fi Taq	PCRBIO	PB10.41-02
Antimycin A	Sigma	A8674
Malonate	Sigma	M1296
Succinate	Sigma	S3674
GTP	Roche	1010639901
Alpha ketoglutarate	Sigma	K3752
Sorbitol	Sigma	S7547
Glycine	Sigma	G7126
Aminocaproic acid	Sigma	A2504
Tricine	Bio-Rad	1610713
Cycloheximide	Calbiochem	239764
Ethidium bromide	MP Biomedicals	190202
Glass beads	Thomas Scientific	5663RSO
DCPIP	Sigma	D1878
Sodium Cyanide	Sigma	380970
Imidazole ( ≥ 99%, molecular biology)	Sigma	I5513
Tricine	Sigma	T0377
6-aminohexanoic acid	Sigma	A2504
Methanol (HPLC grade)	Sigma	34860
Acetic acid (glacial, ReagentPlus®, ≥99%)	Sigma	A6283
Digitonin, water-soluble	SERVA	19551.02
Sodium chloride	Sigma	S7653
EDTA disodium salt	Sigma	E5134
Glycerol	Sigma	G5516
HPLC-grade water	VWR	83645.400
Trypsin, sequencing grade	Promega	V5111
Acetonitrile, LC-MS grade	Carl Roth	AE70.2
Formic acid, LC-MS grade	Thermo Fisher Scientific	28905
DL-Dithiothreitol	Sigma	D9779
Chloroacetamide	Sigma	C0267
MultiScreen-BV Filter Plate 1.2 µm	Merck Millipore	MABVN1250
Ammonium hydrogen carbonate LiChropur	Merck	5.33005
Emitters (15 cm L × 100 um ID × 360 um OD × 15 um orifice)	MS Wil CoAnn Technologies	ICT015100015-5
ReproSil-XR 120 C18, 3 µm	Dr. Maisch	rx13.9e
**Software**		
ImageQuant	Amersham	
Image Studio Digits	LI-COR	
ImageJ	NIH	
T-COFFEE	Jalview	
**Other**		
Typhoon 8600	GE Healthcare	
C-DIGIT	LI-COR	
Gradient maker	Hoefer	SG 50
Tissue grinder	Kimble	885300-0040
EASY-nLC 1000	Thermo Fisher Scientific	LC140
UltiMate 3000 UHPLC system	Thermo Fisher Scientific	RSLCnano / NCS-3500 / LPG-3400
Q Exactive Orbitrap MS	Thermo Fisher Scientific	MS07001
Q Exactive Plus Orbitrap MS	Thermo Fisher Scientific	IQLAAEGAAPFALGMBDK

### Yeast strains and growth conditions

Unless indicated otherwise, all *S. cerevisiae* strains are derived from the BY4742 background (ATCC 4040004). The strain list used in this study is Table EV1. The yeast cells were grown in rich media (2% peptone and 1% yeast extract) or synthetic media (CSM or specific dropout media) with different carbon sources (2% glucose, 2% galactose, 3% ethanol-glycerol, or 2% lactate, pH 5.5). For solid media, 2–2.2% agar was added.

Yeast transformation was performed using the lithium acetate method. G418-resistant mutant strains are from the YKO Matα strain collection (Open Biosystems). *kanMX4* cassettes were obtained from these mutants by PCR. Otherwise, the cassettes were amplified by PCR using pUG72, pUG73, pAG25, pAG32, or pAG60 plasmids as templates (Gueldener, 2002; Goldstein and McCusker, 1999).

For C-terminal epitope tagging of the proteins of interest, a *cis*-recombination approach was used as previously reported (Schneider et al, 1995; Moqtaderi and Struhl, 2008). The *3xtag-URA3-3xtag* construction was amplified using the pMPY-3xHA, ZM467 or ZM474 plasmids as templates. The sequences of the oligonucleotides used are listed in Table EV2.

### Plasmids construction

A list of plasmids generated in this study is listed in Table EV3. For yeast overexpression plasmids, the corresponding gene was amplified ~300 bp upstream and downstream of the coding sequence by PCR. The PCR products were ligated in the pGEM-T easy vector. Plasmids were digested with *EcoRI* or *SphI/SalI* and ligated to the YEp352 2 µ plasmid (Hill et al, 1986).

For the *TMEM242* overexpression plasmid, cDNA was obtained from RNA of the K562 human leukemia cell line (kindly donated by Dr. Mayra Furlan-Magaril lab). Then, the *TMEM242* coding sequence was amplified by fusion PCR to eliminate a *HindIII* site present at 309 bp from the *AUG* start codon. A FLAG epitope was added at the C-terminal end. The construction was digested with *NotI* and *HindIII* and ligated into the pVT100U-GFP vector (Westermann and Neupert, 2000). Expression of the gene was under the *ADH* promoter and terminator. Alternatively, the TMEM242 coding sequence was ligated between the *MSS51* promoter and terminator (~300 bp upstream and downstream of the coding region) in pRS416 using *NotI* and *HindIII*. All plasmids were sequenced using specific primers.

### Mitochondrial proteins analysis

Yeast cells were obtained in logarithmic phase (O.D. 1.0 ± 0.2) in galactose media, and mitochondria were isolated as previously reported (Diekert et al, 2001). Briefly, cells were treated with zymolyase. Afterward, spheroplasts were lysed by mechanical homogenization and crude mitochondria were obtained by differential centrifugation. Mitochondrial protein was quantified by a modified Lowry method (Markwell et al, 1978). Steady state levels were evaluated by SDS-PAGE (12%) and western blot using the indicated antibodies. Digitonin-solubilized mitochondria were used to separate mitochondrial complexes by BN-PAGE (4–12% gradient) (Wittig et al, 2006). The gel was used for cytochrome *c* oxidase or ATP synthase activities (see below) or transferred to a PVDF membrane for immunodetection. Primary antibodies used in this study are in Table EV4.

### In-gel cytochrome *c* oxidase and ATP synthase activity

For cytochrome *c* oxidase activity staining: the gel was placed in PBS buffer, pH 7.4 and horse cytochrome *c* (10 µg) and diaminobenzidine (50 µg) were added. The reaction was light-protected and incubated for 2 h in agitation. The reaction was stopped and destained by the addition of 10% methanol and 50% acetic acid (Wittig et al, 2007). For ATPase activity, the gel was placed in an incubation buffer (270 mM glycine, 35 mM Tris, pH 8.4) for 3 h. The buffer was replaced with activity buffer (270 mM glycine, 35 mM Tris, pH 8.4, 8 mM ATP, 14 mM MgSO_4_, 0.2% Pb(NO_3_)_2_), and incubated for several hours until a white precipitate was formed (Wittig et al, 2007).

### Immunoprecipitation assay

Isolated mitochondria (500 µg) were resuspended in 500 µL of lysis buffer (1% digitonin, 100 mM NaCl, 20 mM Tris, pH 7.4), incubated on ice for 10 min and clarified (10 min at 16,000×*g*). The supernatant was incubated with Sepharose beads coupled with specific antibodies (against HA or V5 epitope) for 2 h at 4 °C. The beads are then washed two times in lysis buffer and one time in 20 mM Tris, pH 7.4. Input fraction (equivalent to 10% of immunoprecipitation protein) and pulldown samples were run on SDS-PAGE conditions and transferred to a PVDF membrane for immunodetection.

### Mitochondrial translation assay

Mitochondrial-encoded protein synthesis in whole cells was carried out as previously reported (Perez-Martinez et al, 2003). For [^35^S]-methionine in vivo labeling, cells were grown in YPGal for two overnights at 30 °C. The cultures were diluted (1 mL in 10 mL of fresh media) and incubated for 3 h at 30 °C. Cells were centrifuged and washed with sterile water. The cell pellet was resuspended in a -Met drop-out media (2% galactose) and incubated for 30 min at 30 °C. Then 5 µL of cycloheximide (10 mg/mL) were added and incubated for 5 min. Labeling was initiated by adding 7 µCi of [^35^S]-methionine. After 15 min of labeling, cells were incubated in ice-water for 5 min and were mechanically lysed using glass beads. Mitochondrial fraction was obtained by differential centrifugation and resuspended in loading dye. The proteins were separated by SDS-PAGE and transferred to a PVDF membrane. The membrane was placed on a phosphor screen and scanned by Typhoon 8600 (GE Healthcare). ImageJ was used for densitometry analysis. De novo mitochondrial protein synthesis data were normalized using the Cox1 signal, and was presented as % of WT.

For in organello translation assay, isolated mitochondrial (250 µg) were preincubated in IoTL buffer (0.6 M sorbitol, 150 mM KCl, 15 mM phosphate buffer pH 7.4, 20 mM Tris, pH 7.4, 12.5 mM MgSO_4_, 3 mg/mL BSA,12 µg/mL of cysteine, and methionine free amino acid mix, 6.6 µM cysteine, 6.6 µg/mL cycloheximide, 1.13 mg/mL α-ketoglutarate acid, 2.3 mg/mL phosphoenolpyruvate, 4 mM ATP, 0.5 mM GTP, and 0.13% pyruvate kinase) for 5 min at 30 °C. Labeling was started by the addition of [^35^S]-methionine (25 µCi) for 20 min at 30 °C. The reaction was stopped with 5 µL of unlabeled methionine (100 mM). For the total fraction, 5% of the samples were saved. Mitochondria were washed in SH buffer (0.8 M Sorbitol, 20 mM HEPES, pH 7.4). Samples were resuspended in lysis buffer (1% digitonin, 100 mM NaCl, 20 mM Tris, pH 7.4), incubated on ice for 10 min and clarified (10 min at 16,000×*g*). The supernatant was incubated with antibodies against the FLAG epitope coupled to Sepharose beads for 2 h at 4 °C. Beads were washed twice in lysis buffer and once in 20 mM Tris, pH 7.4. Proteins were separated by SDS-PAGE and transferred to a PVDF membrane. The membrane was placed on a phosphor screen and scanned with a Typhoon 8600 (GE Healthcare).

### Enzymatic activity assays

Respiratory complex activity was performed as previously reported (Flores-Mireles et al, 2023). In brief, complex II activity was evaluated by measuring DCPIP reduction, while complex III_2_ and IV activity were estimated by measuring the reduction or oxidation of cytochrome *c*, respectively. All determinations were made at 25 °C, using a protein concentration of 20 μg/mL. Specific inhibitor-sensitive activities were obtained by subtracting the inhibitor-resistant activity (malonate, antimycin A or cyanide) and normalized with the protein content. The WT strain activity was considered as 100% activity. The data was analyzed by Student’s *t*-test.

### Complexome profiling

**Figure EV5:**
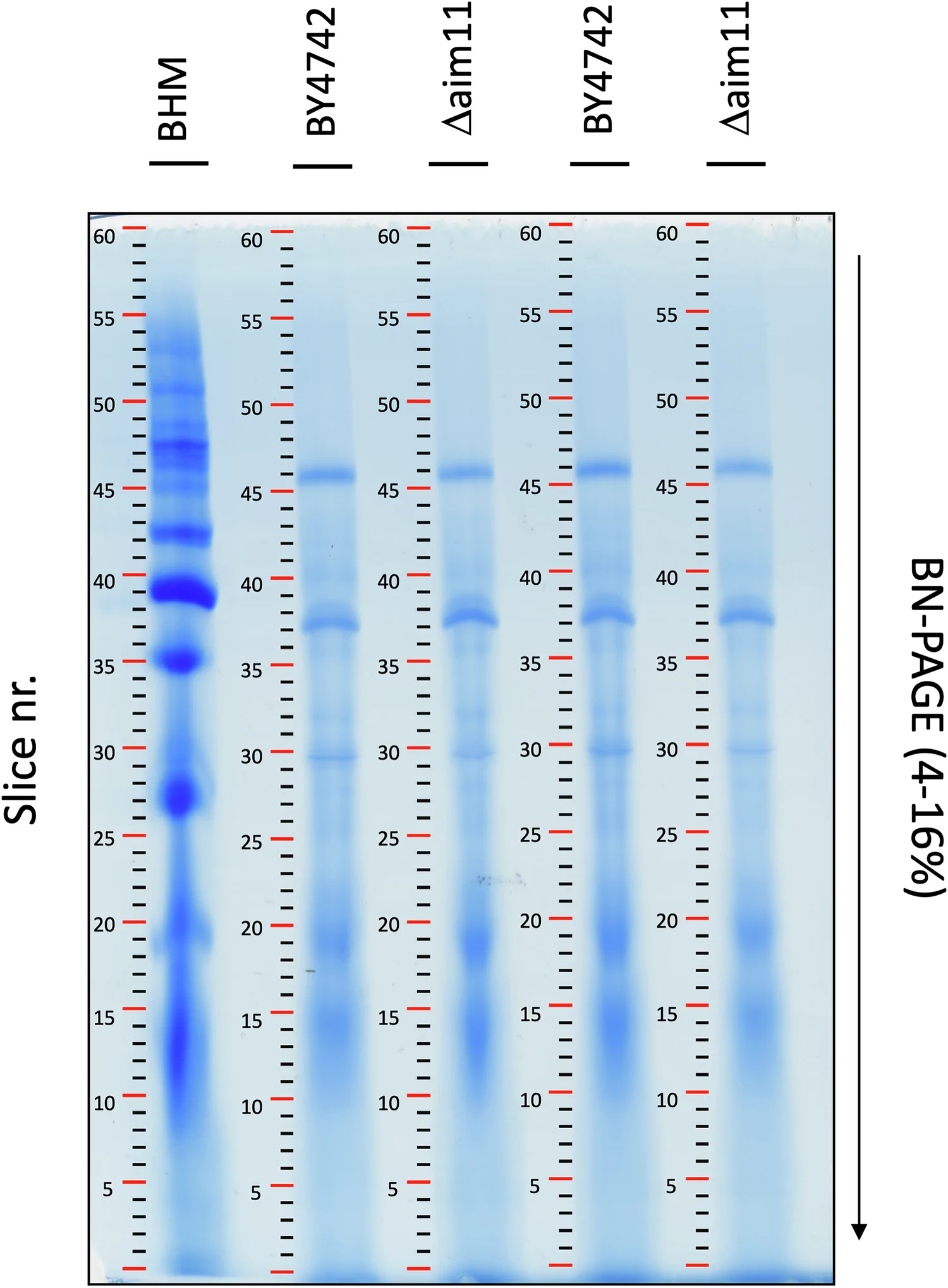
Sample BN-PAGE gel used for complexome profile. 100 μg of mitochondrial protein were loaded in each lane. BN-PAGE conditions were as described in the Materials and Methods section. Bovine heart mitochondria (BHM) were used as a molecular mass reference.

Isolated mitochondria (100 µg) were solubilized with research-grade water-soluble digitonin (3 mg/mg protein; SERVA GmbH) and separated by BN-PAGE (4–16% gradient) (FIg. EV5) (Wittig et al, 2006). Afterward, the gel was fixed, stained, destained, rehydrated and processed for complexome profiling analysis as formerly described (Flores-Mireles et al, 2023). Briefly, the gel was scanned and cut into 60 or 48 identical slices. Each slice was further diced, reduced, alkylated, and trypsin digested. The resulting peptides were separated by liquid chromatography (UHPLC) and analyzed by tandem mass spectrometry (MS/MS). Each condition was analyzed in at least two independent replicates. Then the data obtained were matched in MaxQuant (v2.6.1.0) against the reference proteome of *S. cerevisiae* (UP000002311, downloaded on 08.02.2024). The abundance of each protein was determined from absolute intensity-based quantification values (iBAQ). To facilitate visualization, iBAQ values were converted into fractional abundances normalized to a maximum value of 1, either globally across all gel fractions or restricted to a defined mass range when the protein of interest migrated within a defined molecular mass window. To enable comparison of different samples processed in independent gel runs, complexome profiles were aligned using COPAL (Van Strien et al, 2019). Finally, after hierarchical clustering of the complexome dataset using Pearson correlation, mitochondrial proteins of interest were analyzed and visualized as heat maps or line charts in Microsoft Excel.

### Bioinformatic analysis

For the search for Aim11 metazoan orthologues, the HHPRED platform was used (Zimmermann et al, 2018)*. S. cerevisiae* Aim11, Iai11, Gep7, and Mtc3 protein sequences were used to mine the *Homo sapiens, Caenorhabditis elegans and Drosophila melanogaster* proteomes, using standard parameters. Human TMEM242 was searched against the yeast proteome as an inverse query. Phobius (Kall et al, 2007) was used to predict Aim11 and TMEM242 transmembrane domains. The sequences of yeast Aim11 (UniProt code P87275) and human TMEM242 (UniProt code Q9NWH2) were aligned using the T-COFFEE algorithm in Jalview (Waterhouse et al, 2009). 3D structure prediction of Aim11 and TMEM242 was modeled using AlphaFold3 (Abramson et al, 2024).

### Experimental sample size and statistical analysis

All experiments included three independent biological replicates, except for complexome profiling, which included two independent replicates. The densitometries (Fig. 1E) were obtained using ImageJ. Statistical analysis performed by multiple unpaired *t*-test analysis to compare each newly synthesized protein to the control (WT), *p < 0.05 vs WT. In Fig. 1B, statistically significant changes were determined using a student’s *t*-test analysis; **p < 0.01, ***p < 0.001 (*n* = 3 independent preparations; nine technical replicates). The statistical analysis was unblinded.

## Supplementary information

Table EV1

Table EV2

Table EV3

Table EV4

Peer Review File

Expanded View Figures

## Data Availability

The complexome MS raw data underlying Figs. 2–4 and 6 and EV1–4 have been deposited to the ProteomeXchange Consortium via the PRIDE (Perez-Riverol et al, 2025) partner repository with the dataset identifier PXD075467. Source data can be found at: https://www.ebi.ac.uk/biostudies/studies/S-BSST3189?query=S-BSST3189. The source data of this paper are collected in the following database record: biostudies:S-SCDT-10_1038-S44319-026-00946-7.
