# Supplementary material for: The AMIGa complex is at the crossroads of cytochrome c oxidase and ATP synthase biogenesis
Source: EMBO Rep. 2026 Oct 6;27(21):6115–38. doi: 10.1038/s44319-026-00946-7 (PMC13743771; doi:10.1038/s44319-026-00946-7)
Supplement: Supplementary file 1 [file 44319_2026_946_MOESM5_ESM.pdf]

EMBO reports

The AMIGa complex is at the crossroads of cytochrome c oxidase and ATP synthase biogenesis

Ulrik Pedroza-Dávila, Yolanda Camacho-Villasana, Miriam Vázquez-Acevedo, Madhurya Lutikurti, Diego González-Halphen, Xochitl Pérez-Martínez, and Alfredo Cabrera-Orefice

Corresponding author(s): Xochitl Pérez-Martínez (xperez@ifc.unam.mx) , Ulrik Pedroza-Dávila (upedroza@ifc.unam.mx)

Review Timeline:

Submission Date:	14th Nov 25
Editorial Decision:	12th Dec 25
Revision Received:	5th Jun 26
Editorial Decision:	17th Aug 26
Revision Received:	28th Aug 26
Accepted:	11th Sep 26

Editor: Kurt Weir / Martina Rembold

Transaction Report:

(Note: With the exception of the correction of typographical or spelling errors that could be a source of ambiguity, letters and reports are not edited. Depending on transfer agreements, referee reports obtained elsewhere may or may not be included in this compilation. Referee reports are anonymous unless the Referee chooses to sign their reports.)

Dear Dr. Pérez-Martínez

Thank you for the submission of your research manuscript to our journal. We have now received the full set of referee reports that is copied below.

As you have seen, the referees acknowledge that the findings are interesting and that the conclusions are overall supported by the data presented but they also raise a number of concerns and have suggestions how to further strengthen the data, particularly the inclusion of pull-down experiments and increased discussion of a number of aspects of the paper.

Given these constructive comments, we would like to invite you to revise your manuscript with the understanding that the referee concerns (as detailed above and in their reports) must be fully addressed and their suggestions taken on board. Please address all referee concerns in a complete point-by-point response. Acceptance of the manuscript will depend on a positive outcome of a second round of review. It is EMBO Reports policy to allow a single round of revision only and acceptance or rejection of the manuscript will therefore depend on the completeness of your responses included in the next, final version of the manuscript.

In addition, we encourage you to post the initial, though not the revised, version of the manuscript to BioRxiv.

We realize that it is difficult to revise to a specific deadline. In the interest of protecting the conceptual advance provided by the work, we recommend a revision within 3 months (March 12th). Please discuss the revision progress ahead of this time with the editor if you require more time to complete the revisions.

I am also happy to discuss the revision further via e-mail or a video call, if you wish.

You can either publish the study as a short report or as a full article. For short reports, the revised manuscript should not exceed 27,000 characters (including spaces but excluding materials & methods and references) and 5 main plus 5 expanded view figures. The results and discussion sections must further be combined, which will help to shorten the manuscript text by eliminating some redundancy that is inevitable when discussing the same experiments twice. For a normal article there are no length limitations, but it should have more than 5 main figures and the results and discussion sections must be separate. In both cases, the entire materials and methods must be included in the main manuscript file.

================
IMPORTANT NOTE:

We perform an initial quality control of all revised manuscripts before re-review. Your manuscript will FAIL this control and the handling will be delayed IN CASE the following APPLIES:

1) A data availability section providing access to data deposited in public databases is missing. If you have not deposited any data, please add a sentence to the data availability section that explains that.

2) Your manuscript contains statistics and error bars based on n=2. Please use scatter blots in these cases. No statistics should be calculated if n=2.

When submitting your revised manuscript, please carefully review the instructions that follow below. Failure to include requested items will delay the evaluation of your revision.

================

When submitting your revised manuscript, we will require:

1) a .docx formatted version of the manuscript text (including legends for main figures, EV figures and tables). Please make sure that the changes are highlighted to be clearly visible.

2) individual production quality figure files as .eps, .tif, .jpg (one file per figure).
Please download our Figure Preparation Guidelines (figure preparation pdf) from our Author Guidelines pages https://link.springer.com/journal/44319/submission-guidelines for more info on how to prepare your figures.

3) a .docx formatted letter INCLUDING the reviewers' reports and your detailed point-by-point responses to their comments. As part of the EMBO Press transparent editorial process, the point-by-point response is part of the Review Process File (RPF), which will be published alongside your paper.

4) a complete author checklist, which you can download from our author guidelines (
5) Please note that all corresponding authors are required to supply an ORCID ID for their name upon submission of a revised manuscript (). Please find instructions on how to link your ORCID ID to your account in our manuscript tracking system in our Author guidelines
()

6) We replaced Supplementary Information with Expanded View (EV) Figures and Tables that are collapsible/expandable online. A maximum of 5 EV Figures can be typeset. EV Figures should be cited as 'Figure EV1, Figure EV2" etc... in the text and their respective legends should be included in the main text after the legends of regular figures.

- For the figures that you do NOT wish to display as Expanded View figures, they should be bundled together with their legends in a single PDF file called *Appendix*, which should start with a short Table of Content. Appendix figures should be referred to in the main text as: "Appendix Figure S1, Appendix Figure S2" etc. See detailed instructions regarding expanded view here:

- Additional Tables/Datasets should be labeled and referred to as Table EV1, Dataset EV1, etc. Legends have to be provided in a separate tab in case of .xls files. Alternatively, the legend can be supplied as a separate text file (README) and zipped together with the Table/Dataset file.

7) Before submitting your revision, primary datasets (and computer code, where appropriate) produced in this study need to be deposited in an appropriate public database (see <
https://www.embopress.org/page/journal/14693178/authorguide#dataavailability>).

Please remember to provide a reviewer password if the datasets are not yet public.

The accession numbers and database should be listed in a formal "Data Availability " section (placed after Materials & Method) that follows the model below (see also < https://www.embopress.org/page/journal/14693178/authorguide#dataavailability>). Please note that the Data Availability Section is restricted to new primary data that are part of this study.

Data availability

The datasets (and computer code) produced in this study are available in the following databases:

- RNA-Seq data: Gene Expression Omnibus GSE46843 (https://www.ncbi.nlm.nih.gov/geo/query/acc.cgi?acc=GSE46843)
- [data type]: [name of the resource] [accession number/identifier/doi] ([URL or identifiers.org/DATABASE:ACCESSION])

*** Note - All links should resolve to a page where the data can be accessed. ***

8) At EMBO Press we ask authors to provide source data for the main manuscript figures. You will receive a separate email with instructions for providing source data with your revised manuscript, including how to upload and organize the files.

Additional information on source data and instruction on how to label the files are available

9) The journal requires a statement specifying whether or not authors have competing interests (defined as all potential or actual interests that could be perceived to influence the presentation or interpretation of an article). In case of competing interests, this must be specified in your disclosure statement. Further information: https://www.embopress.org/competing-interests

10) Figure legends and data quantification:
The following points must be specified in each figure legend:

- the name of the statistical test used to generate error bars and P values,
- the EXACT p-values,
- the number (n) of independent experiments (please specify technical or biological replicates) underlying each data point,
- the nature of the bars and error bars (s.d., s.e.m.)

- If the data are obtained from n {less than or equal to} 5, show the individual data points in addition to the SD or SEM.
- If the data are obtained from n {less than or equal to} 2, use scatter blots showing the individual data points.

Discussion of statistical methodology can be reported in the materials and methods section, but figure legends should contain a basic description of n, P and the test applied.

See also the guidelines for figure legend preparation:
https://www.embopress.org/page/journal/14693178/authorguide#figureformat

- Please also include scale bars in all microscopy images and define their size in the legend, not the image.

11) Our journal encourages inclusion of *data citations in the reference list* to directly cite datasets that were re-used and obtained from public databases. Data citations in the article text are distinct from normal bibliographical citations and should directly link to the database records from which the data can be accessed. In the main text, data citations are formatted as follows: "Data ref: Smith et al, 2001" or "Data ref: NCBI Sequence Read Archive PRJNA342805, 2017". In the Reference list, data citations must be labeled with "[DATASET]". A data reference must provide the database name, accession number/identifiers and a resolvable link to the landing page from which the data can be accessed at the end of the reference. Further instructions are available at .

12) All Materials and Methods need to be described in the main text using our 'Structured Methods' format. According to this format, the Methods section includes a Reagents and Tools Table (listing key reagents, experimental models, software and relevant equipment and including their sources and relevant identifiers) followed by a Methods and Protocols section describing the methods, ideally using a step-by-step protocol format. The aim is to facilitate adoption of the methodologies across labs. Please download and fill our Reagents and Tools Table template (.docx), which you can find in our author guidelines: https://www.embopress.org/page/journal/14693178/authorguide#structuredmethods.
When submitting your revised manuscript, please do not include the Reagents and Tools Table in the Methods section of the manuscript but upload it as a separate file choosing the file type "Reagent Table".
An example of a Method paper with Structured Methods can be found here: https://www.embopress.org/doi/10.15252/msb.20178071.

13) As part of the EMBO publication's Transparent Editorial Process, EMBO Reports publishes online a Review Process File to accompany accepted manuscripts. This File will be published in conjunction with your paper and will include the referee reports, your point-by-point response and all pertinent correspondence relating to the manuscript.

You are able to opt out of this by letting the editorial office know (emboreports@embo.org). If you do opt out, the Review Process File link will point to the following statement: "No Review Process File is available with this article, as the authors have chosen not to make the review process public in this case."

We would also welcome the submission of cover suggestions, or motifs to be used by our Graphics Illustrator in designing a cover.

I look forward to seeing a revised form of your manuscript when it is ready and please let me know if you have questions or comments regarding the revision.

Yours sincerely,

Kurt Weir
Editor
EMBO Reports

Referee #1:

The assembly of the cytochrome c oxidase is a multi-step process that involves several assembly factors, intermediates and pathways. In this paper, the authors propose that Aim11 is a new assembly factors of complex IV. The idea is that Aim11 forms a complex with Mtc3, Gep7 and Iai11 to promote efficient assembly of complex IV. The conclusion is based on growth defects on respiratory medium, comigration on complexome profiling and activity assays. Overall, the findings are potentially interesting. However, further experimental work is required to show the role of these factors in the formation of complex IV.

1. The role of Aim11 in the assembly of complex IV is based on its comigration with complex IV subunits and Mtc3, Gep7 and Iai11. However, co-migration on complexome profiling does not necessary mean that the proteins interact. The authors should perform pulldowns to confirm the proposed interactions.
2. The authors used complexome profiling to propose protein interactions. As mentioned under point 1, this approach has its limitation and requires validation by other experimental means. Furthermore, the authors should cite and use the published data from high-resolution complexome profiling of yeast mitochondria (Schulte et al. 2023 Nature) to compare it with their complexome profile.
3. In Figure 3, it is hard to see the COX1 and COX3 modules. The authors should use additional tools like pulldown to confirm the presence f these modules.
4. Figure 1D; the blue native gels are of poor quality and should be replaced.

5. Figure 4: The authors should show blue native gels for the used deletion mutants to revel their effect for complex IV formation.
6. Figure 1E: Can the authors speculate why the expression of ATP6 is strongly increased in aim11delta?

Referee #2:

The manuscript entitled "Aim11 is a novel protein involved in the assembly of mitochondrial cytochrome c oxidase" by Ulrik Pedroza-Dávila, Yolanda Camacho-Villasana, Miriam Vázquez-Acevedo, Madhurya Lutikurti, Diego González-Halphen, Alfredo Cabrera-Orefice and Xochitl Perez-Martinez1 characterizes the role of Aim11 in cytochrome oxidase assembly and provides a valuable workflow for dissecting the refinement steps of mitochondrial biogenesis. The aim11 mutant shows slower respiratory growth and reduced steady-state levels of CIV compared to the wild-type strain. The authors separated mitochondrial protein extracts from the WT and aim11 mutant strains in non-denaturing gel by BN-PAGE, cutting it into over 40 slices and subjecting each to mass spectrometry, an approach that allowed them to identify interactions within CIV and its stable subassemblies. This is a robust, well-designed study that may help elucidate the functions of proteins that participate in, but are not essential for, respiration. This is an important contribution given that about 20% of yeast proteins still lack functional characterization. In addition to confirming previous data from the Tzagoloff lab obtained using other techniques (such as radiolabeling) regarding cytochrome oxidase assembly modules and intermediates, the authors present new findings, including the identification of a role for Aim11 in CIV biogenesis. They show an accumulation of CIV assembly intermediates in the aim11 mutant, demonstrate that Aim11 interacts with the uncharacterized proteins Mtc3, Gep7, and Iai11, all of which contribute together to efficient CIV assembly, and identify the human protein TMEM242 as the orthologue of Aim11. Overall, the manuscript is original, presenting findings that provide new insights to the field, and it is well written, with a clear structure and coherent presentation of the data.

Major points:

I am surprised that such a drastic reduction in steady-state CIV levels (Figure 1D) results in only a mild respiratory phenotype in the spot test. Do the authors have any idea of what might explain this?

What is the percentage of rho-/rho0 of aim11 mutant in ethanol-glycerol and lactate? The Saccharomyces Genome Database (SGD) reports that the null mutant exhibits increased loss of mitochondrial genome. Is this phenotype in the present study due to unstable mtDNA or to reduced CIV levels? Figure 1E suggests that mtDNA is stable, but the authors should include the percentage of rho^{-}/rho^{0} cells and comment on any differences relative to the phenotype described in SGD.

I find it very interesting that there is an apparent decrease in the Atp9 ring (Figure 1E), given that Atp9 was shown to form an assembly intermediate together with Cox6 that couples the biogenesis of cytochrome oxidase and ATP synthase (Franco et al, 2023). Cox6, in turn, is part of the Cox1 assembly module. Combined with the fact that Aim11 interacts with Cox6, please comment on that.
Also, is it possible that the absence of Aim11 is preventing Cox6 from properly interacting with Atp9, thereby causing the accumulation of these Cox1-containing subassemblies (Figure 2B)?

Minor points:

Please note that some references are repeated:
(Veloso et al, 2018; Su et al, 2014b; Franco et al, 2018; McStay et al, 2013b).
Franco LVR, Su C-H, McStay GP, Yu GJ & Tzagoloff A (2018) Cox2p of yeast cytochrome oxidase assembles as a stand-alone subunit with the Cox1p and Cox3p modules. J Biol Chem 293: 16899-16911
Veloso L, Franco R, Su C-H, Mcstay GP, Yu GJ & Tzagoloff A (2018) Cox2p of yeast cytochrome oxidase assembles as a stand-alone subunit with the Cox1p and Cox3p modules.

"Aim11 is a mitochondrial inner membrane protein of ~15 kDa with two transmembrane helices (Morgenstern et al, 2017)" - There is a period missing after this sentence.

"We asked whether the absence of Aim11 affected CIV function or assembly. Thus, we analyzed the steady state levels of CIV subunits (Cox1-3)" - The problem lies in the sentence (not in the experiments themselves). The authors should also mention the BN gel, in which they show that there is indeed much less CIV, and not only lower levels of its subunits.

In Figure 1A, it would be helpful to show the rich-media growth after just one day.

For Figure 1D, it may improve the clarity of the figure if the ATPase in-gel assay is moved from below the Cox1 western. Then, the authors could add the label "ATPase in-gel assay" at the top of the figure, along with the strain names.

For Figure 1E, please include (Atp9)n in the "% of WT" graph, as it appears to be decreased.

For the Figure 2 legend, please indicate whether the plates correspond to complex or synthetic media (they appear to be synthetic).

In Figure 4B, the double mutant displays higher levels of Cox1, Cox2, and Cox3 compared with the single aim11 mutant. What could be the underlying mechanism?

In Figure 4C, it appears that the Δiai11, Δgep7, and Δmtc3 mutants show higher levels of newly translated Cox1, yet reduced steady-state Cox1 levels. Please comment on this.

"TMEM242 from the K562 cell line into a 2 μ yeast expression vector under the ADH promoter and terminator. Then, the plasmid was transformed into different yeast mutants. TMEM242 compensated for the respiratory growth in the Δaim11, Δgep7, Δiai11, and Δmtc3 mutants (Fig 5C)." - It would be important to clarify whether TMEM242 compensates for the Δaim11 phenotype only when overexpressed. Or does it also compensate when expressed from an integrative vector?

"While Aim11 is the main assembly factor, Iai11, Gep7, and Mtc3 function as accessory factors, since the overexpression of AIM11 complemented the absence of the other three proteins." - The authors should mention the supplementary figure in which they show this result.

Referee #3:

This manuscript investigates the role of the uncharacterized mitochondrial inner membrane protein Aim11 in the biogenesis of cytochrome c oxidase (Complex IV, CIV) in Saccharomyces cerevisiae. Through growth assays, respiratory chain enzymology, immunoblotting, [35S]-pulse labeling, and especially complexome profiling, the authors conclude that Aim11 participates in the assembly of all three CIV modules (Cox1-, Cox2-, and Cox3-modules). They further show that Aim11 forms a complex with three poorly characterized mitochondrial proteins (Iai11, Gep7, Mtc3) and propose that Aim11 is the "principal" component of an assembly complex. Finally, they present functional cross-species complementation by human TMEM242.
The manuscript addresses an important problem in mitochondrial biology: identifying new components of CIV biogenesis and understanding how modular assembly is integrated. The complexome profiling dataset is extensive and potentially impactful. However, as currently written, the manuscript has significant weaknesses in mechanistic interpretation, experimental rigor, and clarity of presentation, which limit its suitability for EMBO Reports without major revision.

Major Comments
1. Mechanistic insight is insufficiently developed, and the proposed Aim11 roles remain descriptive. The manuscript repeatedly claims that Aim11 "assists progression" of all three CIV modules, yet the mechanistic basis is unclear. The key findings (accumulation of module-specific intermediates; lower Cox1/Cox2 levels; disappearance of Cox6-Aim11 complex; partial rescue by COX6 overexpression) are consistent with defects in late membrane integration and/or stabilization, but do not pinpoint whether Aim11 acts: (i) as a Cox6 chaperone, (ii) as a membrane insertase/holdase, (iii) as a scaffold for module progression, (iv) as a stability factor for multiple assembly modules, (v) or indirectly via inner membrane homeostasis/prohibitins. The current data allow several interpretations. Additional experiments are needed to clarify the role of Aim11 and its potential effects on all three CIV modules simultaneously.

2. Low-quality BN-PAGE data that weaken complexome profile studies. The authors report a large amount of free CIV, even in the WT strain, under their complex extraction conditions, which indicates over-solubilization of the membranes and disruption of the supercomplexes. They used a digitonin:protein ratio of 3:1, but it is not indicated whether they used high-purity digitonin. Impurities are well known to disrupt membranes in a rather uncontrolled manner. In addition to using high-purity digitonin, I suggest including additional bona fide CIV mutants as controls, as the membrane solubilization conditions may be too harsh, and some peripheral subunits (such as Cox12) may be ripped from the complex.

3. Complexome profiling interpretation needs more rigor and quantitation. Complexome analysis is a major strength, but the interpretation is partly qualitative. Specific issues include:
3.1. Quantitation is lacking. For example, "~70% decrease" in supercomplexes requires numerical data, error bars, and statistical testing. Area-under-the-curve quantifications must be shown and methods described in detail.
3.2. Subcomplex assignments are overconfident. Most "subassemblies" are assigned based solely on comigration, though large shifts suggest missing partners. Without immunoprecipitation or crosslinking evidence, the manuscript should avoid definitive statements about composition. Orthogonal approaches are absolutely needed.
3.3. The essential distinction between assembly intermediates and degradation fragments is not addressed
Cox1 and Cox3 low-mass species could represent proteolysis fragments (common when assembly stalls). This must be considered.
3.4. The claim that Aim11 affects all three modules equally is not quantitatively supported
Cox1 appears most severely affected; Cox3 module effects seem secondary.

4. The interpretation of [35S]-labeling experiments is problematic. The authors conclude that mitochondrial translation is not

impaired. However, (i) Cox1 synthesis appears unchanged (bypassing CIV-dependent COX1 mRNA translation regulation), yet steady-state Cox1 is drastically lower, suggesting rapid turnover. (ii) Cox2 and Cox3 synthesis increase, which is a hallmark of imbalanced module assembly. (iii) Atp6 synthesis dramatically increases, but ATP synthase levels do not, suggesting broader perturbation of membrane protein homeostasis.
These data strongly suggest an inner membrane protein quality-control defect rather than selective CIV assembly failure. This must be addressed explicitly.

5. A critical experiment is missing: Cox6 stability and turnover. According to the data presented, Cox6 is the most compelling Aim11-related target. Cox6 steady-state levels are reduced; Aim11-Cox6 comigrate in complexome data; COX6 overexpression partially rescues growth; and Gep7 and Mtc3 share structural similarity with Cox5/Cox4 family proteins. However, Cox6 stability or half-life is never measured. Cycloheximide chase or pulse-chase assays for Cox6 are essential to clarify the model.

6. The link between Aim11 and prohibitins is underdeveloped. Aim11 is synthetic lethal with prohibitins, but the manuscript does not integrate this observation. Prohibitins shape inner membrane domains and regulate the insertion of Cox1/Cox3, as shown in several papers. Aim11 may act in the same membrane quality-control network. The discussion should address this explicitly.

7. The low-molecular-mass polypeptide observed in all mutants is never identified. This is repeatedly mentioned as "unknown". Is it a proteolytic product? An unprocessed mitochondrial protein? A marker of membrane stress? At a minimum, the authors need to attempt identification (LC-MS/MS) or provide a reasonable hypothesis.

8. The authors refer to the Aim11-Mtc3-Gep7-Iai11 complex as a demonstrated fact, but the only experimental evidence presented is co-sedimentation. While for subunits already known to belong to the same enzyme, it may be sufficient to moderate the conclusion statements, for this new "complex," an IP assay is absolutely necessary. Also, are the other components interacting in the absence of Aim11?

9. The study does not explain why Aim11 affects CIII2 activity. Complex III2 activity decreases to 70% of wild-type. The authors attribute this to CIV-related defects, but this is rather speculative. In the literature, some CIV assembly mutants have increased rather than decreased CIII activity. Again, the discussion must address this point and how it fits into the current model, and consider whether the model should be broadened to include all observations.

Minor Comments
1. Discussion of Cox12 dissociation during BN-PAGE should more clearly reference the literature.
2. The paper should clearly distinguish "association" vs. "true physical interaction", especially for complexome-derived inferences.
5. BN-PAGE solubilization conditions (digitonin purity) must be reported.
6. The normalization strategy for the complexome profiling dataset is not described.
7. Details of statistical analyses are lacking.
8. As a suggestion, the authors may consider reorganizing the figures to emphasize a clear assembly model rather than exhaustive cataloguing of intermediates.

Message to all reviewers:

To find the space to thoroughly address all reviewers concerns we decided, with the consent of the editor, Dr. Weir, to switch the manuscript from a report to a full-length article.

Some of the main changes that we included in the revised version of the manuscript are experiments exploring the additional role of Aim11 in ATP synthase biogenesis, and how these observations are connected to CIV assembly. In this sense we decided to change the title of the manuscript to "The AMIGa complex is at the crossroads of cytochrome *c* oxidase and ATP synthase biogenesis" instead of "Aim11 is a novel protein involved in the assembly of mitochondrial cytochrome *c* oxidase". The inspiration to make these changes comes from the fact that, as pointed out by reviewer 3, lack of Aim11 seems to increase Atp6 synthesis. In addition, the human orthologue that we identified, TMEM242, has a reported role in ATP synthase biogenesis. We also decided to name the Aim11-Mtc3-Iai11-Gep7 assembly complex as "AMIGa complex", and more deeply explored and discussed the role of this complex on Cox6 and the Atp9 c-ring complex (the Atco complex previously described by Veloso and Tzagoloff).

Referee 1

The assembly of the cytochrome c oxidase is a multi-step process that involves several assembly factors, intermediates and pathways. In this paper, the authors propose that Aim11 is a new assembly factors of complex IV. The idea is that Aim11 forms a complex with Mtc3, Gep7 and Iai11 to promote efficient assembly of complex IV. The conclusion is based on growth defects on respiratory medium, comigration on complexome profiling and activity assays. Overall, the findings are potentially interesting. However, further experimental work is required to show the role of these factors in the formation of complex IV.

1. The role of Aim11 in the assembly of complex IV is based on its comigration with complex IV subunits and Mtc3, Gep7 and Iai11. However, co-migration on complexome profiling does not necessary mean that the proteins interact. The authors should perform pulldowns to confirm the proposed interactions.

> The interaction of these proteins in a complex was previously demonstrated by Morgenstern and collaborators (2017) from the Nils Wiedemann and Bettina Warscheid labs. They immunoprecipitated Aim11 or Iai11 and posterior proteomic analysis revealed

that both copurified with Mtc3 and Gep7, and with each other. A recent bioRxiv manuscript from the Johannes Herrmann lab also confirmed the observation made by Morgenstern et al. In the present manuscript we also confirmed this interaction by immunoprecipitation of Iai11, Mtc3 and Gep7 and western blot, and in all cases Aim11 copurified with all of them. The experiment is included as the new Figure 4A. In addition, we reinforced the Morgenstern findings in the manuscript to make it clearer.

2. The authors used complexome profiling to propose protein interactions. As mentioned under point 1, this approach has its limitation and requires validation by other experimental means. Furthermore, the authors should cite and use the published data from high-resolution complexome profiling of yeast mitochondria (Schulte et al. 2023 Nature) to compare it with their complexome profile.

> The high-resolution complexome profiling of yeast mitochondria published by Schulte *et al* showed similar migration patterns for Gep7/Aim11 and Mtc3/Iai11 as our data. Additional populations for all four proteins were also detected in Schulte *et al* and our dataset, supporting the idea that the four proteins form complexes. We included this analysis in our text (Lines 326-331): *"Similar migration patterns between Aim11 and Gep7, and between Iai11 and Mtc3, were also observed in a previously reported complexome dataset of yeast WT mitochondria (Schulte et al, 2023). Additionally, all four proteins comigrated at around 147 kDa, consistent with the 150 kDa peak where we found the AMIGa complex".*

3. In Figure 3, it is hard to see the COX1 and COX3 modules. The authors should use additional tools like pulldown to confirm the presence f these modules.

> The existence of the three Complex IV modules and their compositions was previously demonstrated by the Tzagoloff group, and therefore, reconfirmation of the presence of the assembly modules is beyond the scope of the present work. We used the previously demonstrated information to identify these modules on the complexome data. Unfortunately, a pulldown will not allow us to differentiate if the protein interacts with modules or with the mature complex. In any case, in this work we focused on how these previously reported modules were modified by the absence of Aim11.

4. Figure 1D; the blue native gels are of poor quality and should be replaced.

> We agree and we repeated the BN gels to improve their quality (see new panel 1D).

5. Figure 4: The authors should show blue native gels for the used deletion mutants to revel their effect for complex IV formation.

> We analyzed mitochondria from all single mutants of the AMIGa complex components by BN-PAGE. In-gel activity for cytochrome *c* oxidase activity is shown on new Figure EV3B. As with the *Δaim11* mutant, deletion of *Δiai11*, *Δgep7* and *Δmtc3* also decreased CIV activity, both as monomeric CIV or in supercomplexes. This essay was discussed on lines 304-306.

6. Figure 1E: Can the authors speculate why the expression of ATP6 is strongly increased in aim11delta?

> Our complexome profiling data showed a slight accumulation of ATP synthase F_1 fraction (New Figure 6I). A higher expression of Atp6 was previously detected by the Tzagoloff group (Rak et al 2009) when free F_1 accumulated. Additionally, a similar phenotype was also observed in null mutant strains of other ATP synthase assembly factors like the INA complex (Naumenko et al 2017). The possible reasons for the increase in *ATP6* mRNA translation are now discussed in lines 437-439 and 514-518.

Referee 2

The manuscript entitled "Aim11 is a novel protein involved in the assembly of mitochondrial cytochrome c oxidase" by Ulrik Pedroza-Dávila, Yolanda Camacho-Villasana, Miriam Vázquez-Acevedo, Madhurya Lutikurti, Diego González-Halphen, Alfredo Cabrera-Orefice and Xochitl Perez-Martinez1 characterizes the role of Aim11 in cytochrome oxidase assembly and provides a valuable workflow for dissecting the refinement steps of mitochondrial biogenesis. The aim11 mutant shows slower respiratory growth and reduced steady-state levels of CIV compared to the wild-type strain. The authors separated mitochondrial protein extracts from the WT and aim11 mutant strains in non-denaturing gel by BN-PAGE, cutting it into over 40 slices and subjecting each to mass spectrometry, an approach that allowed them to identify interactions within CIV and its stable subassemblies. This is a robust, well-designed study that may help elucidate the functions of proteins that participate in, but are not essential for, respiration. This is an important contribution given that about 20% of yeast proteins still lack functional characterization. In addition to confirming previous data from the Tzagoloff lab obtained using other techniques (such as radiolabeling) regarding cytochrome oxidase assembly modules and intermediates, the authors present new findings, including the identification of a role for Aim11 in CIV biogenesis. They show an accumulation of CIV assembly intermediates in the aim11 mutant, demonstrate that Aim11 interacts with the uncharacterized proteins Mtc3, Gep7, and Iai11, all of which contribute together to efficient CIV assembly, and identify the

human protein TMEM242 as the orthologue of Aim11. Overall, the manuscript is original, presenting findings that provide new insights to the field, and it is well written, with a clear structure and coherent presentation of the data.

Major points:

I am surprised that such a drastic reduction in steady-state CIV levels (Figure 1D) results in only a mild respiratory phenotype in the spot test. Do the authors have any idea of what might explain this?

> Cytochrome *c* oxidase activity decreased more than 50% (Figure 1B), yet, as mentioned by the reviewer, the respiratory growth defect does not seem to be so dramatic, especially on ethanol/glycerol medium. In *S. cerevisiae* CIV is not rate limiting of the respiratory chain pathway (see Boumans, H., et al, (1998) Metabolic Control Analysis of the bc_1 complex of Saccharomyces cerevisiae: effect on cytochrome *c* oxidase, respiration, and growth rate. *Biochem. J.* **331:** 877-83.). We propose that the decreased amount of complex IV that is assembled can maintain robust respiratory growth. This is not the first time that we observe this phenomenon; cells carrying a mutation on the mitochondrial Cox1 subunit lost 75% of CIV activity, yet the respiratory growth was robust (García-Villegas R., et al (2017) J. Biol. Chem. 292: 10912-25). We discuss these observations in lines 477-480.

What is the percentage of rho-/rho0 of aim11 mutant in ethanol-glycerol and lactate? The Saccharomyces Genome Database (SGD) reports that the null mutant exhibits increased loss of mitochondrial genome. Is this phenotype in the present study due to unstable mtDNA or to reduced CIV levels? Figure 1E suggests that mtDNA is stable, but the authors should include the percentage of rho^{-}/rho^{0} cells and comment on any differences relative to the phenotype described in SGD.

> In our hands, no change on the rho-/rho0 incidence was detected after deletion of Aim11. This observation is mentioned in line 98-99 .

I find it very interesting that there is an apparent decrease in the Atp9 ring (Figure 1E), given that Atp9 was shown to form an assembly intermediate together with Cox6 that couples the biogenesis of cytochrome oxidase and ATP synthase (Franco et al, 2023). Cox6, in turn, is part of the Cox1 assembly module. Combined with the fact that Aim11 interacts with Cox6, please comment on that.

> We agree that our results strongly suggest that Aim11 has a role on the coordinated assembly of CIV and ATP synthase through Cox6 and the Atp9 ring, which conform the Atco

complex. Indeed, many new experiments that we carried out are focused to develop this idea. In the case of the mitochondrial translation assay from Figure 1E, although at first glance a decrease in Atp9 ring is observed, our densitometric analysis did not show a statistically significant difference between the WT and *AIM11* null mutant. This observation was included in the new panel 1E.

Also, is it possible that the absence of Aim11 is preventing Cox6 from properly interacting with Atp9, thereby causing the accumulation of these Cox1-containing subassemblies (Figure 2B)?

> In new Figure 2F we demonstrated that absence of Aim11 destabilized Cox6, and that over-expression of the *COX6* gene can partially compensate for the respiratory growth defect of a aim11 null mutant. In addition, we detected a physical interaction between Aim11 and the newly synthesized Atp9 c-ring (new Figure 6A). This interaction reflects that Aim11 and therefore the AMIGa complex act on both components of the Atco complex previously proposed by the Tzagoloff group. Maybe Aim11 regulates accessibility of the Atco components for CIV and ATP synthase assembly. When this complex is compromised, Cox6 levels drop, resulting in the accumulation of Cox1-containing intermediates. We discuss this possibility on the Discussion section, lines 536-556 and present a model on new Figure 7.

Minor points:

Please note that some references are repeated:
(Veloso et al, 2018; Su et al, 2014b; Franco et al, 2018; McStay et al, 2013b).
Franco LVR, Su C-H, McStay GP, Yu GJ & Tzagoloff A (2018) Cox2p of yeast cytochrome oxidase assembles as a stand-alone subunit with the Cox1p and Cox3p modules. J Biol Chem 293: 16899-16911
Veloso L, Franco R, Su C-H, Mcstay GP, Yu GJ & Tzagoloff A (2018) Cox2p of yeast cytochrome oxidase assembles as a stand-alone subunit with the Cox1p and Cox3p modules.

> We corrected the references.

"Aim11 is a mitochondrial inner membrane protein of ~15 kDa with two transmembrane helices (Morgenstern et al, 2017)" - There is a period missing after this sentence.
> We corrected it.

"We asked whether the absence of Aim11 affected CIV function or assembly. Thus, we analyzed the steady state levels of CIV subunits (Cox1-3)" - The problem lies in the sentence (not in the experiments themselves). The authors should also mention the BN gel, in which they show that there is indeed much less CIV, and not only lower levels of its subunits.

> We appreciate the suggestion. We have clarified the points by adding the phrase "Next, we asked if the reduction in C*c*O activity is due to a defect on CIV assembly", line 113.

We also added the phrase " Both immunodetection of Cox1 and in-gel CIV activity staining revealed that not only Cox1 and Cox2 levels diminished, but there are also lower levels of monomeric CIV and respiratory super complexes in the *Δaim11* strain", lines 123-126.

In Figure 1A, it would be helpful to show the rich-media growth after just one day.

> Growth of cells on respiratory media is so poor at day one that it is very difficult to observe any difference. However, we replaced Figure 1A (originally showing 3 days of growth) by the 2-days of growth image.

For Figure 1D, it may improve the clarity of the figure if the ATPase in-gel assay is moved from below the Cox1 western. Then, the authors could add the label "ATPase in-gel assay" at the top of the figure, along with the strain names.

> We have corrected this for clarity (Fig 1D).

For Figure 1E, please include (Atp9)n in the "% of WT" graph, as it appears to be decreased.
> We have included Atp9n in the new Figure 1E. As mentioned to Reviewer 1, we did not observe a significant difference on the Atp9 ring levels.

For the Figure 2 legend, please indicate whether the plates correspond to complex or synthetic media (they appear to be synthetic).

> It is indeed synthetic media. Legend of Figure 2 was corrected, along with other figure legends where the information was not included in the previous version of the manuscript.

In Figure 4B, the double mutant displays higher levels of Cox1, Cox2, and Cox3 compared with the single aim11 mutant. What could be the underlying mechanism?

>We analyzed all our biological repeats of this experiment, and we detected that there is no clear difference between the single *Δaim11* mutant and the double mutants. We realized that we did not choose a more representative repetition. Therefore, Figure 4C was updated.

In Figure 4C, it appears that the Δiai11, Δgep7, and Δmtc3 mutants show higher levels of newly translated Cox1 yet reduced steady-state Cox1 levels. Please comment on this.

> We analyzed all our technical and biological repeats of the experiment and increase in Cox1 labeling is not conclusive, that is why we did not make any comment about this observation on the manuscript. In contrast, the increase in Atp6 labeling and the appearance of the small size peptide is consistently observed and discussed on the manuscript.

"TMEM242 from the K562 cell line into a 2 µ yeast expression vector under the ADH promoter and terminator. Then, the plasmid was transformed into different yeast mutants. TMEM242 compensated for the respiratory growth in the Δaim11, Δgep7, Δiai11, and Δmtc3 mutants (Fig 5C)." - It would be important to clarify whether TMEM242 compensates for the Δaim11 phenotype only when overexpressed. Or does it also compensate when expressed from an integrative vector?

> The compensation was only observed when the TMEM242 gene was over-expressed in a 2 µ plasmid with the constitutive *ADH* promoter and terminator. When a low-copy, centromeric plasmid was used no compensation was observed. This observation is presented in new Figure EV4, and mentioned in lines 386-387.

"While Aim11 is the main assembly factor, Iai11, Gep7, and Mtc3 function as accessory factors, since the overexpression of AIM11 complemented the absence of the other three proteins." - The authors should mention the supplementary figure in which they show this result.

> . We have corrected this, now we mentioned Figures 4G and EV3F in lines 505-508.

Referee 3

This manuscript investigates the role of the uncharacterized mitochondrial inner membrane protein Aim11 in the biogenesis of cytochrome c oxidase (Complex IV, CIV) in Saccharomyces cerevisiae. Through growth assays, respiratory chain enzymology, immunoblotting, [35S]-pulse labeling, and especially complexome profiling, the authors conclude that Aim11 participates in the assembly of all three CIV modules (Cox1-, Cox2-, and Cox3-modules). They further show that Aim11 forms a complex with three poorly characterized mitochondrial proteins (Iai11, Gep7, Mtc3) and propose that Aim11 is the "principal" component of an assembly complex. Finally, they present functional cross-species complementation by human TMEM242.

The manuscript addresses an important problem in mitochondrial biology: identifying new components of CIV biogenesis and understanding how modular assembly is integrated. The complexome profiling dataset is extensive and potentially impactful. However, as currently written, the manuscript has significant weaknesses in mechanistic interpretation, experimental rigor, and clarity of presentation, which limit its suitability for EMBO Reports without major revision.

Major Comments

1. Mechanistic insight is insufficiently developed, and the proposed Aim11 roles remain descriptive. The manuscript repeatedly claims that Aim11 "assists progression" of all three CIV modules, yet the mechanistic basis is unclear. The key findings (accumulation of module-specific intermediates; lower Cox1/Cox2 levels; disappearance of Cox6-Aim11 complex; partial rescue by COX6 overexpression) are consistent with defects in late membrane integration and/or stabilization, but do not pinpoint whether Aim11 acts:

(i) as a Cox6 chaperone,
(ii) as a membrane insertase/holdase,
(iii) as a scaffold for module progression.
(iv) as a stability factor for multiple assembly module
(v) or indirectly via inner membrane homeostasis/prohibitins.

The current data allow several interpretations. Additional experiments are needed to clarify the role of Aim11 and its potential effects on all three CIV modules simultaneously.

> We agree that a long way remains to be explored to understand the detailed molecular mechanisms of Aim11 and the AMIGa complex, and we are sure that many more papers on the mechanisms of Aim11 and the AMIGa complex will be published in the years to come.

We expanded the scope of this paper, first by further investigating the effects of Aim11 on stability of Cox6, one of the previously reported interactors of Aim11 and the AMIGa complex. We observed that stability of Cox6 is compromised in the absence of Aim11 and suggested that Aim11 might be involved in controlling availability of Cox6 for Cox1 module assembly. Since Cox6 is part of the Atco complex involved in the coordinated assembly of CIV and ATP synthase, we also investigated if Aim11 had some role on ATP synthase biogenesis. In gel activity of ATP synthase does not seem to be affected by the absence of Aim11, however some F1 subunits accumulated on the mutant. Moreover, we demonstrated that Aim11 physically interacts with newly made Atp9 c-ring. Since both Aim11 interactors, Cox6 and Atp9 c-ring, are components of the Atco complex, we propose that Aim11 has a role regulating assembly of the Atco complex, and therefore, availability of Cox6 and Atp9 subunits for assembly. We prepared a new Figure 2F and Figure 6 describing these results and included a new model for the Aim11 and AMIGa complex role on ATP synthase and CIV biogenesis.

2. Low-quality BN-PAGE data that weaken complexome profile studies. The authors report a large amount of free CIV, even in the WT strain, under their complex extraction conditions, which indicates over-solubilization of the membranes and disruption of the supercomplexes. They used a digitonin:protein ratio of 3:1, but it is not indicated whether they used high-purity digitonin. Impurities are well known to disrupt membranes in a rather uncontrolled manner. In addition to using high-purity digitonin, I suggest including additional bona fide CIV mutants as controls, as the membrane solubilization conditions may be too harsh, and some peripheral subunits (such as Cox12) may be ripped from the complex.

> The BN-PAGE gels used for Figure 1, EV3B and in-gel activity staining have been replaced with higher-quality gels as requested. Importantly, these gels and the ones used for complexome profiling were performed following a similar protocol but with different chemicals, by different individuals, and in different laboratories. The complexome data were collected in the laboratories of Prof. Ulrich Brandt (Nijmegen, NL) and Dr. Ilka Wittig

(Frankfurt, Germany), two leading groups in complexome profiling, following the widely accepted Nature protocols of Wittig et al., 2006.

Regarding digitonin purity, we used high-purity water-soluble digitonin from SERVA (Heidelberg, Germany). Having worked with multiple batches of this product for over a decade, we have observed negligible batch-to-batch variation, giving us confidence that impurities are not significantly affecting our preparations.

Concerning the amount of free CIV observed in the WT strain, we respectfully note that in *S. cerevisiae*, the ratio of free CIV to supercomplex-associated CIV is not fixed, but varies with strain background, growth conditions and media composition. In our dataset, the majority of CIV is associated with supercomplexes, consistent with expectations. The presence of some free CIV therefore does not necessarily indicate over-solubilization.

Regarding Cox12, its low signal from CIV-containing fractions is a well-documented observation not specific to our conditions. Furthermore, its small size and hydrophobic nature make it prone to escape MS identification. There is still visible signal of Cox12 comigrating with the CIV-containing fractions. We have included the relevant reference. This subunit seems to be sensitive to Coomassie blue dye, as we have observed markedly better stabilization of the Cox12 interaction with CIV in high-resolution Clear Native electrophoresis (private unpublished data of an ongoing collaboration). The remaining CIV subunits are detected at comparable stoichiometric levels, which is why we do not consider the addition of bona fide CIV mutant controls necessary to clarify the scope of this manuscript.

3. Complexome profiling interpretation needs more rigor and quantitation. Complexome analysis is a major strength, but the interpretation is partly qualitative. Specific issues include:
3.1. Quantitation is lacking. For example, "~70% decrease" in supercomplexes requires numerical data, error bars, and statistical testing. Area-under-the-curve quantifications must be shown and methods described in detail

> Complexome profiling was applied here primarily to identify protein interactions and detect global changes in complex assembly, rather than for strict quantitative comparison. Each condition was analysed in at least two independent replicates, thus not allowing classical statistical testing. Given the technical demands of this approach, where each gel slice requires approximately 50–60 minutes of MS instrument time, running additional replicates would only be justified if the primary goal were quantitative rather than qualitative. Nevertheless, to address this concern, we have now provided area-under-the-

curve (AUC) values for the compared fractions, allowing more precise numerical assessment of the differences observed between conditions The complexome profiling methods section has been expanded accordingly.

3.2. Subcomplex assignments are overconfident. Most "subassemblies" are assigned based solely on comigration, though large shifts suggest missing partners. Without immunoprecipitation or crosslinking evidence, the manuscript should avoid definitive statements about composition. Orthogonal approaches are absolutely needed.

> We agree that comigration on BN-PAGE alone is not sufficient to definitively establish complex composition. However, our subcomplex assignments were not based solely on comigration patterns. First, we cross-referenced our observations with previously published literature on the reported compositions of CIV assembly modules, identifying which subunits and chaperones were expected to co-migrate at specific molecular masses. Second, we used iBAQ values as an additional criterion to evaluate the plausibility of proposed interactions; proteins present at vastly different abundances (for example, one at 1–3 million and another at 20–30 million iBAQ units) were not considered likely interaction partners despite sharing similar migration patterns, since proteins within a complex are expected to show comparable absolute abundances when sufficient peptides are detected. Third, we now performed some co-immunoprecipitation and western blot analyses as orthogonal support for key interactions (see new Figures 2F, 4A, 6A).

We have addressed this concern and toned down our subcomplex assignments throughout the manuscript, framing comigration data as indicative rather than definitive where orthogonal validation is lacking. Additionally, during the preparation of this revision, Kizmaz et al. (2026) independently validated the AMIGa complex composition and the Aim11-Cox6 interaction by IP-MS. Together with the original Morgenstern et al. (2017) pulldown data, we believe the current level of evidence is appropriate for the claims made in the revised manuscript.

3.3. The essential distinction between assembly intermediates and degradation fragments is not addressed

Cox1 and Cox3 low-mass species could represent proteolysis fragments (common when assembly stalls). This must be considered.

> We thank the reviewer for raising this point. While we acknowledge that low-mass species could in principle represent degradation fragments, several observations support their identity as assembly intermediates. The detected species contain multiple co-migrating

subunits and chaperones at stoichiometrically comparable abundances, which is inconsistent with random proteolytic fragments. Furthermore, their observed molecular masses correlate well with the theoretical masses of the proposed subunit compositions and several of these intermediates have been previously described in the literature. Finally, their specific enrichment in the *Δaim11* mutant rather than appearing as a diffuse low-mass signal argues against non-specific proteolysis. Nonetheless, we have moderated our claims in the revised manuscript where this distinction cannot be unambiguously established.

3.4. The claim that Aim11 affects all three modules equally is not quantitatively supported Cox1 appears most severely affected; Cox3 module effects seem secondary.

> We corrected this asseveration in line 261-263.

4. The interpretation of [35S]-labeling experiments is problematic. The authors conclude that mitochondrial translation is not impaired. However, (i) Cox1 synthesis appears unchanged (bypassing CIV-dependent COX1 mRNA translation regulation), yet steady-state Cox1 is drastically lower, suggesting rapid turnover. (ii) Cox2 and Cox3 synthesis increase, which is a hallmark of imbalanced module assembly. (iii) Atp6 synthesis dramatically increases, but ATP synthase levels do not, suggesting broader perturbation of membrane protein homeostasis.
These data strongly suggest an inner membrane protein quality-control defect rather than selective CIV assembly failure. This must be addressed explicitly.

> To our interpretation, translation was not impaired since labeling of any of the mt-DNA translation products decreased. However, we agree that perturbations in protein synthesis need a more detailed description. These observations are now discussed in lines to with you that assembly is affected in the null mutant as expressed in lines 135-141.

5. A critical experiment is missing: Cox6 stability and turnover. According to the data presented, Cox6 is the most compelling Aim11-related target. Cox6 steady-state levels are reduced; Aim11-Cox6 comigrate in complexome data; COX6 overexpression partially rescues growth; and Gep7 and Mtc3 share structural similarity with Cox5/Cox4 family proteins. However, Cox6 stability or half-life is never measured. Cycloheximide chase or pulse-chase assays for Cox6 are essential to clarify the model.

> We determined the steady-state levels of Cox6 in the absence of Aim11 and observed that, indeed, Cox6 levels were dramatically reduced, indicating that stability/turnover of Cox6 is

compromised if Aim11 is missing. We added panel F to Figure 2 showing this result and discussed this observation in the related text of the manuscript.

6. The link between Aim11 and prohibitins is underdeveloped. Aim11 is synthetic lethal with prohibitins, but the manuscript does not integrate this observation. Prohibitins shape inner membrane domains and regulate the insertion of Cox1/Cox3, as shown in several papers. Aim11 may act in the same membrane quality-control network. The discussion should address this explicitly.

> We agree that specific studies are needed to focus on the relationship between prohibitins and the AMIGa complex. However, the aim of the present manuscript was to describe the possible roles of Aim11 and the AMIGa complex on CIV and ATP synthase biogenesis. However, our complexome dataset provided us with interesting information that we added as the new Figure EV1A. In the absence of Aim11 the Phb1 and Phb2 prohibitins subunits accumulated, reinforcing the genetic relation between Aim11 and prohibitins. At the same time, we established a role of Aim11 in the assembly of the ATP synthase, including the chaperone Atp10 and the protease Atp23. Interestingly, deletion of these genes is also synthetic lethal in the absence of prohibitins.

We added a discussion regarding this point in the Discussion section, where we propose that there might be a compensatory increase of the prohibitin in the absence of Aim11.

It is worth to mention that few days after our preprint publication the Hermann lab published a preprint studying the possible role of Aim11 in the channel insertion machinery, that could also explain the prohibitin interaction data, reinforcing the idea that much more attention is needed in future papers to understand how the prohibitins and AMIGa complex act together. All these points were added to the discussion (lines 560-578).

7. The low-molecular-mass polypeptide observed in all mutants is never identified. This is repeatedly mentioned as "unknown". Is it a proteolytic product? An unprocessed mitochondrial protein? A marker of membrane stress? At a minimum, the authors need to attempt identification (LC-MS/MS) or provide a reasonable hypothesis.

> We agree that this "unknown" low molecular weight product is very interesting and revealing its identity could shed light on the mechanims of action of the AMIGa complex. Unfortunately, the possibility of identifying this peptide by LC-MS/MS is close to zero. This autoradiography comes from an SDS-PAGE experiment where many other proteins migrate

at the same size, and if the peptide is an aberrant sequence, then the database will not identify it. We proposed two hypotheses. First, the peptide could be derived from the *ATP6* mRNA since *ATP6* mRNA translation is highly increased in absence of Aim11. The second is if Aim11 binds Atp9, the unknown peptide would correspond to the degradation of Atp9 in the null mutant. We discuss these possibilities in line 142-143 and 551-553.

8. The authors refer to the Aim11-Mtc3-Gep7-Iai11 complex as a demonstrated fact, but the only experimental evidence presented is co-sedimentation. While for subunits already known to belong to the same enzyme, it may be sufficient to moderate the conclusion statements, for this new "complex," an IP assay is absolutely necessary. Also, are the other components interacting in the absence of Aim11?

The association of these proteins was previously demonstrated by immunoprecipitation and MS identification by Nils Wiedemann and Bettina Warscheid labs in 2017 (Morgesten 2017). We repeated our own immunoprecipitations and western blot experiments and included them in the new Figure 4.

9. The study does not explain why Aim11 affects CIII2 activity. Complex III2 activity decreases to 70% of wild-type. The authors attribute this to CIV-related defects, but this is rather speculative. In the literature, some CIV assembly mutants have increased rather than decreased CIII activity. Again, the discussion must address this point and how it fits into the current model, and consider whether the model should be broadened to include all observations.

> It is true that even though CIV is the most sensitive to the absence of Aim11, other OXPHOS complexes are also altered, we acknowledge these observations more emphatically in the Discussion section also lines 557-574.

Minor Comments

1. Discussion of Cox12 dissociation during BN-PAGE should more clearly reference the literature.

> We thank the reviewer for this suggestion. The dissociation of Cox12 from CIV under BN-PAGE conditions has been previously reported by Strecker et al. (2016), who described Cox12/Cox26 as a peripheral supercomplex-associated subunit prone to dissociation

during blue native electrophoresis (see answer for reviewer 2). We have now cited this reference explicitly in the relevant section of the revised manuscript.

2. The paper should clearly distinguish "association" vs. "true physical interaction", especially for complexome-derived inferences.

> While we consider association and physical interaction to be equivalent terms in our view, we understand the concern regarding the strength of evidence behind each claim. In the revised manuscript, we have been more careful to distinguish between direct and indirect interactions, and to clearly indicate where conclusions are inferred from complexome data versus orthogonal biochemical approaches.

5. BN-PAGE solubilization conditions (digitonin purity) must be reported.

> We have reported this in the respective section: research grade water-soluble digitonin (SERVA GmbH)

6. The normalization strategy for the complexome profiling dataset is not described.

> We have expanded the methods section clearly indicating how the normalization was performed.

7. Details of statistical analyses are lacking.

> For the enzyme activities, pairwise student's t-test were performed to determine P-values. For complexome profiling no statistical analyses were performed.

8. As a suggestion, the authors may consider reorganizing the figures to emphasize a clear assembly model rather than exhaustive cataloguing of intermediates.

> We included new figures and changed the order of presentation of some main and expanded view figures to try to direct the audience to a clearer assembly model. Also, the addition of a Discussion section provides us with more space to discuss in depth our current model (new Figure 7).

Dear Dr. Pérez-Martínez

Thank you for the submission of your revised manuscript to EMBO reports. We have now received the full set of referee reports that is copied below.

As you will see, all referees are positive about the study and request only minor changes.

From the editorial side, there are also a few things that we need before we can proceed:

- As a standard procedure, we edit the abstract of manuscripts to make them more accessible to a general readership. Please find the suggested version below my signature, which has been edited to present tense.

- Please rename the "Materials and Methods" section to "Methods".

- Please place the manuscript sections in the following order: Title page - Abstract - Introduction - Results - Discussion - Methods - Data Availability - Acknowledgments - Disclosure Statement & Competing Interests - References - Figure Legends - (Main Tables with legends if applicable) - Expanded View Figure Legends.

- The abbreviations section needs to be removed from the manuscript. Abbreviations should be defined in brackets after their first mention in the text, not in a list of abbreviations.

- Please note that the data callouts in the text for PXD006147- data citation does not include "Data ref:" as a prefix.

- CRediT has replaced the traditional author contributions section because it offers a systematic machine readable author contributions format that allows for more effective research assessment. Please remove the Author Contributions section from the manuscript and use the free text boxes beneath each contributing author's name in our online systems to add specific details on the author's contribution. More information is available in our guide to authors.

- Nomenclature of EV figures in the figure legends needs correction to Figure EV1, Figure EV2, etc. (Expanded View 1, Expanded View 2, etc. is not correct).

- Please adjust the in-text callouts for individual figures and figure panels: e.g. EV5 appears to be missing.

- Please double-check to make sure to all relevant funding information in the manuscript is congruent with the info entered into our submission system (SECIHTI (CVU 883299), IF262026 need to be entered in eJP).

- Please upload the EV tables as individual files. Please correct the nomenclatures of the EV tables in all places (source file names, titles in the system, title of the legends, ms callouts) to Table EV1, etc.

- The entry for short DNA sequences in the Author Checklist is empty, but seems that it should point to an EV table.

- We are unable to access the source data uploaded to BioStudies. To enable source data checks, please provide the source data not associated with omics/large datasets either by uploading to our system with your next submission or fixing the BioStudies link.

- It appears that the entries for Figures 1B and C are switched in the source data checklist. Also, Figure EV5 appears to be a blot image, which is not reflected in the source data checklist.

- Please include a statement on blinding in statistical analysis, even if no blinding was performed.

- EMBO Reports requires figures with N<5 to display individual data points. Please doublecheck your figures to ensure this is followed, particularly Figure 1B.

- Our production/data editors have asked you to clarify several points in the figure legends (see below). Please incorporate these changes in the manuscript and return the revised file with tracked changes with your final manuscript submission.
- Please note that the legends for figure EV-2 are not provided in the sequential manner (legend for figure EV-2G is provided before legend of figure EV-2F). This needs to be rectified.
- Please note that the legend for figures EV-4G,H are missing in the manuscript. This needs to be rectified.
- Please note that the sub-figure EV-3H is not labeled in the figure panel in the manuscript. This needs to be rectified.
- Please indicate the statistical test used for data analysis in the legends of figures 1B,E.
- Please note that information related to n is missing in the legends 1E.
- Please note that the error bars are not defined in the legends of figures 1B,E.

- Please note that EMBO press papers are accompanied online by:
A) a short (2 sentences) summary of the findings and their significance,
B) 2-5 short bullet points highlighting the key results, and
C) a synopsis image in .jpg or .png format that is exactly 550 pixels wide and 300 pixels high. Please note that the text needs to be legible at the final size. Please upload this information along with your revised manuscript (the text for A and B should be provided in one separate Word file uploaded as Cover Art/Synopsis Image).

We look forward to your final revised manuscript.

Kurt Weir
Editor
EMBO Reports

Cytochrome c oxidase (CIV) and ATP synthase are part of the oxidative phosphorylation machinery. Their modular, highly regulated assembly requires specific factors and is coordinated through a common intermediate, the Atco complex, composed of the Atp9 ring and Cox6. By high-throughput analysis, it was previously detected that Aim11 interacts with some CIV and ATP synthase subunits, but its relevance was unknown. In this work, we characterize the role of Aim11 in CIV and ATP synthase biogenesis. We find that the null mutant exhibits reduced respiratory growth and diminished CIV activity. Mitochondrial complexome profiling of the Δaim11 mutant shows an accumulation of intermediates of the CIV and ATP synthase assembly modules. Aim11 forms a complex with three other proteins: Mtc3, Gep7, and Iai11, which we coin as AMIGa (Aim11-Mtc3-Iai11-Gep7 assembly complex). Aim11 interacts with the newly synthesized Atp9 ring, possibly mediated by the Atco complex. In addition, we identify the human protein TMEM242 as an Aim11 orthologue.

Referee #1:

The revised version of the manuscript improved. Many of my concerns have been addressed. The topic is interesting and suited for publication in EMBO rep. However, I have some minor concerns that should be addressed:

1. The authors should re-do the blue native gels in Fig. 1D. The respiratory chain supercomplexes are not resolved. Furthermore, complex IV monomer appears to be strongly reduced, which is not the case in the complexome profiling. Complex III should be shown.

2. The authors added new data on a possible role of Aim11 in the biogenesis of the ATP-synthase and propose an interaction of Aim11 with Atco. This part appears in part preliminary and the link to complex IV is not clear. For instance, does Aim11 bind to Cox6? Are there any biogenesis defects of the ATP synthase?

Referee #2:

The manuscript is of high quality. The authors have thoroughly addressed the reviewers' comments and concerns, substantially improving the manuscript. I recommend its publication.

Minor points:

Line 350-353: As observed for the Δaim11 mutant, overexpression of COX6 partially recovered the respiratory phenotype of single Δgep7, Δiai11 or Δmtc3 mutants, of the triple Δgep7/Δiai11/Δmtc3 and of the quadruple Δaim11/Δgep7/Δiai11/Δmtc3 mutants (Fig 4G, EV3G). - There is nothing on COX6 in Fig 4G.

Line 1157: (A) Serial dilutions of Δaim11 cells with the indicated overexpression plasmids - I believe this does not correspond to overexpression.

Line 538: Morgenstern et al, 2017a). and the Atp9 c-ring. - remove extra period

Regarding undefined band in Fig 1E: At line 141, the band is suggested to be related to Atp6, while at line 551 it is suggested to be related to Atp9.

Referee #3:

I appreciate the substantial effort the authors have invested in revising this manuscript. The revised version is considerably stronger than the original submission, both experimentally and conceptually. The authors have responded carefully to the

reviewers' comments and have expanded the scope of the study with a significant amount of new data. In particular, the validation of the AMIGa complex by immunoprecipitation, the additional analyses of Cox6 stability, the rescue experiments by COX6 overexpression, the characterization of ATP synthase assembly defects, the demonstration of Aim11 interaction with the newly synthesized Atp9 c-ring, the genetic interaction analyses, and the incorporation of TMEM242 into the proposed model substantially strengthen the manuscript. Altogether, these new experiments transform the study from the description of a new complex IV assembly factor into a broader and more interesting investigation of a protein complex that appears to coordinate cytochrome c oxidase and ATP synthase biogenesis through the Atco pathway.
Overall, I believe that the major concerns raised during the first round of review have been satisfactorily addressed. The manuscript now presents a coherent and compelling body of evidence supporting a role for the AMIGa complex in mitochondrial respiratory complex biogenesis. Although the precise molecular mechanism remains to be elucidated, I do not consider this a prerequisite for publication, as the current study establishes an important conceptual framework that will undoubtedly stimulate future mechanistic work.
I have only a few remaining comments, which are primarily related to the interpretation of the data rather than to the experimental work itself.
1. In several places throughout the manuscript the mechanistic conclusions are stated more strongly than warranted by the available evidence. The data convincingly demonstrate that Aim11 and the AMIGa complex influence the assembly of both cytochrome c oxidase and ATP synthase and that they interact with components of the Atco intermediate. However, statements implying that Aim11 directly regulates Atco assembly, stabilizes Cox6, or controls the progression of specific assembly modules should be phrased more cautiously. The current data are highly consistent with these models but do not yet establish direct molecular mechanisms. Replacing expressions such as "Aim11 regulates" or "Aim11 stabilizes" with wording such as "the data suggest," "are consistent with," or "support a model in which" would better reflect the strength of the evidence.
2. While the existence of the AMIGa complex is now well supported, its structural organization remains unresolved. The proposed Aim11-Gep7 and Iai11-Mtc3 heterodimers represent plausible interpretations of the complexome data, but these arrangements remain hypothetical and should be presented as such.
3. Most of the conclusions regarding ATP synthase biogenesis are necessarily based on indirect evidence, including Atp9 interaction, Atp25 and INA complex accumulation, and altered Atp6 translation. These observations strongly support an involvement of the AMIGa complex in ATP synthase biogenesis, but they do not yet define its precise biochemical function. I therefore encourage the authors to maintain a clear distinction throughout the manuscript between experimentally demonstrated findings and the mechanistic model proposed in the Discussion.

Referee Cross-comment:

Referee 3
All reviewers seem to agree that the manuscript is significantly improved and that all major concerns have been addressed. There are a few minor points to be corrected.

Answer to comments:

EMBOR-2025-63194V2 Decision Letter

Dear Dr. Pérez-Martínez

Thank you for the submission of your revised manuscript to EMBO reports. We have now received the full set of referee reports that is copied below.

As you will see, all referees are positive about the study and request only minor changes.

From the editorial side, there are also a few things that we need before we can proceed:

- As a standard procedure, we edit the abstract of manuscripts to make them more accessible to a general readership. Please find the suggested version below my signature, which has been edited to present tense.

> *We incorporated the suggested abstract.*

- Please rename the "Materials and Methods" section to "Methods".

> *Done*

- Please place the manuscript sections in the following order: Title page - Abstract - Introduction - Results - Discussion - Methods - Data Availability - Acknowledgments - Disclosure Statement & Competing Interests - References - Figure Legends - (Main Tables with legends if applicable) - Expanded View Figure Legends.

> *We have followed instructions.*

- The abbreviations section needs to be removed from the manuscript. Abbreviations should be defined in brackets after their first mention in the text, not in a list of abbreviations.

> *We don't have an Abbreviations section. In any case, we took care of defining the first mention of a term in brackets.*

- Please note that the data callouts in the text for PXD006147- data citation does not include "Data ref:" as a prefix.

> *This was corrected.*

- CRediT has replaced the traditional author contributions section because it offers a systematic machine readable author contributions format that allows for more effective research assessment. Please remove the Author Contributions section from the manuscript and use the free text boxes beneath each contributing author's name in our online systems to add specific details on the author's contribution. More information is available in our guide to authors.

> *The "Author contributions section was removed from the manuscript and used CRediT instead.*

- Nomenclature of EV figures in the figure legends needs correction to Figure EV1, Figure EV2, etc. (Expanded View 1, Expanded View 2, etc. is not correct).

> *We changed nomenclature to Figure EV1, etc.*

- Please adjust the in-text callouts for individual figures and figure panels: e.g. EV5 appears to be missing.

> *We checked the in-texts callouts for individual figures. In particular, Figure EV5 is cited in the Methods section-Complexome profiling.*

- Please double-check to make sure to all relevant funding information in the manuscript is congruent with the info entered into our submission system (SECIHTI (CVU 883299), IF262026 need to be entered in eJP).

> *We corrected this for all funding sources.*

- Please upload the EV tables as individual files. Please correct the nomenclatures of the EV tables in all places (source file names, titles in the system, title of the legends, ms callouts) to Table EV1, etc.

> *Done*

- The entry for short DNA sequences in the Author Checklist is empty but seems that it should point to an EV table.

> *Done*

- We are unable to access the source data uploaded to BioStudies. To enable source data checks, please provide the source data not associated with omics/large datasets either by uploading to our system with your next submission or fixing the BioStudies link.

> The BioStudies database informed me that the data is now available. The new link is indicated in the Data Source Checklist file. The link is:

https://www.ebi.ac.uk/biostudies/studies/S-BSST3189?query=S-BSST3189

- It appears that the entries for Figures 1B and C are switched in the source data checklist. Also, Figure EV5 appears to be a blot image, which is not reflected in the source data checklist.

> Regarding Figure EV5, we changed "macroscopic photo" to "gel image" on the source data checklist. In addition, entries for Fig. 1B and C were corrected in the source data checklist.

- Please include a statement on blinding in statistical analysis, even if no blinding was performed.

> The phrase "The statistical analysis was unblinded" was included on the Methods section, line 735.

- EMBO Reports requires figures with N<5 to display individual data points. Please doublecheck your figures to ensure this is followed, particularly Figure 1B.

> Figure 1B has N=9, so we did not show the individual data points.

- Our production/data editors have asked you to clarify several points in the figure legends (see below). Please incorporate these changes in the manuscript and return the revised file with tracked changes with your final manuscript submission.

- Please note that the legends for figure EV-2 are not provided in the sequential manner (legend for figure EV-2G is provided before legend of figure EV-2F). This needs to be rectified.

> It was corrected.

- Please note that the legend for figures EV-4G,H are missing in the manuscript. This needs to be rectified.

> It was corrected

- Please note that the sub-figure EV-3H is not labeled in the figure panel in the manuscript. This needs to be rectified.

> The manuscript has no Figure EV3H. In any case we doubled check this fact.

- Please indicate the statistical test used for data analysis in the legends of figures 1B,E.

> Done

- Please note that information related to n is missing in the legends 1E.

> *Corrected*

- Please note that the error bars are not defined in the legends of figures 1B,E.

> *Corrected*

- Please note that EMBO press papers are accompanied online by:
A) a short (2 sentences) summary of the findings and their significance,
B) 2-5 short bullet points highlighting the key results, and
C) a synopsis image in .jpg or .png format that is exactly 550 pixels wide and 300 pixels high. Please note that the text needs to be legible at the final size. Please upload this information along with your revised manuscript (the text for A and B should be provided in one separate Word file uploaded as Cover Art/Synopsis Image).

> *We included these files as indicated.*

We look forward to your final revised manuscript.

Kurt Weir
Editor
EMBO Reports

Cytochrome c oxidase (CIV) and ATP synthase are part of the oxidative phosphorylation machinery. Their modular, highly regulated assembly requires specific factors and is coordinated through a common intermediate, the Atco complex, composed of the Atp9 ring and Cox6. By high-throughput analysis, it was previously detected that Aim11 interacts with some CIV and ATP synthase subunits, but its relevance was unknown. In this work, we characterize the role of Aim11 in CIV and ATP synthase biogenesis. We find that the null mutant exhibits reduced respiratory growth and diminished CIV activity. Mitochondrial complexome profiling of the Δaim11 mutant shows an accumulation of intermediates of the CIV and ATP synthase assembly modules. Aim11 forms a complex with three other proteins: Mtc3, Gep7, and Iai11, which we coin as AMIGa (Aim11-Mtc3-Iai11-Gep7 assembly complex). Aim11 interacts with the newly synthesized Atp9 ring, possibly mediated by the Atco complex. In addition, we identify the human protein TMEM242 as an Aim11 orthologue.

Referee #1:

The revised version of the manuscript improved. Many of my concerns have been addressed. The topic is interesting and suited for publication in EMBO rep. However, I have some minor concerns that should be addressed:

1. The authors should re-do the blue native gels in Fig. 1D. The respiratory chain supercomplexes are not resolved. Furthermore, complex IV monomer appears to be

strongly reduced, which is not the case in the complexome profiling. Complex III should be shown.

> It is true that the supercomplexes are not completely resolved in Fig. 1D, even though we repeated the experiment as previously requested. However, in the Blue Native gel used for complexome profiling, supercomplexes are also poorly resolved. Regarding differences in free CIV monomer abundance, the BN gels for activity/western blot and complexome profiling were performed in two different labs, so that conditions may have varied slightly; BN-PAGE is quite sensitive to those details. However, both BN-PAGE experiments corroborate the main results: cytochrome c oxidase is reduced, while ATP synthase does not seem as affected. The main contribution of Fig. 1D is to show that CIV activity is reduced and that complex V activity is similar in WT and Daim11 mitochondria. The main contribution of complexome profiling is to identify subassembly complexes as well as assembled CIV and ATP synthase more clearly. Finally, we decided not to show Complex III in Figure 1D so the audience could focus on the main observation that Aim11 connects CIV and ATP synthase assembly.

2. The authors added new data on a possible role of Aim11 in the biogenesis of the ATP-synthase and propose an interaction of Aim11 with Atco. This part appears in part preliminary and the link to complex IV is not clear. For instance, does Aim11 bind to Cox6? Are there any biogenesis defects of the ATP synthase?

> The biogenesis defects on ATP synthase seem to be minor in terms of the enzyme activity; however, we reported the following observations: Translation of the ATP6 mRNA increased (Fig. 1E), the Atp9 ring and other Atp9 intermediates accumulate in the Daim11 mutant (Fig. 6C), Atp25, Ina17 and Ina22 intermediates accumulate after Aim11 deletion. The same is observed for Atp6, Atp10 and Atp23 (Fig. 6D-H). Regarding association of Cox6 and Aim11, we do observe a comigration of the AMIGa complex and Cox6 in our complexome profiling. After Aim11 deletion, migration of Cox6 switched (Figure EV3D), supporting the physical interaction between Aim11 and Cox6. In fact, the Aim11-Cox6 interaction was previously reported by Morgenstern (2017), and our results agree with this observation.

Referee #2:

The manuscript is of high quality. The authors have thoroughly addressed the reviewers' comments and concerns, substantially improving the manuscript. I recommend its publication.

Minor points:

Line 350-353: As observed for the Δaim11 mutant, overexpression of COX6 partially

recovered the respiratory phenotype of single Δgep7, Δiai11 or Δmtc3 mutants, of the triple Δgep7/Δiai11/Δmtc3 and of the quadruple Δaim11/Δgep7/Δiai11/Δmtc3 mutants (Fig 4G, EV3G). - There is nothing on COX6 in Fig 4G.

> The COX6 overexpression experiments are shown in Fig. EV3G. We corrected the text in line 350-353.

Line 1157: (A) Serial dilutions of Δaim11 cells with the indicated overexpression plasmids - I believe this does not correspond to overexpression.

> Correct, overexpression of TMEM242 is shown in Fig. 5D. In contrast, Fig. EV4 is showing TMEM242 expression on a centromeric plasmid and under the control of the MSS51 promoter and terminator sequences, and thereof is not overexpressed. We corrected this observation in Figure Legend EV4A. Thank you for pointing out this mistake.

Line 538: Morgenstern et al, 2017a). and the Atp9 c-ring. - remove extra period

> Done

Regarding undefined band in Fig 1E: At line 141, the band is suggested to be related to Atp6, while at line 551 it is suggested to be related to Atp9.

> Thank you for noticing this, we propose in the discussion section (line 551) that it is possibly related to Atp9, and that this peptide could be the result of a degradation product after the AMIGa complex deletion. We corrected this on line 141 by changing the phrase to: " This peptide could originate from Atp9 (see Discussion)."

Referee #3:

I appreciate the substantial effort the authors have invested in revising this manuscript. The revised version is considerably stronger than the original submission, both experimentally and conceptually. The authors have responded carefully to the reviewers' comments and have expanded the scope of the study with a significant amount of new data. In particular, the validation of the AMIGa complex by immunoprecipitation, the additional analyses of Cox6 stability, the rescue experiments by COX6 overexpression, the characterization of ATP synthase assembly defects, the demonstration of Aim11 interaction with the newly synthesized Atp9 c-ring, the genetic interaction analyses, and the incorporation of TMEM242 into the proposed model substantially strengthen the manuscript. Altogether, these new experiments transform the study from the description of a new complex IV assembly factor into a broader and more interesting investigation of a protein complex that appears to coordinate cytochrome c oxidase and ATP synthase biogenesis through the Atco pathway.

Overall, I believe that the major concerns raised during the first round of review have been satisfactorily addressed. The manuscript now presents a coherent and compelling body of evidence supporting a role for the AMIGa complex in mitochondrial respiratory complex biogenesis. Although the precise molecular mechanism remains to be elucidated, I do not consider this a prerequisite for publication, as the current study establishes an important conceptual framework that will undoubtedly stimulate future mechanistic work.
I have only a few remaining comments, which are primarily related to the interpretation of the data rather than to the experimental work itself.

1. In several places throughout the manuscript the mechanistic conclusions are stated more strongly than warranted by the available evidence. The data convincingly demonstrate that Aim11 and the AMIGa complex influence the assembly of both cytochrome c oxidase and ATP synthase and that they interact with components of the Atco intermediate. However, statements implying that Aim11 directly regulates Atco assembly, stabilizes Cox6, or controls the progression of specific assembly modules should be phrased more cautiously. The current data are highly consistent with these models but do not yet establish direct molecular mechanisms. Replacing expressions such as "Aim11 regulates" or "Aim11 stabilizes" with wording such as "the data suggest," "are consistent with," or "support a model in which" would better reflect the strength of the evidence.

> *We modified the phrasing of our conclusions throughout the manuscript as suggested.*

2. While the existence of the AMIGa complex is now well supported, its structural organization remains unresolved. The proposed Aim11-Gep7 and Iai11-Mtc3 heterodimers represent plausible interpretations of the complexome data, but these arrangements remain hypothetical and should be presented as such.

> *We changed the phrasing on line 346 according to the reviewer's suggestion.*

3. Most of the conclusions regarding ATP synthase biogenesis are necessarily based on indirect evidence, including Atp9 interaction, Atp25 and INA complex accumulation, and altered Atp6 translation. These observations strongly support an involvement of the AMIGa complex in ATP synthase biogenesis, but they do not yet define its precise biochemical function. I therefore encourage the authors to maintain a clear distinction throughout the manuscript between experimentally demonstrated findings and the mechanistic model proposed in the Discussion.

> *We included the phrase " Our observations strongly support the involvement of the AMIGa complex in ATP synthase biogenesis, but the precise molecular mechanism remains unknown. " in line 555.*

Referee Cross-comment:

Referee 3
All reviewers seem to agree that the manuscript is significantly improved and that all major concerns have been addressed. There are a few minor points to be corrected.

Dr. Xochitl Pérez-Martínez
Instituto de Fisiología Celular, Universidad Nacional Autónoma de México
Genética Molecular
Ciudad Universitaria
CDMX 04510
Mexico

Dear Dr. Pérez-Martínez,

I am very pleased to accept your manuscript for publication in the next available issue of EMBO reports. Thank you for your contribution to our journal.

Your manuscript will be processed for publication by EMBO Press. It will be copy edited and you will receive page proofs prior to publication. Please note that you will be contacted by Springer Nature Author Services to complete licensing and payment information.

You may qualify for financial assistance for your publication charges - either via a Springer Nature fully open access agreement or an EMBO initiative. Check your eligibility: https://link.springer.com/journal/44319/how-to-publish-with-us

Should you be planning a Press Release on your article, please notify the production team via this form in order to coordinate publication and release dates: https://forms.cloud.microsoft/e/eH4A8JVP6g

If you have any questions, please do not hesitate to contact the Editorial Office. Thank you for your contribution to EMBO Reports.

Yours sincerely,

Martina Rembold, PhD
Senior Editor
EMBO Reports

>>> Please note that it is EMBO Reports policy for the transcript of the editorial process (containing referee reports and your response letter) to be published as an online supplement to each paper. If you do NOT want this, you will need to inform the Editorial Office via email immediately. More information is available here: https://link.springer.com/partners/embo-press/editorial-policies#Peer%20review

EMBO Press Author Checklist

Corresponding Author Name: Xochitl Perez-Martinez	
Journal Submitted to: EMBO Reports	
Manuscript Number: EMBOR-2025-63194	

USEFUL LINKS FOR COMPLETING THIS FORM
The EMBO Journal - Author Guidelines
EMBO Reports - Author Guidelines
Molecular Systems Biology - Author Guidelines
EMBO Molecular Medicine - Author Guidelines

Reporting Checklist for Life Science Articles (updated January
This checklist is adapted from Materials Design Analysis Reporting (MDAR) Checklist for Authors. MDAR establishes a minimum set of requirements in transparent reporting in the life sciences (see Statement of Task: 10.31222/osf.io/9sm4x). Please follow the journal's guidelines in preparing your
Please note that a copy of this checklist will be published alongside your article.

Abridged guidelines for figures
1. Data
The data shown in figures should satisfy the following conditions:
- the data were obtained and processed according to the field's best practice and are presented to reflect the results of the experiments in an accurate and unbiased manner.
- ideally, figure panels should include only measurements that are directly comparable to each other and obtained with the same assay.
- plots include clearly labeled error bars for independent experiments and sample sizes. Unless justified, error bars should not be shown for technical
- if n<5, the individual data points from each experiment should be plotted. Any statistical test employed should be justified.
- Source Data should be included to report the data underlying figures according to the guidelines set out in the authorship guidelines on Data

2. Captions
Each figure caption should contain the following information, for each panel where they are relevant:
- a specification of the experimental system investigated (eg cell line, species name).
- the assay(s) and method(s) used to carry out the reported observations and measurements.
- an explicit mention of the biological and chemical entity(ies) that are being measured.
- an explicit mention of the biological and chemical entity(ies) that are altered/varied/perturbed in a controlled manner.
- the exact sample size (n) for each experimental group/condition, given as a number, not a range;
- a description of the sample collection allowing the reader to understand whether the samples represent technical or biological replicates (including how many animals, litters, cultures, etc.).
- a statement of how many times the experiment shown was independently replicated in the laboratory.
- definitions of statistical methods and measures:
  - common tests, such as t-test (please specify whether paired vs. unpaired), simple χ2 tests, Wilcoxon and Mann-Whitney tests, can be unambiguously identified by name only, but more complex techniques should be described in the methods section;
  - are tests one-sided or two-sided?
  - are there adjustments for multiple comparisons?
  - exact statistical test results, e.g., P values = x but not P values < x;
  - definition of 'center values' as median or average;
  - definition of error bars as s.d. or s.e.m.

Please complete ALL of the questions below.
Select "Not Applicable" only when the requested information is not relevant for your study.

Materials

Newly Created Materials	Information included in the manuscript?	In which section is the information available? (Reagents and Tools Table, Materials and Methods, Figures, Data Availability Section)
New materials and reagents need to be available; do any restrictions apply?	Not Applicable	Data Availability Section, Table EV1, Table EV3

Antibodies	Information included in the manuscript?	In which section is the information available? (Reagents and Tools Table, Materials and Methods, Figures, Data Availability Section)
For **antibodies** provide the following information:<br>- Commercial antibodies: RRID (if possible) or supplier name, catalogue number and or/clone number<br>- Non-commercial: RRID or citation	Yes	Table EV4

DNA and RNA sequences	Information included in the manuscript?	In which section is the information available? (Reagents and Tools Table, Materials and Methods, Figures, Data Availability Section)
**Short novel DNA or RNA including primers, probes**: provide the sequences.	Not Applicable	Table EV2

Cell materials	Information included in the manuscript?	In which section is the information available? (Reagents and Tools Table, Materials and Methods, Figures, Data Availability Section)
**Cell lines:** Provide species information, strain. Provide accession number in repository **OR** supplier name, catalog number, clone number, and/**OR** RRID.	Not Applicable	
**Primary cultures:** Provide species, strain, sex of origin, genetic modification status.	Not Applicable	
Report if the cell lines were recently **authenticated** (e.g., by STR profiling) and tested for mycoplasma contamination.	Not Applicable	

Experimental animals	Information included in the manuscript?	In which section is the information available? (Reagents and Tools Table, Materials and Methods, Figures, Data Availability Section)
**Laboratory animals or Model organisms:** Provide species, strain, sex, age, genetic modification status. Provide accession number in repository **OR** supplier name, catalog number, clone number, **OR** RRID.	Not Applicable	
**Animal observed in or captured from the field:** Provide species, sex, and age where possible.	Not Applicable	
Please detail **housing and husbandry conditions**.	Not Applicable	

Plants and microbes	Information included in the manuscript?	In which section is the information available? (Reagents and Tools Table, Materials and Methods, Figures, Data Availability Section)
**Plants:** provide species and strain, ecotype and cultivar where relevant, unique accession number if available, and source (including location for collected wild specimens).	Not Applicable	
**Microbes:** provide species and strain, unique accession number if available, and source.	Yes	Saccharomyces cerevisiae ATCC 201389

Human research participants	Information included in the manuscript?	In which section is the information available? (Reagents and Tools Table, Materials and Methods, Figures, Data Availability Section)
If collected and within the bounds of privacy constraints report on age, sex and gender or ethnicity for all study participants.	Not Applicable	

Core facilities	Information included in the manuscript?	In which section is the information available? (Reagents and Tools Table, Materials and Methods, Figures, Data Availability Section)
If your work benefited from core facilities, was their service mentioned in the acknowledgments section?	Yes	Acknowledgments

Design

Study protocol	Information included in the manuscript?	In which section is the information available? (Reagents and Tools Table, Materials and Methods, Figures, Data Availability Section)
If study protocol has been **pre-registered, provide DOI in the manuscript**. For clinical trials, provide the trial registration number **OR** cite DOI.	Not Applicable	
Report the **clinical trial registration number** (at ClinicalTrials.gov or equivalent), where applicable.	Not Applicable	

Laboratory protocol	Information included in the manuscript?	In which section is the information available? (Reagents and Tools Table, Materials and Methods, Figures, Data Availability Section)
Provide DOI OR other citation details if **external detailed step-by-step protocols** are available.	Not Applicable	

Experimental study design and statistics	Information included in the manuscript?	In which section is the information available? (Reagents and Tools Table, Materials and Methods, Figures, Data Availability Section)
Include a statement about **sample size** estimate even if no statistical methods were used.	Yes	Methods
Were any steps taken to minimize the effects of subjective bias when allocating animals/samples to treatment (e.g. **randomization procedure**)? If yes, have they been described?	Not Applicable	
Include a statement about **blinding** even if no blinding was done.	Not Applicable	
Describe **inclusion/exclusion criteria** if samples or animals were excluded from the analysis. Were the criteria pre-established?<br><br>If sample or data points were omitted from analysis, report if this was due to attrition or intentional exclusion and provide justification.	Not Applicable	
For every figure, are **statistical tests** justified as appropriate? Do the data meet the assumptions of the tests (e.g., normal distribution)? Describe any methods used to assess it. Is there an estimate of variation within each group of data? Is the variance similar between the groups that are being statistically compared?	Not Applicable	

Sample definition and in-laboratory replication	Information included in the manuscript?	In which section is the information available? (Reagents and Tools Table, Materials and Methods, Figures, Data Availability Section)
In the figure legends: state number of times the experiment was **replicated** in laboratory.	Yes	Methods
In the figure legends: define whether data describe **technical or biological replicates**.	Yes	Methods

Ethics

Ethics	Information included in the manuscript?	In which section is the information available? (Reagents and Tools Table, Materials and Methods, Figures, Data Availability Section)
Studies involving **human participants**: State details of **authority granting ethics approval** (IRB or equivalent committee(s), provide reference number for approval.	Not Applicable	
Studies involving **human participants**: Include a statement confirming that **informed consent** was obtained from all subjects and that the experiments conformed to the principles set out in the WMA Declaration of Helsinki and the Department of Health and Human Services Belmont Report.	Not Applicable	
Studies involving **human participants:** For publication of **patient photos**, include a statement confirming that consent to publish was obtained.	Not Applicable	
Studies involving experimental **animals**: State details of **authority granting ethics approval** (IRB or equivalent committee(s), provide reference number for approval. Include a statement of compliance with ethical regulations.	Not Applicable	
Studies involving **specimen and field samples:** State if relevant **permits** obtained, provide details of authority approving study; if none were required, explain why.	Not Applicable	

Dual Use Research of Concern (DURC)	Information included in the manuscript?	In which section is the information available? (Reagents and Tools Table, Materials and Methods, Figures, Data Availability Section)
Could your study fall under dual use research restrictions? Please check biosecurity documents and list of **select agents and toxins** (CDC): https://www.selectagents.gov/sat/list.htm	Not Applicable	
If you used a select agent, is the security level of the lab appropriate and reported in the manuscript?	Not Applicable	
If a study is subject to dual use research of concern regulations, is the name of the **authority granting approval and reference number** for the regulatory approval provided in the manuscript?	Not Applicable	

Reporting

The MDAR framework recommends adoption of discipline-specific guidelines, established and endorsed through community initiatives. Journals have their own policy about requiring specific guidelines and recommendations to complement MDAR.

Adherence to community standards	Information included in the manuscript?	In which section is the information available? (Reagents and Tools Table, Materials and Methods, Figures, Data Availability Section)
State if relevant guidelines or checklists (e.g., **ICMJE, MIBBI, ARRIVE, PRISMA**) have been followed or provided.	Not Applicable	
For **tumor marker prognostic studies,** we recommend that you follow the **REMARK** reporting guidelines (see link list at top right). See author guidelines, under 'Reporting Guidelines'. Please confirm you have followed these guidelines.	Not Applicable	
For **phase II and III randomized controlled trials**, please refer to the **CONSORT** flow diagram (see link list at top right) and submit the CONSORT checklist (see link list at top right) with your submission. See author guidelines, under 'Reporting Guidelines'. Please confirm you have submitted this list.	Not Applicable	

Data Availability

Data availability	Information included in the manuscript?	In which section is the information available? (Reagents and Tools Table, Materials and Methods, Figures, Data Availability Section)
Have **primary datasets** been deposited according to the journal's guidelines (see 'Data Deposition' section) and the respective accession numbers provided in the Data Availability Section?	Yes	Data Availability
Were **human clinical and genomic datasets** deposited in a public access-controlled repository in accordance to ethical obligations to the patients and to the applicable consent agreement?	Not Applicable	
Are **computational models** that are central and integral to a study available without restrictions in a machine-readable form? Were the relevant accession numbers or links provided?	Not Applicable	
If publicly available data were reused, provide the respective **data citations in the reference list**.	Not Applicable	
