# Supplementary material for: The AMIGa complex is at the crossroads of cytochrome c oxidase and ATP synthase biogenesis
Source: EMBO Rep. 2026 Oct 6;27(21):6115–38. doi: 10.1038/s44319-026-00946-7 (PMC13743771; doi:10.1038/s44319-026-00946-7)
Supplement: Supplementary file 2 [file 44319_2026_946_MOESM6_ESM.pdf]

Expanded View Figures

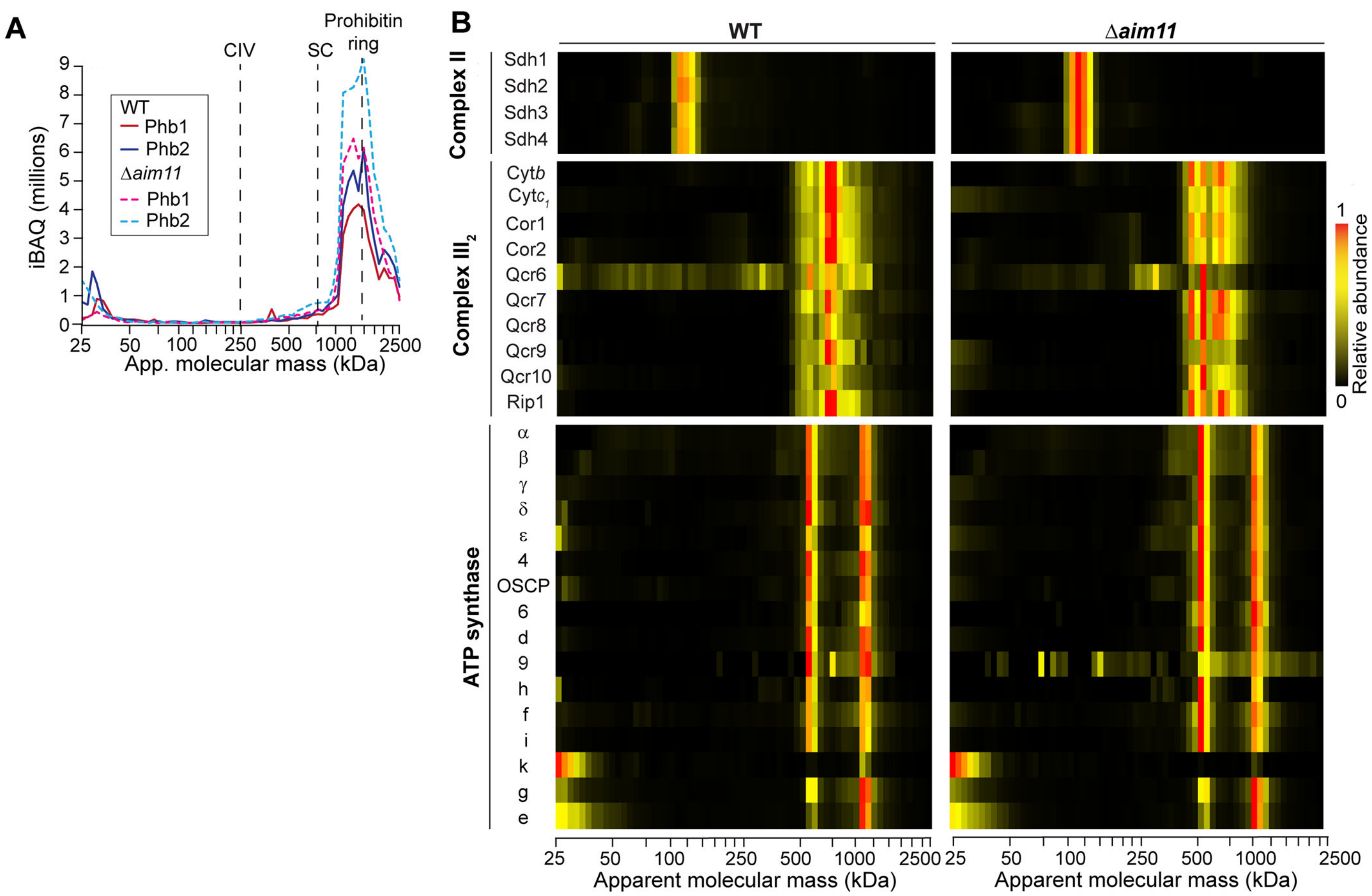

Figure EV1. Absence of Aim11 accumulates prohibitin levels, but does not alter CII, CIII$_2$, or ATP synthase levels.

(**A**) Prohibitin ring (Phb1 and Phb2) complexome migration in WT and *AIM11* null mutant mitochondria. (**B**) Heat maps of the complexome profiling analysis showing the migration patterns of CII, CIII$_2$, and ATP synthase individual subunits.

© The Author(s)

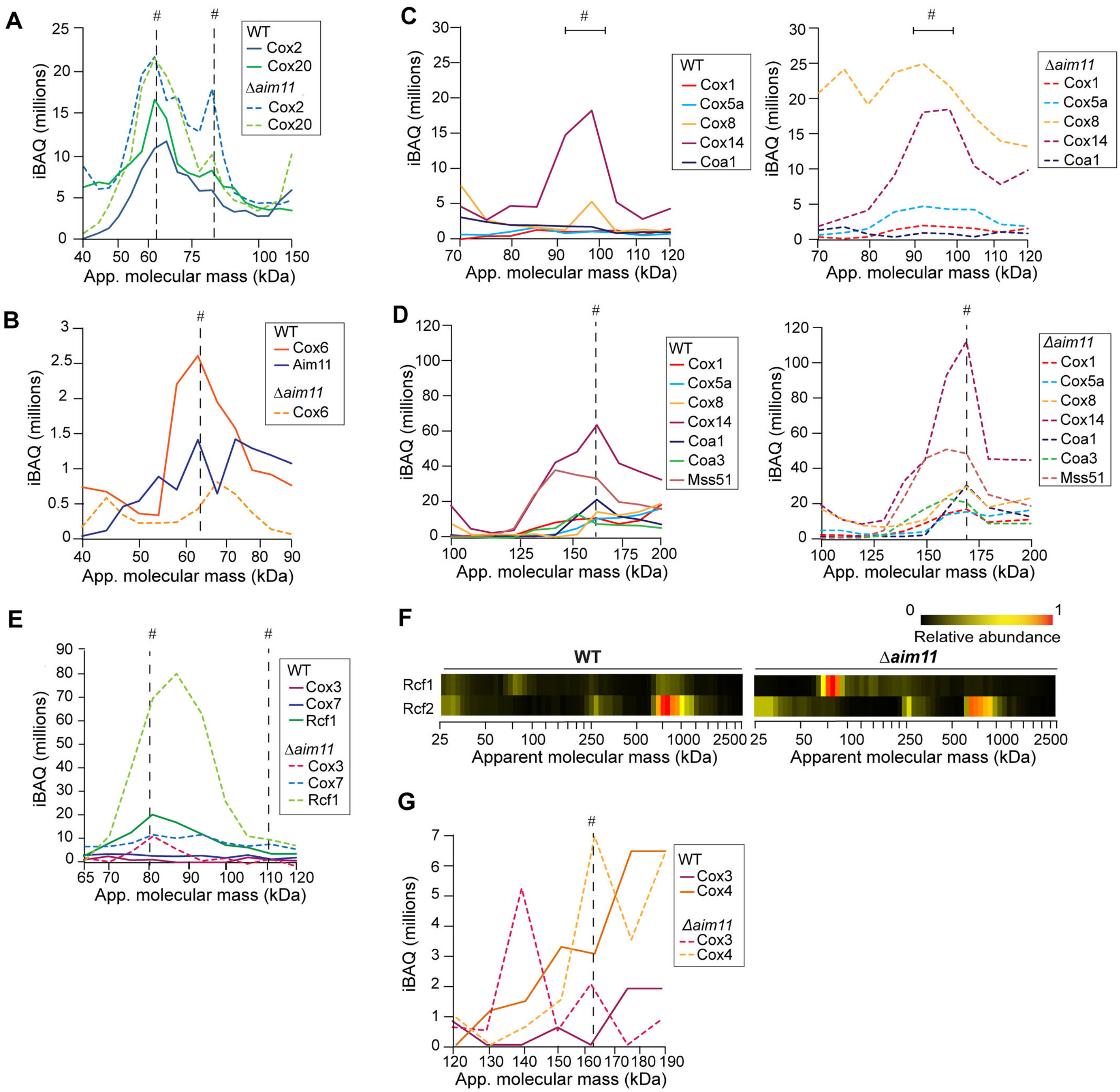

Figure EV2. The absence of Aim11 alters the three CIV assembly modules.

(**A–E**) Abundance and migration patterns of different CIV subunits and assembly factors. The y-axis shows average iBAQ values ($n = 2$) from each protein. (**F**) Heat maps of the complexome profiling analysis showing the migration patterns of Rcf1 and Rcf2. (**G**) Abundance and migration patterns of CIV subunits Cox3 and Cox4. The y-axis shows average iBAQ values ($n = 2$) from each protein.

© The Author(s)

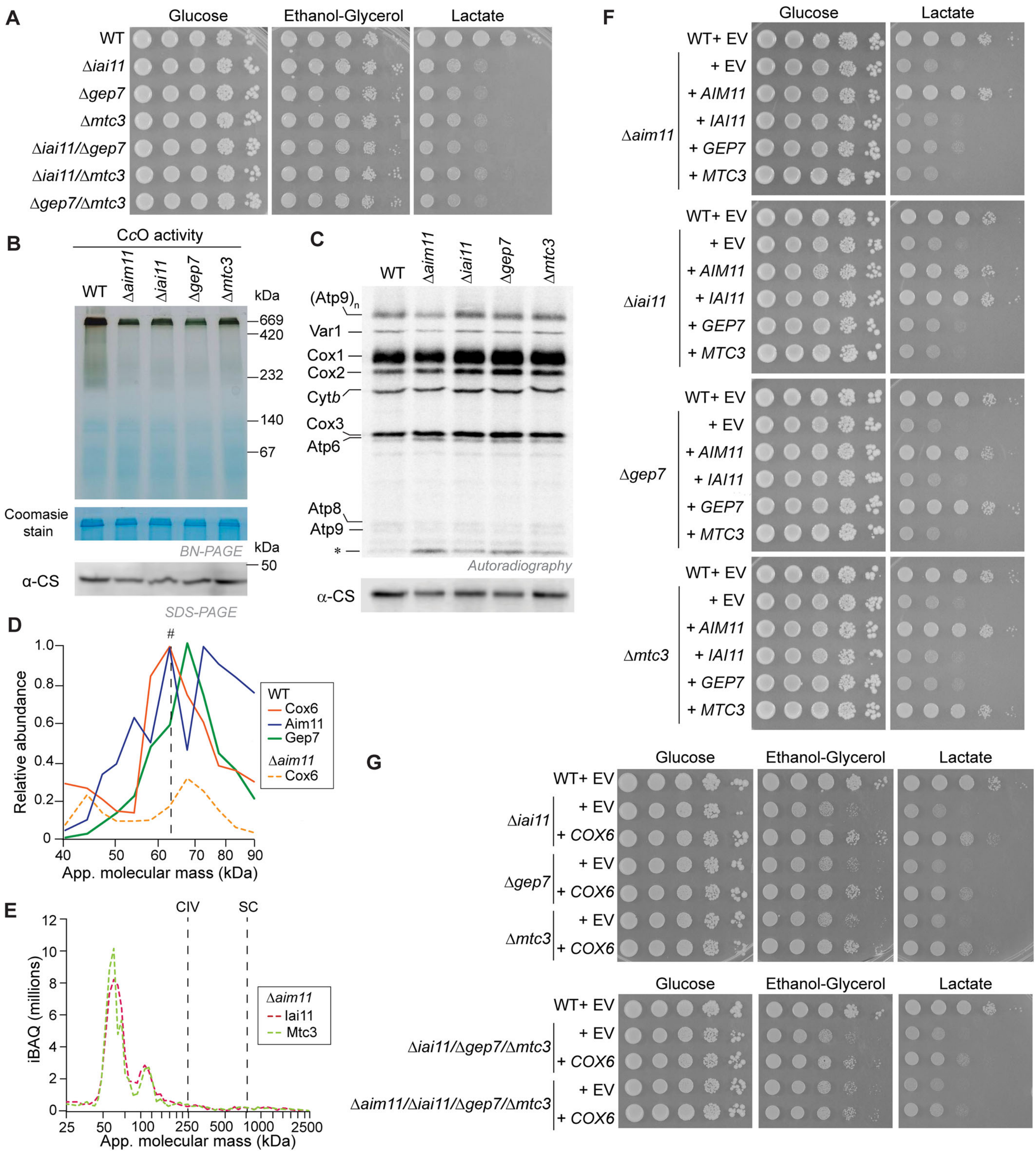
A
Glucose
Ethanol-Glycerol
Lactate
WT
Δiai11
Δgep7
Δmtc3
Δiai11/Δgep7
Δiai11/Δmtc3
Δgep7/Δmtc3
B
CcO activity
WT
Δaim11
Δiai11
Δgep7
Δmtc3
kDa
669
420
232
140
67
Coomasie stain
BN-PAGE
kDa
50
α-CS
SDS-PAGE
C
WT
Δaim11
Δiai11
Δgep7
Δmtc3
(Atp9)n
Var1
Cox1
Cox2
Cytb
Cox3
Atp6
Atp8
Atp9
*
Autoradiography
α-CS
D
#
Relative abundance
1.0
0.8
0.6
0.4
0.2
0
40
50
60
70
80
90
App. molecular mass (kDa)
WT
Cox6
Aim11
Gep7
Δaim11
Cox6
E
CIV
SC
iBAQ (millions)
12
10
8
6
4
2
0
25
50
100
250
500
1000
2500
App. molecular mass (kDa)
Δaim11
Iai11
Mtc3
F
Glucose
Lactate
WT+ EV
Δaim11
Δiai11
Δgep7
Δmtc3
+ EV
+ AIM11
+ IAI11
+ GEP7
+ MTC3
G
Glucose
Ethanol-Glycerol
Lactate
WT+ EV
Δiai11
Δgep7
Δmtc3
+ EV
+ COX6
Δiai11/Δgep7/Δmtc3
Δaim11/Δiai11/Δgep7/Δmtc3

Figure EV3. Aim11 is the core component of the AMIGa complex.

(**A**) Serial dilutions of the indicated strains in fermentative (glucose) and respiratory media (ethanol-glycerol or lactate). Growth in CSM media was shown after 3 days at 30 °C. (**B**) Mitochondria (100 µg) were solubilized with digitonin and separated by BN-PAGE. In-gel staining activity of CIV was performed. A fraction of the sample was separated by SDS-PAGE and transferred to a PVDF membrane for western blot and probed with an antibody against citrate synthase and was used as loading control. (**C**) Mitochondrial translation products from whole cells were labeled with [^{35}S]-methionine in the indicated strains. An unknown translation product was present in all the null mutants (*). (**D**) Complexome profiles of Gep7, Aim11, and Cox6. A comigration signal at around 60 kDa is indicated with the # symbol. This comigration disappeared in the absence of Aim11. (**E**) Complexome profile showing migration patterns of Iai11 and Mtc3 in the absence of Aim11. The apparent molecular mass of Complex IV (CIV) and supercomplexes (SC) is indicated. (**F**) Serial dilutions of the null mutants of AMIGa complex components with the indicated overexpression plasmids. Cells were grown in either fermentative (glucose) or respiratory (lactate) media lacking uracil. Growth shown after 5 days at 30 °C. EV is empty vector. (**G**) Serial dilutions of the single, triple and quadruple *AIM11, IAI11, GEP7*, and *MTC3* null mutant strains with the indicated overexpression plasmids. Cells were grown in either fermentative (glucose) or respiratory (ethanol-glycerol or lactate) media lacking uracil for 5 days at 30 °C. EV is empty vector.

© The Author(s)

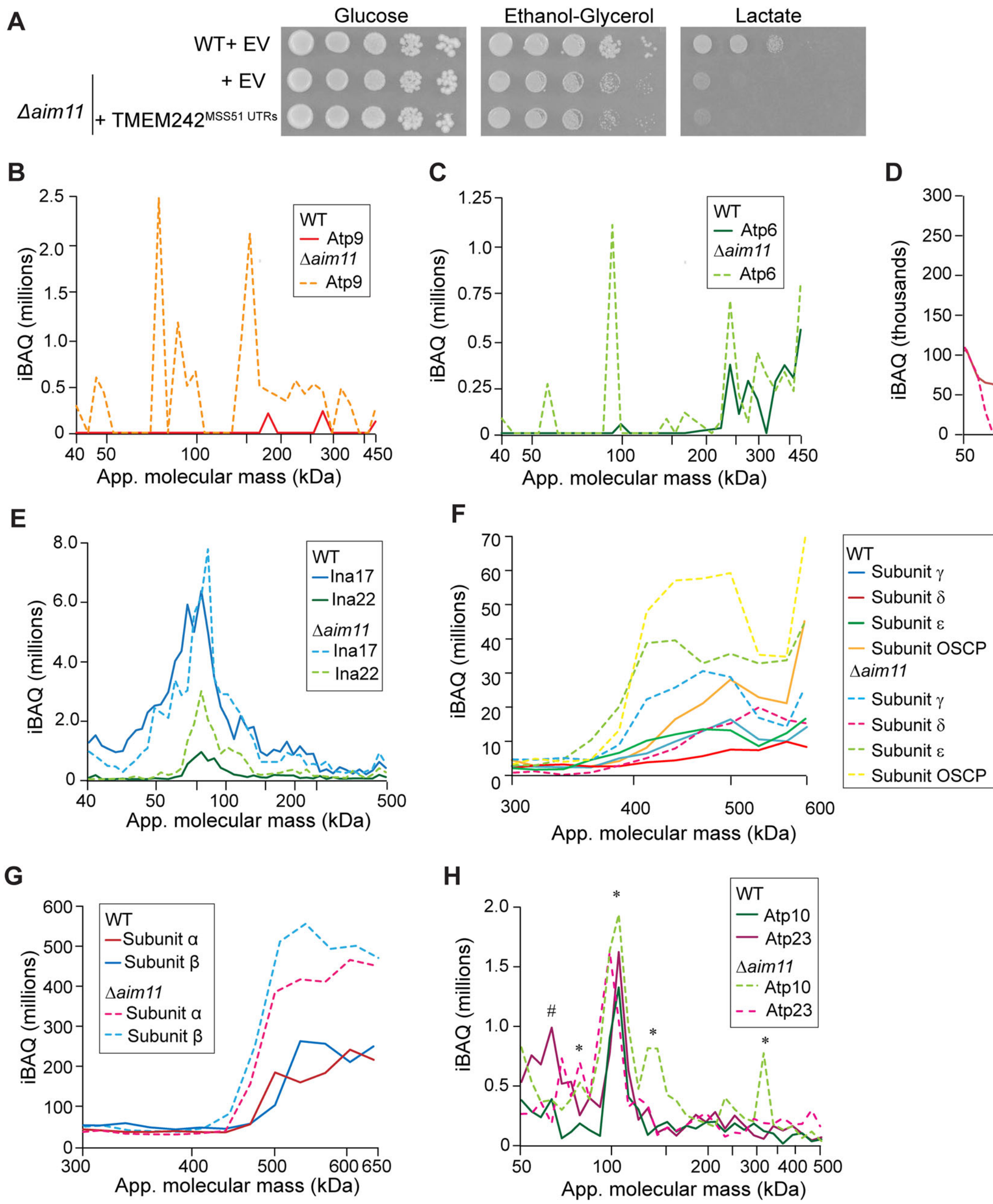

Figure EV4. TMEM242 rescues the respiratory phenotype of a *Δaim11* mutation only when it is overexpressed.

(**A**) Serial dilutions of *Δaim11* cells with the indicated plasmids. *TMEM242* was cloned on a centromeric plasmid with the promoter and terminator sequences of the *MSS51* gene (*TMEM242$^{MSS51\ UTRs}$*). Cells were grown in media lacking uracil with glucose, ethanol-glycerol, or lactate as a carbon source. Cells were grown for 5 days at 30 °C. EV is empty vector. (**B–H**) Complexome migration patterns of the indicated ATP synthase subunits and assembly factors. The y-axis shows average iBAQ values ($n = 2$) from each protein. In (**H**), peaks only observed in WT (#) or *Δaim11*(*) mitochondria are indicated.

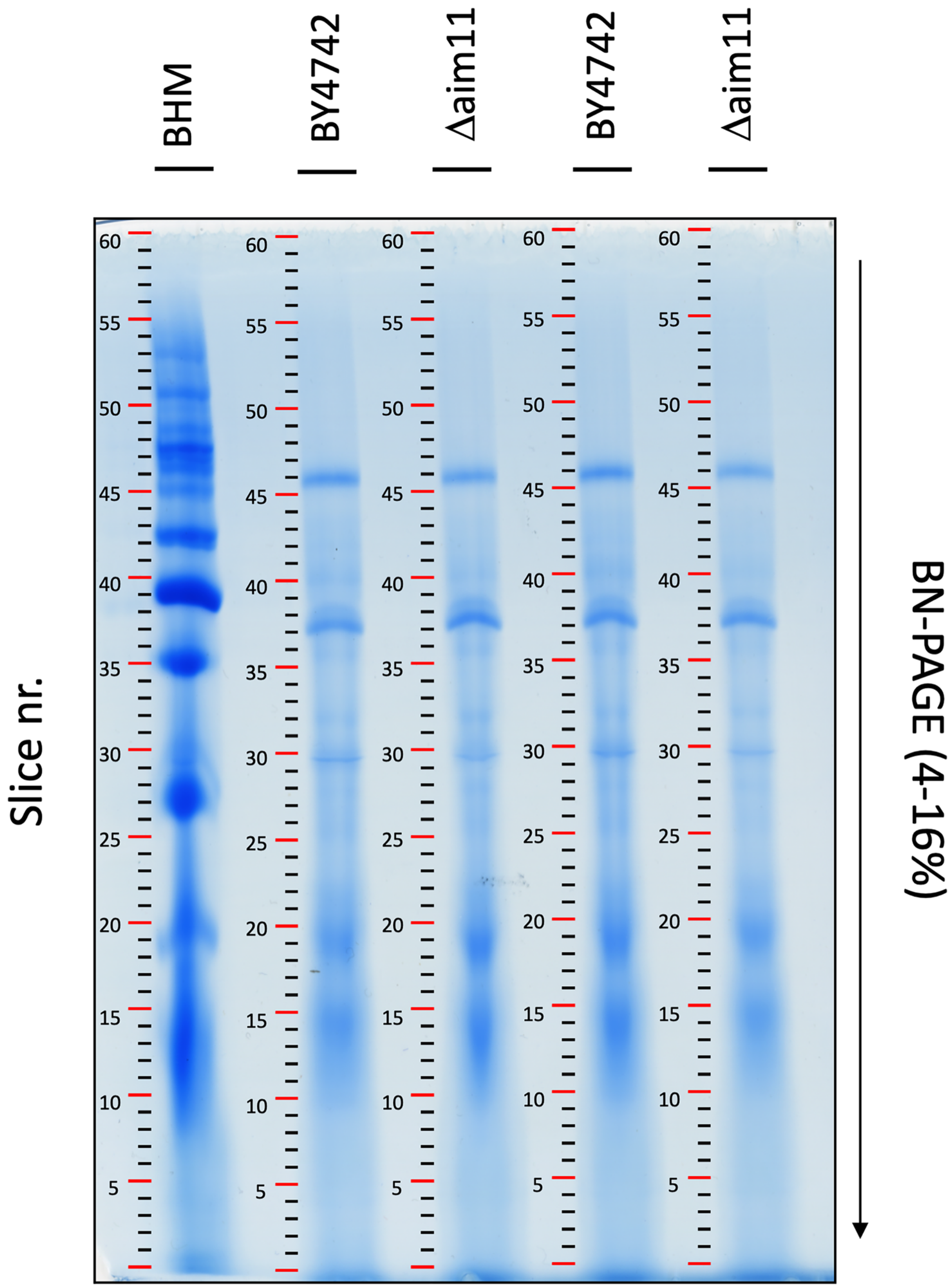

Figure EV5. Sample BN-PAGE gel used for complexome profile.

100 μg of mitochondrial protein were loaded in each lane. BN-PAGE conditions were as described in the Materials and Methods section. Bovine heart mitochondria (BHM) were used as a molecular mass reference.

 © The Author(s)
