# Supplementary material for: The AMIGa complex is at the crossroads of cytochrome c oxidase and ATP synthase biogenesis
Source: EMBO Rep. 2026 Oct 6;27(21):6115–38. doi: 10.1038/s44319-026-00946-7 (PMC13743771; doi:10.1038/s44319-026-00946-7)
Supplement: Supplementary file 3 [file 44319_2026_946_MOESM1_ESM.docx]

**Table EV1.** Yeast strains used in this study.

| Strain | Genotype | REFERENCE |
| --- | --- | --- |
| BY4742  ATCC 201389 | *MATα, his3Δ1, leu2Δ0, lys2Δ0, ura3Δ0, BY4742* | YKO Matα strain collection (Open Biosystems) |
| UP87 | *MATα, his3Δ1, leu2Δ0, lys2Δ0, ura3Δ0, Δaim11::loxP-LEU2-loxP, BY4742* | This study |
| UP91 | *MATα, his3Δ1, leu2Δ0, lys2Δ0, ura3Δ0, Δaim11::loxP-URA3-loxP, BY4742* | This study |
| *Δaim11* | *MATα, his3Δ1, leu2Δ0, lys2Δ0, ura3Δ0, Δaim11::kanMX4, BY4742* | YKO Matα strain collection (Open Biosystems) |
| *Δiai11* | *MATα, his3Δ1, leu2Δ0, lys2Δ0, ura3Δ0, Δiai11::kanMX4, BY4742* | YKO Matα strain collection (Open Biosystems) |
| *Δgep7* | *MATα, his3Δ1, leu2Δ0, lys2Δ0, ura3Δ0, Δgep7::kanMX4, BY4742* | YKO Matα strain collection (Open Biosystems) |
| *Δmtc3* | *MATα, his3Δ1, leu2Δ0, lys2Δ0, ura3Δ0, Δmtc3::kanMX4, BY4742* | YKO Matα strain collection (Open Biosystems) |
| *Δcox6* | *MATα, his3Δ1, leu2Δ0, lys2Δ0, ura3Δ0, Δcox6::kanMX4, BY4742* | YKO Matα strain collection (Open Biosystems) |
| *Δina17* | *MATα, his3Δ1, leu2Δ0, lys2Δ0, ura3Δ0, Δina17::kanMX4, BY4742* | YKO Matα strain collection (Open Biosystems) |
| *Δina22* | *MATα, his3Δ1, leu2Δ0, lys2Δ0, ura3Δ0, Δina22::kanMX4, BY4742* | YKO Matα strain collection (Open Biosystems) |
| UP85 | *MATα, his3Δ1, leu2Δ0, lys2Δ0, ura3Δ0, Δaim11::loxP-LEU2-loxP, Δiai111::kanMX4, BY4742* | This study |
| UP89 | *MATα, his3Δ1, leu2Δ0, lys2Δ0, ura3Δ0, Δaim11::loxP-LEU2-loxP, Δgep7::kanMX4, BY4742* | This study |
| UP135 | *MATα, his3Δ1, leu2Δ0, lys2Δ0, ura3Δ0, Δaim11::loxP-LEU2-loxP, Δmtc3::kanMX4, BY4742* | This study |
| UP227 | *MATα, his3Δ1, leu2Δ0, lys2Δ0, ura3Δ0, Δmtc3::kanMX4, Δgep7::natMX4, BY4742* | This study |
| UP230 | *MATα, his3Δ1, leu2Δ0, lys2Δ0, ura3Δ0, Δmtc3::kanMX4, Δiai11::hphMX4, BY4742* | This study |
| UP231 | *MATα, his3Δ1, leu2Δ0, lys2Δ0, ura3Δ0, Δgep7::kanMX4, Δiai11::hphMX4, BY4742* | This study |
| UP228 | *MATα, his3Δ1, leu2Δ0, lys2Δ0, ura3Δ0, Δaim11::loxP-LEU2-loxP, Δmtc3::kanMX4, Δgep7::natMX4, BY4742* | This study |
| UP234 | *MATα, his3Δ1, leu2Δ0, lys2Δ0, ura3Δ0, Δmtc3::kanMX4, Δgep7::natMX4, Δiai11::hphMX4, BY4742* | This study |
| UP235 | *MATα, his3Δ1, leu2Δ0, lys2Δ0, ura3Δ0, Δaim11::loxP-LEU2-loxP, Δmtc3::kanMX4, Δgep7::natMX4, Δiai11::hphMX4, BY4742* | This study |
| UP120 | *MATα, his3Δ1, leu2Δ0, lys2Δ0, ura3Δ0, AIM11-3xFLAG-URA3-3xFLAG, BY4742* | This study |
| UP121 | *MATα, his3Δ1, leu2Δ0, lys2Δ0, ura3Δ0, AIM11-3xFLAG, BY4742* | This study |
| UP173 | *MATα, his3Δ1, leu2Δ0, lys2Δ0, ura3Δ0, MTC3-3xHA-URA3-3xHA, BY4742* | This study |
| UP177 | *MATα, his3Δ1, leu2Δ0, lys2Δ0, ura3Δ0, MTC3-3xHA, BY4742* | This study |
| UP178 | *MATα, his3Δ1, leu2Δ0, lys2Δ0, ura3Δ0, IAI11-3xV5-URA3-3xV5, BY4742* | This study |
| UP200 | *MATα, his3Δ1, leu2Δ0, lys2Δ0, ura3Δ0, IAI11-3xV5, BY4742* | This study |
| UP174 | *MATα, his3Δ1, leu2Δ0, lys2Δ0, ura3Δ0, GEP7-3xHA-URA3-3xHA, BY4742* | This study |
| UP176 | *MATα, his3Δ1, leu2Δ0, lys2Δ0, ura3Δ0, GEP7-3xHA, BY4742* | This study |
| UP179 | *MATα, his3Δ1, leu2Δ0, lys2Δ0, ura3Δ0, AIM11-3xFLAG, MTC3-3xHA-URA3-3xHA, BY4742* | This study |
| UP180 | *MATα, his3Δ1, leu2Δ0, lys2Δ0, ura3Δ0, AIM11-3xFLAG, MTC3-3xHA, BY4742* | This study |
| UP182 | *MATα, his3Δ1, leu2Δ0, lys2Δ0, ura3Δ0, AIM11-3xFLAG, GEP7-3xHA-URA3-3xHA, BY4742* | This study |
| UP188 | *MATα, his3Δ1, leu2Δ0, lys2Δ0, ura3Δ0, AIM11-3xFLAG, GEP7-3xHA, BY4742* | This study |
| UP197 | *MATα, his3Δ1, leu2Δ0, lys2Δ0, ura3Δ0, AIM11-3xFLAG, IAI11-3xV5-URA3-3xV5, BY4742* | This study |
| UP201 | *MATα, his3Δ1, leu2Δ0, lys2Δ0, ura3Δ0, AIM11-3xFLAG, IAI11-3xV5, BY4742* | This study |
| UP270 | *MATα, his3Δ1, leu2Δ0, lys2Δ0, ura3Δ0, COX6-3xFLAG-URA3-3xFLAG, BY4742* | This study |
| UP285 | *MATα, his3Δ1, leu2Δ0, lys2Δ0, ura3Δ0, COX6-3xFLAG, BY4742* | This study |
| UP294 | *MATα, his3Δ1, leu2Δ0, lys2Δ0, ura3Δ0, COX6-3xFLAG, Δaim11::loxP-URA3-loxP, BY4742* | This study |
| UP217 | *MATα, his3Δ1, leu2Δ0, lys2Δ0, ura3Δ0, Δina17::kanMX4, Δaim11::loxP-URA3-loxP, BY4742* | This study |
| UP225 | *MATα, his3Δ1, leu2Δ0, lys2Δ0, ura3Δ0, Δina22::kanMX4, Δaim11::loxP-URA3-loxP, BY4742* | This study |
| NB40 3c rho^0^ | *MATa*, *arg8::hisG, leu2-3,112, lys2, ura3-52, his3ΔHindIII, D273-10B (ρ^0^)* | (Bonnefoy et al., 2001) |

In parenthesis is the mitochondrial genotype.

**References for Tables EV**

Bonnefoy, N., Bsat, N., & Fox, T. D. (2001). Mitochondrial Translation of *Saccharomyces cerevisiae COX2* mRNA Is Controlled by the Nucleotide Sequence Specifying the Pre-Cox2p Leader Peptide. *Molecular and Cellular Biology*, *21*(7), 2359–2372. https://doi.org/10.1128/MCB.21.7.2359-2372.2001

García-Villegas, R., Camacho-Villasana, Y., Shingú-Vázquez, M. Á., Cabrera-Orefice, A., Uribe-Carvajal, S., Fox, T. D., & Pérez-Martínez, X. (2017). The Cox1 C-terminal domain is a central regulator of cytochrome c oxidase biogenesis in yeast mitochondria. *Journal of Biological Chemistry*, *292*(26), 10912–10925. https://doi.org/10.1074/jbc.M116.773077

Goldstein, A. L., & McCusker, J. H. (1999). Three new dominant drug resistance cassettes for gene disruption in *Saccharomyces cerevisiae*. *Yeast*, *15*(14), 1541–1553. https://doi.org/10.1002/(SICI)1097-0061(199910)15:14<1541::AID-YEA476>3.0.CO;2-K

Gueldener, U. (2002). A second set of loxP marker cassettes for Cre-mediated multiple gene knockouts in budding yeast. *Nucleic Acids Research*, *30*(6), 23e–223. https://doi.org/10.1093/nar/30.6.e23

Hill, J. E., Myers, A. M., Koerner, T. J., & Tzagoloff, A. (1986). Yeast/ *E. coli* shuttle vectors with multiple unique restriction sites. *Yeast*, *2*(3), 163–167. https://doi.org/10.1002/yea.320020304

Moqtaderi, Z., & Struhl, K. (2008). Expanding the repertoire of plasmids for PCR‐mediated epitope tagging in yeast. *Yeast*, *25*(4), 287–292. https://doi.org/10.1002/yea.1581

Schneider, B. L., Seufert, W., Steiner, B., Yang, Q. H., & Futcher, A. B. (1995). Use of polymerase chain reaction epitope tagging for protein tagging in *Saccharomyces cerevisiae*. *Yeast*, *11*(13), 1265–1274. https://doi.org/10.1002/yea.320111306

Westermann, B., & Neupert, W. (2000). Mitochondria-targeted green fluorescent proteins: convenient tools for the study of organelle biogenesis in *Saccharomyces cerevisiae*. *Yeast*, *16*(15), 1421–1427. https://doi.org/10.1002/1097-0061(200011)16:15<1421::AID-YEA624>3.0.CO;2-U
