# Supplementary material for: The AMIGa complex is at the crossroads of cytochrome c oxidase and ATP synthase biogenesis
Source: EMBO Rep. 2026 Oct 6;27(21):6115–38. doi: 10.1038/s44319-026-00946-7 (PMC13743771; doi:10.1038/s44319-026-00946-7)
Supplement: Supplementary file 4 [file 44319_2026_946_MOESM2_ESM.docx]

**Table EV2.** Oligonucleotides used in the present work.

| Oligonucleotide | Sequence (5’-3’) | Use |
| --- | --- | --- |
| AIM11 Fw1 | CCGAGCTCGCGGATTGATCGGTTCATGGCAATCTCATGGTCCC | To amplify *AIM11* gene ~300 bp upstream and downstream the coding sequence. |
| AIM11 Rv1 | GGCTGCAGCGCGGGCTACTCTGTTTTGCACTGTGAGGTATGCTC |  |
| AIM11 Fw2 | GGATGGGGAACTTGATTCTTTGACGGGTTTGTCAGCAATTAGTACGACTATCCCCAGCTGAAGCTTCGTACGCTGC | Generate *URA3* or *LEU2* cassete for *AIM11* deletion by homologous recombination. For pUG72 or pUG73 plasmids. |
| AIM11 Rv2 | GCCGGCAACGGCTCGTAGTTATACCATTATTTACAGTTTAAAGAGATTAAGCCCCGCATAGGCCACTAGTGGATCTG |  |
| AIM11 S1 | GGAATGAAGCCATGTCAGCGTTAACGTATGCATCC | Forward primer to sequence *AIM11* constructions. |
| AIM1pMPY F | ACTAACTAAGCAATTGCAAG ACCTCCTGTC GAGCGAAAAC AACAAGAGGGAACAAAAGCTGG | To tag Aim11 at the C-terminus. To Use pMPY-HAx3, ZM467 or ZM474 plasmid. |
| AIM11 pMPY R | TATACCATTATTTACAGTTTAAAGAGATTAAGCCAATGCGTAGTGCTACTGTAGGGCGAATTGGG |  |
| TMEM242 F | GTGAAGCTTATGGAGACAGCGGGCGCTGCAACTGG | To clone TMEM242-FLAG from cDNA by fusion PCR. |
| TMEM242 P2 | CCTAAAGCCTTCCAGACTGCGAAGCTAATC |  |
| TMEM242 P3 | GATTAGCTTCGCAGTCTGGAAGGCTTTAGG |  |
| TMEM242 R | ACTGCGGCCGCTCACTTGTCGTCATCGTCTTTGTAGTCCGCCGCTTTGGATTTCAATGTTTCCTCCC |  |
| IAI11 F | TTGCCTCCTTGAGACACTTGTTCAAAGC | To amplify *IAI11* gene ~300 bp upstream and downstream the coding sequence. |
| IAI11 R | TCATCCGTTTGAGAAGCATAATAGG |  |
| IAI11 F2 | AGCAAGTAGTGGACAGCTTAGTTAAGACACACAATTCATCTCTTTGTAAA AGGGAACAAAAGCTGG | To tag Iai11 at the C-terminus. Use pMPY-HAx3, ZM467 or ZM474 plasmid. |
| IAI11 R2 | ATATAAGTAGTGGAATATATTATTGAAACTACTACTATGGTATAACACTACTGTAGGGCGAATTGGG |  |
| IAI11 S1 | GGTATATTTGGTATGGGCATCACAGG | Forward primer to sequence *IAI11* constructions. |
| IAI11 F3 | GTAGTACTTGTCTTATCTTGCTTTTGTTCAACTGCACTTGTAAATCAGTGACCAGCTGAAGCTTCGTACGC | Generate *URA3*, *nat*MX4, *hph*MX4 or *kan*MX4 cassete for *IAI11* deletion by homologous recombination. For pAG25, pAG32, pAG60 or pAFA-*kan* plasmids. |
| IAI11 R3 | CACATATAAGTAGTGGAATATATTATTGAAACTACTACTATGGTATAACACGATGAATTCGAGCTCGTT |  |
| GEP7 F | CCACCTCAAAGTCCAAGTAATTGTCC | To amplify *GEP7* gene ~300 bp upstream and downstream the coding sequence. |
| GEP7 R | CATCGTGAGTTTCTTTCTCTATCGATCC |  |
| GEP7 F2 | AACAAAAAACGAAGGAGCTGCCCGCACAGAAGAGTACTGTAATATCAGAAAGGGAACAAAAGCTGG | To tag Gep7 at the C-terminal. Use pMPY-HAx3, ZM467 or ZM474 plasmid. |
| GEP7 R2 | TGAGTGGCACTTTTAACTGCAAGAATGAAGAGTGACCCCATTTTTTTTTAACTGTAGGGCGAATTGGG |  |
| GEP7 F3 | TATATACACATGTTCTCGCTAAAATCAGTTAAAAGGTCCGATTGAGCCAGCTGAAGCTTCGTACGC | Generate *URA3*, *nat*MX4, *hph*MX4 or *kan*MX4 cassete for *GEP7* deletion by homologous recombination. For pAG25, pAG32, pAG60 or pAFA-*kan* plasmids. |
| GEP7 R3 | CACATATAAGTAGTGGAATATATTATTGAAACTACTACTATGGTATAACACGATGAATTCGAGCTCGTT |  |
| GEP7 S1 | CTCAATTTGTCCTGACAAACTCTTCTCC | Forward primer to sequence *GEP7* constructions. |
| MTC3 F | GGATAAATGGCTGGAAGCTCTTTGAGG | To amplify *MTC3* gene ~300 bp upstream and downstream the coding sequence. |
| MTC3 R | CTAAGAGTGACTAATGTAGGTAAGTGC |  |
| MTC3 F2 | ATAATTGGAAACAAGATAAAAAGCTAGAGGAACAATTAAGGGATCTTGTAAGGGAACAAAAGCTGG | To tag Mtc3 at the C-terminus. Use pMPY-HAx3, ZM467 or ZM474 plasmid. |
| MTC3 R2 | GTACTCTGCGCAAGCGCATATATATATATACTATTGTTGCTTCCAATTTACTGTAGGGCGAATTGGG |  |
| MTC3 F3 | AAGACCACCCAAAAGATATCATTTCAACATAGGTAGAAATAAGGCAGAAGCCAGCTGAAGCTTCGTACGC | Generate *URA3*, *nat*MX4, *hph*MX4 or *kan*MX4 cassete for *MTC3* deletion by homologous recombination. For pAG25, pAG32, pAG60 or pAFA-*kan* plasmids. |
| MTC3 R3 | AACGTACTCTGCGCAAGCGCATATATATATATACTATTGTTGCTTCCAATTCGATGAATTCGAGCTCGTT |  |
| MTC3 S1 | CAAGGAATACTTACGATGGAGGATGC | Forward primer to sequence *MTC3* constructions. |
| COX6 F1 | GCCAATCAGGGCCCCGCGCGTTATTTC | To amplify *COX6* gene ~300 bp upstream and downstream the coding sequence. |
| COX6 R1 | ATATTAAAGGTAATCTGTGACCAGCCC |  |
| MSS51 F13 | CCGCTCGAGTCTAGAGCCACCCAGGTCGCAATGCCCTTCC | To amplify and fuse *MSS51* 5’ and 3’ UTRs. |
| MSS51 R10 | CCCAAGCTTAGTTCTGGAAGTACAAGTTTGCC |  |
| MSS51 F14 | GGCAAACTTGTACTTCCAGAACTAAAGCTTGCGGCCGCTTATCGGGAGGCCATGCCCAACTTTC |  |
| MSS51 R11 | CGAGCTCGCCTAATATAGTCCGAAAAGGAAAGG |  |
| NAT Rev | GTTGTTTATGTTCGGATGTG | Reverse primer to confirm recombination of *natMX4* cassete in the mutant. |
| HYG Rev | GCAATCGCGCATATGAAATC | Reverse primer to confirm recombination of *hphMX4* cassete in the mutant. |
| KanB | CTGCAGCGAGGAGCCGTAAT | Reverse primer to confirm recombination of kanMX4 cassete in the mutant. |
| ADH1 F1 | CGTCATTGTTCTCGTTCCCTTTCTTCC | To sequence construction in pVT-100 plasmid. |
| ADH1 R1 | GAGTCACTTTAAAATTTGTATACAC |  |
| T7 | TAATACGACTCACTATAGGG | To sequence construction in pGem-T Easy |
| SP6 | ATTTAGGTGACACTATAGAA |  |
| M13 F | TGTAAAACGACGGCCAGT | To sequence construction in pRS416. |
| M13 R | CAGGAAACAGCTATGAC |  |
