# Supplementary material for: The AMIGa complex is at the crossroads of cytochrome c oxidase and ATP synthase biogenesis
Source: EMBO Rep. 2026 Oct 6;27(21):6115–38. doi: 10.1038/s44319-026-00946-7 (PMC13743771; doi:10.1038/s44319-026-00946-7)
Supplement: Supplementary file 5 [file 44319_2026_946_MOESM3_ESM.docx]

**Table EV3.** Plasmids used or constructed in the present study.

| Plasmid | Relevant characteristics | REF |
| --- | --- | --- |
| pUP10 | TMEM242-FLAG in pVT100U. Under *ADH* promoter and terminator. | This study |
| pUP17 | *AIM11-3xFLAG* in pGem-T Easy*.* | This study |
| pUP18 | *AIM11-3xFLAG* in YEp352. | This study |
| pUP13 | *IAI11-3xV5* in pGem-T Easy. | This study |
| pUP14 | *IAI11-3xV5* in YEp352. | This study |
| pUP12 | *GEP7-3xHA* in pGem-T Easy. | This study |
| pUP16 | *GEP7-3xHA* in YEp352. | This study |
| pUP11 | *MTC3-3xHA* in pGem-T Easy. | This study |
| pUP15 | *MTC3-3xHA* in YEp352. | This study |
| pUP28 | *COX6* in pGem-T Easy*.* | This study |
| pUP29 | *COX6* in YEp352. | This study |
| pYC106 | *MSS51* 3’ and 5’ UTRs in pGEM-T Easy. | This study |
| pYC107 | *MSS51* 3’ and 5’ UTRs in pRS416. | This study |
| pUP30 | TMEM242 with *MSS51* 3’ and 5’ UTRs in pRS416. | This study |
