# Supplementary material for: The AMIGa complex is at the crossroads of cytochrome c oxidase and ATP synthase biogenesis
Source: EMBO Rep. 2026 Oct 6;27(21):6115–38. doi: 10.1038/s44319-026-00946-7 (PMC13743771; doi:10.1038/s44319-026-00946-7)
Supplement: Supplementary file 6 [file 44319_2026_946_MOESM4_ESM.docx]

**Table EV4.** Primary antibodies used in the present work

| Antibody | Dilution | Secondary | Source |
| --- | --- | --- | --- |
| α-Cox1 | 1: 40000 | Rabbit | (García-Villegas et al., 2017) |
| α-Cox2 | 1:2000 | Mouse | Mitoscience |
| α-Cox3 | 1:2000 | Mouse | Mitoscience |
| α-CS | 1:1000 | Rabbit | Gift for XPM from Dr. Thomas Fox |
| α-Atp6 | 1:2000 | Rabbit | Gift for XPM from Dr. Soledad Funes |
| α-Cyt*b* | 1:1000 | Rabbit | Gift for XPM from Dr. Rosemary Stuart |
| α-Rip1 | 1:1000 | Rabbit | Gift for XPM from Dr. Rosemary Stuart |
